# Induction of p53-mediated apoptosis by azacitidine in patient-derived xenograft follicular helper T-cell lymphoma model

**DOI:** 10.1038/s41375-025-02628-0

**Published:** 2025-05-20

**Authors:** Gamze Tari Crochet, Selcen Ari-Yuka, Anja Fischer, Mohamed Chour, Alexis Claudel, Nouhoum Sako, Cyrielle Robe, Julie Naudet, Alexis Gonon, Diana Laure Mboumba, Nicolas Ortonne, Vincent Alcazer, Marie-Hélène Delfau-Larue, Reiner Siebert, Philippe Gaulard, François Lemonnier

**Affiliations:** 1 Paris Est-Créteil University, Créteil, France; 2 Mondor Biomedical Research Institute, Team Ortonne, INSERM U955, Créteil, France; 3 Yildiz Technical University, Department of Bioengineering, Istanbul, Turkey; 4 Institute of Human Genetics, Ulm University and Ulm University Medical Center, Ulm, Germany; 5 International Infectiology Research Center, CIRI, INSERM U1111 - CNRS UMR5308, University of Lyon 1, Pierre Bénite, France; 6 Ecole Normal Supérieure de Lyon, Department of Biology, Lyon, France; 7 Henri Mondor Hospital, Department of Hematology and Immunobiology, Créteil, France; 8 Henri Mondor Hospital, Department of Pathology, Créteil, France; 9 Centre Hospitalier Lyon Sud, Department of Clinical Hematology, Pierre Bénite, France; 10 Henri Mondor Hospital, Department of Lymphoid Hematology, Créteil, France

**Keywords:** Cancer models, Preclinical research, T-cell lymphoma

## Abstract

Follicular helper T-cell lymphoma (TFHL) is the most common non-cutaneous T-cell lymphoma in the Western world and is associated with a poor prognosis. Neoplastic cells rely heavily on the tumor microenvironment, demonstrated by the absence of TFHL-derived cell lines, which hinders therapeutic progress. To overcome this limitation, we developed and characterized patient-derived xenograft TFHL (TFHL-PDXs). Fifteen TFHLs were implanted into immunodeficient mice, generating nine PDXs. The tumor microenvironment was detected in the first passage but progressively disappeared in subsequent passages. *TET2* mutations persisted in all cases and TFHL-specific mutations were observed in most. The models were treated with azacitidine and patient sensitivity was fully recapitulated. To elucidate the mechanism of action of azacitidine, we analyzed the differences in DNA methylation and gene expression in six TFHL-PDX models. Global DNA hypomethylation occurred in azacitidine-treated cells in drug-sensitive models but not in the resistant ones. DNA hypomethylation was associated with global upregulation of gene expression, including that of various cancer-related pathways, suggestive of p53-pathway-mediated cytotoxicity. Overall, the PDXs recapitulated TFHL features and exhibited sensitivity to azacitidine. They also made it possible to decipher the mechanism responsible for the effect of azacitidine, revealing the activation of p53-mediated apoptosis associated with DNA hypomethylation.

## Introduction

Follicular helper T-cell lymphoma (TFHL), the most common type of non-cutaneous T-cell lymphoma in Western countries [1], is associated with immune deregulation, resistance to conventional chemotherapy, and poor outcomes. According to recently updated classifications [2, 3], TFHL is divided into three subtypes: angioimmunoblastic-type (AITL), follicular-type, and TFHL not otherwise specified (NOS). AITL is the most common subtype and is characterized by a prominent tumor microenvironment (TME) consisting of a stromal component, with follicular dendritic cell (FDC) expansion and hyperplastic postcapillary venules, and a hematopoietic component, comprised of reactive CD4^+^ and CD8^+^ T-cells, plasma cells, eosinophils, and commonly enlarged Epstein-Barr virus (EBV)-infected B cells. The usually prevalent TME [4] likely influences tumor transformation and maintenance. TFHL harbors mutations in genes encoding epigenetic regulators, such as *TET2* (up to 80–90%) [5], *DNMT3A* (30%) [6], and *IDH2* (20–30%) [7], and those involved in T-cell signaling, including RHOA pG17V (up to 60%) [8, 9], *VAV1*, *PLCG1*, and CD28 [10]. *TET2* and *DNMT3A* mutations can occur in hematopoietic progenitor cells, driving clonal hematopoiesis and tumor transformation [11].

Recurrent mutations in epigenetic regulators in TFHL provide a rationale for the use of epigenetic-targeting drugs. The better efficacy of histone deacetylase inhibitors, such as romidepsin, in TFHL than in other PTCLs may indicate the importance of epigenetic modifications induced by this compound [12]. The high frequency of mutations in *DNMT3A* and *TET2*, two genes encoding enzymes that regulate cytosine methylation and hydroxymethylation, also provides a rationale for targeting DNA methylation with DNMT inhibitors, such as azacitidine, in TFHL. An initial report of 12 patients treated with oral azacitidine showed promising results [13]. Although the ORACLE study, which was a phase 3 trial comparing oral azacitidine to the investigator’s choice, did not meet its primary endpoint, it showed prolonged survival of patients receiving azacitidine, with a favorable safety profile, suggesting that azacitidine could be combined with other agents [14]. However, its mechanism of action is unclear, in particular, whether it acts through DNA demethylation of tumor suppressor genes, reactivation of endogenous retroviruses, or cytotoxic effects. The identification of such a mechanism could facilitate better drug combinations.

A limitation of preclinical studies in TFHL is the lack of appropriate models, as no representative *invitro* models have yet been derived. To study the effect of azacitidine, we developed several models of patient-derived xenograft TFHL (TFHL-PDXs) and used them to decipher the effect of azacitidine on TFHL.

## Materials and Methods

### Ethics approval and consent to participate for patients and animals

Lymph-node tumor biopsies from TFHL patients of the Henri Mondor University Hospital, Créteil, France, were collected according to Personal Protection Committee of Ile de France V-approved protocol #2015-A00342-47 (see Table 1) and informed consent was obtained from all subjects.

**Table 1:** Patient derived xenograft (PDX) information.

Source	Age	Gender	Treatment	Current Status	Patient Mutational Profile	Patient Aza Response	PDX	Engrafted Material	Median Survival	PDX Mutational Profile	PDX Aza Response
**Patient 1**	45	Male	CHOP E + ICE + BV+ Gemcitabine	Dead with disease	WT	NA	PDX1	Relapse PBMC	116	WT	NA
**Patient 6**	66	Male	CHOP + 5-aza+ ICE+ Gemcitabine+ BV+ Idelalisib+ Thalidomide	Dead with disease	*DNMTA3A mut; TET2 mut; TET2 mut; RHOA mut; TP53WT*	PD	PDX6	Relapse Biopsy	27	*DNMTA3A mut; TET2 mut; TET2 mut; RHOA mut; TP53 WT*	NA
							PDX6 BIS	Relapse Biopsy	51	*DNMTA3A mut; TET2 mut; TET2 mut; TP53 NA*	Resistant
**Patient 11**	71	Female	CHOP + CC486 + GEMOX	Dead with disease	*DNMT3A mut; TET2 mut; TET2 mut; RHOA mut; CD28 mut; IDH2 mut; IDH2 mut; TP53 WT*	SD	PDX11	Diagnosis Biopsy	81	*DNMT3A mut; TET2 mut; TET2 mut; RHOA mut; TP53 WT*	Sensitive
**Patient 12**	42	Male	CHOP E + DHAC + CC486 + CC220 +allo SCT	Alive without disease	*TET2 mut;TET2 mut; RHOA mut; TP53 NA*	CR	PDX12	Relapse Biopsy	23	*TET2 mut;TET2 mut; RHOA mut; TP53 NA*	Sensitive
**Patient 13**	56	Male	CHOP E+ DHAx+ auto SCT + ICE + 5-aza+ Venetoclax	Dead with disease	*DNMT3A mut; TET2 mut; TET2 mut; RHOA mut; CD28 mut; TP53 NA*	PD	PDX13	Relapes Biopsy	30	*DNMT3A mut;TET2 mut; TET2 mut; RHOA mut; TP53 NA*	Resistant
**Patient 16**	66	Female	CHOP + 5-aza	Dead with disease	*DNMT3A mut; TET2 mut; TET2 mut; RHOA mut; TP53 NA*	NA	PDX16	Relapse Biopsy	65	*DNMT3A mut; TET2 mut; TET2 mut; TP53 NA*	NA
**Patient 18**	76	Male	CHOP + 5-aza	Dead with disease	*TET2 mut; TET2 mut; VAV1 mut; TP53 WT*	CR	PDX18	Relapse Biopsy	53	*TET2 mut; TET2 mut; VAV1 mut; TP53 WT*	Sensitive
**Patient 18**	78	Male	CHOP + 5-aza + Lacutamab+ BV+ Bendamustine+ Ruxolitinib	Dead with disease	*TET2 mut; TET2 mut; VAV1 mut; TP53 WT*	CR-PD	PDX18BIS	Relapse Biopsy	39	*TET2 mut; TET2 mut; VAV1 mut; TP53 WT*	Resistant
**Patient 24**	77	Male	5-aza	Alive without disease	*TET2 mut; TET2 mut; RHOA mut; TP53 WT*	CR	PDX24	Diagnosis Biopsy	40	*TET2 mut; TET2 mut; RHOA mut; TP53 WT*	Sensitive
**Patient 26**	75	Female	CHOP + 5-aza	Alive without disease	*DNMT3A mut; TET2 mut; TET2 mut; RHOA mut; IDH2 mut; PLCG1 mut; TP53 WT*	NA	PDX26	Diagnosis Biopsy	47	*DNMT3A mut; TET2 mut; TET2 mut; RHOA mut; IDH2 mut; TP53 WT*	NA

*5-aza* azacitidine, *BV* brentuximab vedotin, *CC200* iberdomide, *CC486* oral azacytidine, *CHOP* cyclophosphamide, doxorubicin, vincristine, prednisone, *CHOP E* CHOP + etoposide, *CR* complete response, *DHAC* dexamethasone, *GEMOX* gemcitabine, oxaliplatin, *ICE* ifosfamide, carboplatin, etoposide, *mut* mutated, *NA* not available, *PD* progressive disease, *SCT* stem cell transplant, *SD* stable disease, *WT* wildtype.

Four-to-six-week-old NOD.Cg-*Prkdc*^*scid*^ *Il2rg*^*tm1Wjl*^/SzJ (NSG^TM^) mice were purchased from Charles River Laboratories (Saint Germain Nuelles, France) or kindly provided by Janvier-Labs (Le Genest-Saint-Isle, France) and bred and handled according to Institutional Animal Care and Use Committee-approved protocol APAFIS#16131-2018071614237737v4 of the Mondor Biomedical Research Institute.

All methods were performed in accordance with the relevant guidelines and regulations.

### PDX generation

Fresh or frozen lymph-node biopsies of TFHL patients were implanted into NSG^TM^ mice subcutaneously under anesthesia, considered Passage 1 (P1). The mice were euthanized at the humane endpoint [15], dissected, and the tumor cells were isolated from involved organs (see Supplementary Materials and Methods). Subsequently, 5–10 × 10^6^ tumor cells of P1 were implanted intraperitoneally into new NSG mice, considered as Passage 2 (P2). PDX generation continued over several passages, considered as P3, P4, etc.

### Flow cytometry

PDX cells were stained with antibodies against human CD45, CD19, CD3, CD4, CD8, ICOS, and PD1 and treated with VersaLyse. Expression was evaluated using an LSR Fortessa X20 flow cytometer (BD Biosciences, USA) and the results were analyzed using FlowJo software.

### Mutational profiling by next-generation sequencing

DNA extracted from the FFPE biopsies of TFHL patients or PDX mice was used for targeted deep sequencing [16, 17] using a customized panel covering genes involved in TFHL (TET2, DNMT3A, IDH2, RHOA, CD28, and others) as previously described [18] (see Supplementary Materials and Methods).

### Targeted Capture T-Cell Receptor Next Generation Sequencing

DNA extracted from the FFPE biopsies of TFHLpatients or PDX mice was used for studying T-cell receptor gene rearrangements. A T-cell receptor capture panel including all V(D)J segments of alpha beta gamma delta chain genes was used. The capture panel consisted of 1322 agilent Sureselect biotinylated baits targeting the 3’100 bp of all TCR V gene regions, and 837 biotinylated baits targeting the 5’ 100 bp of all TCR J gene regions as annotated by IMGT data base https://clicktime.symantec.com/3ZHTCSiunN8mg1iRCXthZX6H2?u=www.imgt.org.

Briefly, DNA was fragmented (180–250 bp) by enzymatic digestion and Agilent DNA libraries were generated from 10–200 ng of fragmented DNA using Sureselect XTHS2 target enrichment kit. Libraries were sequenced on an Illumina NextSeq2000 instrument, and FASQ were analyzed using Vidjil, a Web Platform for Analysis of High-Throughput Repertoire Sequencing. The dominant T-cell clone identified in patient’s biopsies was tracked https://www.vidjil.org/ [19].

### Azacitidine treatment

To study the efficacy of azacitidine, PDX mice received 2.5 mg/kg azacitidine (Vidaza) (Zentiva, Czech Republic) or vehicle (normal saline) by intraperitoneal injection every two days for a total of five doses after circulating tumor cells were detected in the peripheral blood (day 7–10 after implantation). The treatment cycle was repeated, with a three-week treatment-free period, until euthanasia at the humane endpoint. Peripheral blood lymphocytes (PBLs) were collected weekly. PDX12 *n *= 14, PDX18 *n *= 7, PDX11/PDX18bis *n *= 8, PDX6bis/PDX13 *n *= 6.

To study the mechanism of action of azacitidine, peripheral blood was collected on days 15–20. Mice were euthanized 24 h after the last dose of the one cycle of treatment. *n *= 6 for all PDXs.

### DNA methylation analysis

DNA from the PDXs and the corresponding patient tumor samples was bisulfite-converted for DNA methylation analysis using an EZ DNA Methylation kit (Zymo Research, USA) and Infinium® MethylationEPIC (EPIC) BeadChips (Illumina Inc., USA) following the manufacturer’s guidelines. Data were analyzed as detailed in Supplementary Materials and Methods.

### Transcriptomic and functional analysis

RNA obtained from PDX cells was used for general transcriptomics and human endogenous retrovirus (HERV) and antiviral response gene expression analysis, which were quantified using a custom pipeline, as previously described [20] (see Supplementary Materials and Methods).

### Statistical analysis

Statistical analysis was performed using Prism software v10.0 packages (GraphPad Software, La Jolla, CA, USA). Student’s t-test or one-way ANOVA were used and statistically significant changes were considered for *p* values < 0.05. The data represent at least three reproduced experiments. Log-rank tests were used to determine significant differences between the mouse survival curves.

## Results

### TFHL PDX models recapitulate TFHL hallmarks

**Fig. 1:**
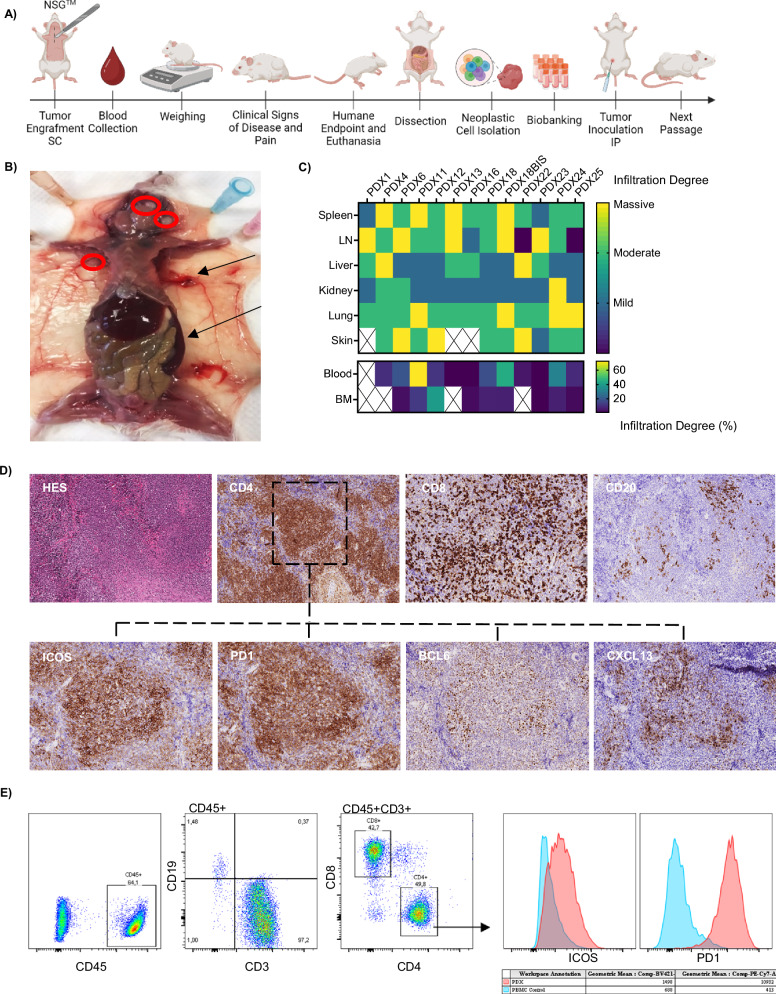
PDX generation and characterization. **A** Schematic representation of PDX generation. **B** Tumor involvement: Circles represent enlarged lymph nodes, and arrows indicate areas of high vascularization and splenomegaly. **C** Heatmap showing the extent of organ infiltration in different models. Tissue samples from the spleen, lymph nodes, liver, kidneys, lungs, and skin were semi-quantitatively evaluated by pathologists, and the degree of infiltration is categorized as mild, moderate, or massive. Blood and bone marrow infiltration are reported as the percentage of hCD45 + CD4 + PD1+ cells as measured by flow cytometry. **D** Phenotypic characterization of PDX models by immunohistochemistry. Staining of spleen tissue at passage 1. H&E (10x), TFH markers: *CD4* (10x), ICOS (20x), PD1 (20x), BCL6 (20x), and CXCL13 (20x), and microenvironment markers: CD8 (10x) and CD20 (10x). **E** Phenotypic characterization of PDX models by flow cytometry. Staining of splenocytes from passage 1. Panel includes CD45, CD19, CD3, CD8, CD4, ICOS, and PD1. LN lymph nodes, BM bone marrow.

We generated PDX models by engrafting patient TFHL lymph-node biopsies or circulating cells into NSG mice (Table 1). After subcutaneous or intraperitoneal implantation, the mice were clinically monitored (for tumor development, pain, and weight loss) and the percentage of circulating human tumor cells was evaluated weekly by flow cytometry until reaching the humane endpoint (Fig. 1A). At euthanasia, mice exhibited generalized polyadenopathy, splenomegaly, hepatomegaly, skin lesions, and abnormal vascularization of the skin (Fig. 1B).

**Fig. 2:**
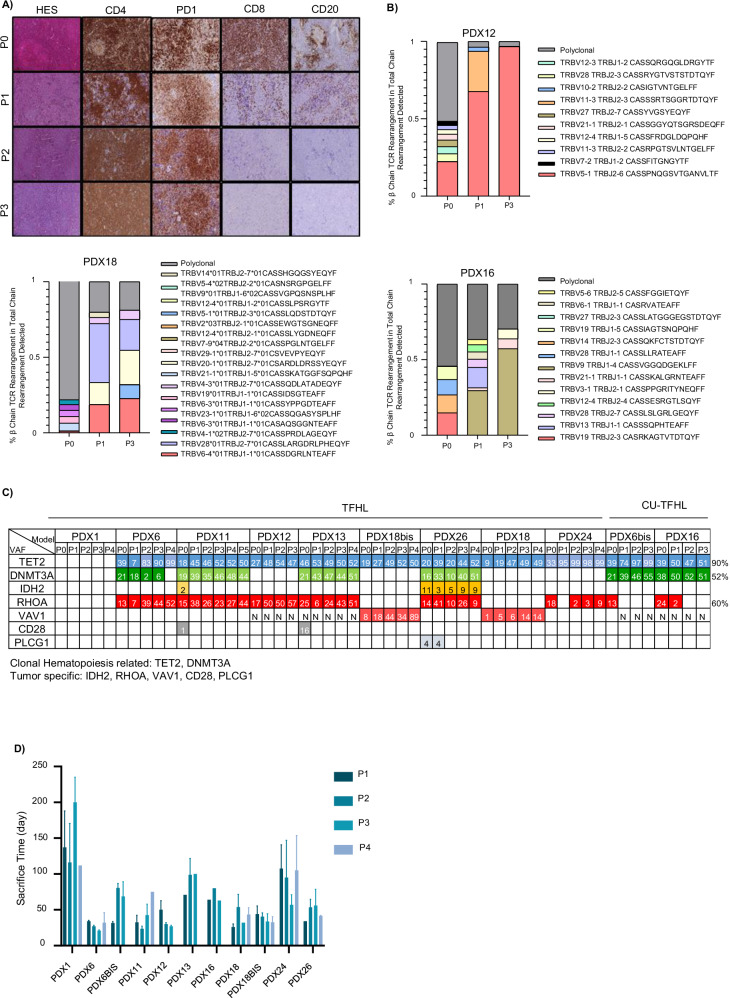
Molecular, pathological, and survival evolution during passaging. **A** Evolution of tumor cells and the microenvironment during passaging. IHC Staining of spleen tissue by HES (10X), tumor cell markers: CD4 (10X) and PD1 (20X), and microenvironment markers CD8 (10X) and CD20 (10X). **B** Clonal evolution of PDX models. The presence of T-cell clone on spleen cells was characterized by beta chain gene rearrangement and the percentage of β chain TCR rearrangement in total chain rearrangement was presented for PDX12, PDX18, and PDX16. **C** Mutational landscape of TFHL-PDX. The figure shows the distribution and VAF(%) of mutations in *TET2, DNMT3A, IDH2, RHOA, VAV1,* CD28, and *PLCG1*. Samples represented in dark blue bore at least two *TET2* mutations, whereas only one *TET2* mutation was detected in samples represented in light blue. DNMT3A R882* variants are represented in dark green, whereas samples represented in light green harbored DNMT3A mutations altering other residues. The models were sequenced using different versions of the lymphoma panel: PDX1: V.1.3, PDX6: V.1.3, PDX6Bis: V.1.2, PDX11: V.1.3, PDX12:V.1.2, PDX13:V.1.1-2, PDX16: V.1.2, PDX18: V.1.3, PDX18BIS: V.1.3, and PDX24: V.1.3. N: not available White cells: WT sequence. Representative example of the time to generate the PDX model, demonstrating the time (day) between tumor engraftment until euthanasia of the mouse. P0 Patient, P1 Passage 1, P2 Passage 2, P3 Passage 3, P4 Passage 4.

On histopathology, the mice showed tumor infiltration by small- to medium-sized atypical lymphoid cells in the spleen, liver, lymph nodes, kidneys, and skin. There was almost consistent spleen involvement, which predominated within the white pulp, whereas the red pulp only occasionally contained aggregates or sheets of neoplastic cells (Figs. 1C, D and 2A). Liver infiltration was variable but predominated in the portal areas. Lung infiltration was usually massive ( > 50% of the tissue involved), predominating along the bronchial trees. Lymph nodes were diffusively infiltrated, whereas kidneys generally showed mild interstitial infiltration. Skin lesions, generally located on the back or abdomen and often associated with alopecia, consisted of tumor infiltration in the epidermis, dermis, hair follicles, and subcutaneous fat. Tumor involvement in the bone marrow was limited, representing 1–40% of cellularity (Figs. 1C and S1A, B, C). Circulating cells were consistently detected in the blood by flow cytometry (Fig. S1C).

By IHC, most atypical cells were CD4^+^ICOS^+^PD1^+^BCL6^+^ admixed with generally scattered CD20^+^ B cells, sometimes EBER-positive, and CD8^+^ T-cells (Figs. 1D and  S2A).

FACS analysis of splenocytes showed a similar phenotype, with mostly CD4^+^ T-cells with a CD45^+^CD3^+^CD4^+^PD1^+^ICOS^+^TFH profile, representing a median of 51.62% splenocytes (IQR 12.21–89.34). B cells and CD8^+^ T-cells were also detected at first passage (Figs. 1E and  S2B).

Overall, we observed the development of patient-derived TFHL in the mice, enriched in neoplastic cells relative to the primary human samples but phenotypically recapitulating human TFHL.

### Evolution of malignant and reactive compartments during passages

We monitored the evolution of the human TME over successive passages. IHC showed a progressive decrease in B-cell and CD8^+^ T-cell populations, which became almost undetectable by P3 (Fig. 2A). Similarly, FACS analysis showed a decline in the median percentage of B cells (47,8%, 4,5%, and 1%) and CD8^+^ T-cells (50%, 20%, and 1%) at P1, P2, and P3, respectively (Fig. S2B, C), indicating a gradual loss of the TME, accompanied by an enrichment of malignant T-cells during passage (Fig. 2A).

Focusing on T-cells, we employed hybrid capture-based TCR sequencing assay and target gene sequencing (TGS) to investigate T-cell composition and T-cell clonal trajectories across different passages and organs. We observed distinct patterns.

In certain models (PDX1, PDX12, PDX13, and PDX26), the initial neoplastic T-cell clone, identified in the primary tumor, predominated. These malignant T-cells expanded progressively over passages, constituting the majority of T-cells at the latest passage, as illustrated in PDX12 (Fig. 2B). This finding aligns with TGS results, which showed a high variant allele frequency (VAF) for *RHOA* or *VAV1*, two mutations specific to TFHL neoplastic cells (Fig. 2C). The VAF reaching approximately 50% suggests, assuming the absence of loss of heterogeneity, that nearly all human cells belonged to the neoplastic clone (Fig. 2C, Fig. S3A, and Supplementary Table).

In other models (PDX6, PDX11, PDX18, PDX18bis, and PDX24), we observed oligoclonal tumors, characterized by the coexistence of the initial neoplastic clone alongside one or more clonal T-cell populations that were not expanded in the primary tumor, as they were undetectable in initial tumor. This scenario is exemplified by PDX18 (Figs. 2B and  S3B). TGS analysis revealed a discrepancy between the VAFs of *RHOA* or *VAV1* and those of *TET2* or *DNMT3A*. The VAFs of *TET2* and *DNMT3A* reached 50% or 100% (in cases of loss of heterozygosity) after a few passages, suggesting that nearly all cells originated from a common mutated hematopoietic progenitor. In contrast, the VAFs of *RHOA* and *VAV1* remained lower (ranging from 3% to 44% at passage 3), mirroring the proportion of the identified neoplastic clone in TCR sequencing (Supplementary Table). This suggests that the initial neoplastic clone coexists with other TCR-defined clones originating from a common *TET2* and/or *DNMT3A*-mutated hematopoietic progenitor.

In PDX6bis (derived from implantation of a second piece from the same biopsy as PDX6) (Fig. S3C) and PDX16 (Fig. 2B), we observed the expansion of a clonal T-cell population that was TCR-unrelated to the primary clone and did not harbor the oncogenic *RHOA* mutation. These findings suggest that PDX6bis and PDX16 likely developed a TCR clonally unrelated lymphoma (CU-TFHL) originating from a common *TET2/DNMT3A*-mutated precursor cell, with divergent evolution occurring before TCR rearrangement.

Finally, we investigated whether differential expansion of clonal T-cell populations could be influenced by variations in the microenvironment. We compared clonal composition in lung, spleen, and skin infiltrates across three models (PDX12, PDX18bis, and PDX26). Except for a distinct pattern observed in the skin of PDX18bis, no significant differences were found (Fig. S3D).

Although the evolution of neoplastic T-cell-specific mutations varied depending on the model, expansion of *TET2*-mutated cells was found in every tumor bearing a *TET2* mutation (TFHL and CU-TFHL). Conversely, engraftment of subclones bearing *CD28* and/or *IDH2* mutations was not detected in PDX11 and/or PDX13, whereas these mutations were present in the primary tumor (Fig. 2C). In addition, an *IDH2*-mutated subclone was engrafted into PDX26 mice and maintained across passages (Figs. 2C and  S4).

In total, we subcutaneously implanted 15 TFHL tumors and generated nine TFHL-PDXs for at least two passages, but mostly for 3–4 passages (median number of passages: 3 (1–5)). Two TFHL-implanted PDXs developed a clonally unrelated lymphoma with a TFH phenotype. In addition, one TFHL-implanted PDX model developed an EBV-infected B-cell lymphoma (Fig. S5). We also implanted the PBMCs of four patients via intravenous injection and generated one TFHL-PDX model. The success rates of subcutaneous and intravenous implantation were 10/15 (66%) and 1/4 (25%), respectively. The median time to the first detection of human CD45^+^ cells was 11.6 days (IQR: 7–20) and to that to euthanasia 63 days (IQR: 28–140) (Fig. 2D). The passage success rates were 79%, 92%, 80%, and 90% for P1, P2, P3, and P4, respectively. Implantation of cryopreserved cells or biopsies resulted in a higher failure rate (16%) than fresh-material engraftment (5%).

The cells isolated from different models were cryopreserved and a biobank of PTCL-PDX models was generated, allowing the models to be used for further analysis.

### TFHL PDXs recapitulate patient sensitivity to azacitidine

We selected six TFHL-PDXs (PDX6bis, PDX11, PDX12, PDX13, PDX18, and PDX18bis) derived from patients treated with azacitidine with available clinical data.

**Fig. 3:**
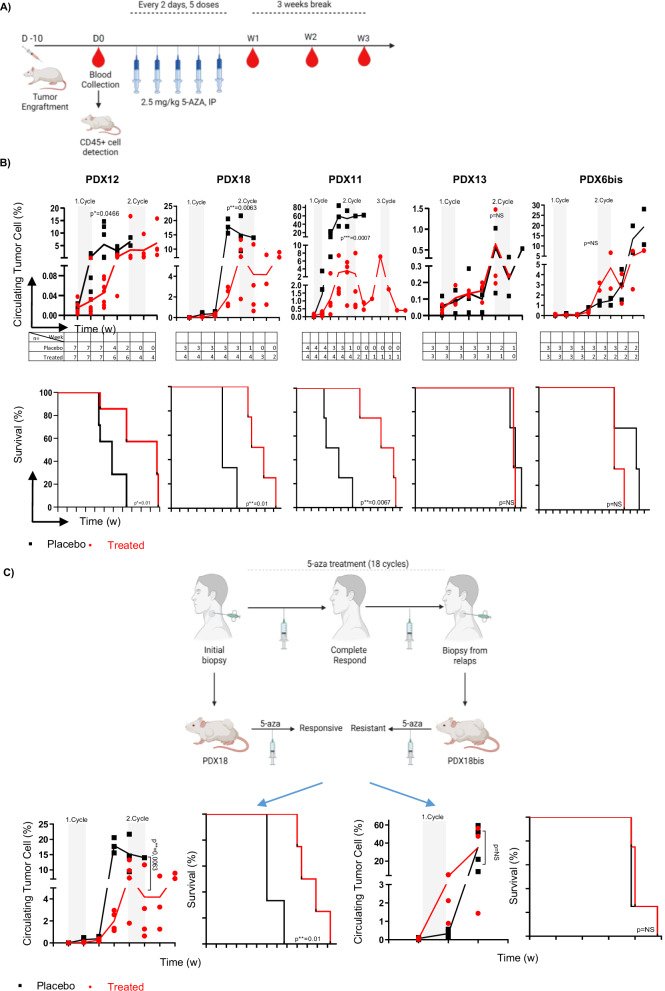
Sensitivity to azacitidine for TFHL-PDX models and their source patients. **A** chematic representation of azacitidine treatment. Tumor cells were implanted into NSG mice and circulating tumor cells were detected after 10 days. The treatment started upon detection of circulating tumor cells and the mice received intraperitoneal injections of 2.5 mg/kg azacitidine every other day for a total of five doses and the cycle was repeated every four weeks until the humane end point. **B** The efficacy of 5-azacitidine treatment in different PDX models. Three PDXs (PDX11, PDX12, and PDX18) developed from responsive models and two PDXs (PDX13 and PDX6bis) developed from refractory patients received azacitidine treatment. The weekly percentage of circulating tumor cells, followed during the treatment period, is shown on the first line of the figure. In the second line, the overall survival rate of azacitidine- and placebo-treated mice is shown. T-tests and Log-rank tests were used to determine significance changes in the percentage of circulating tumor cells and mouse survival curves, respectively. PDX12 *n *= 14, PDX18 *n *= 7, PDX11 *n *= 8, PDX6bis and PDX13 *n *= 6. **C** Schematic representation of the efficacy of azacitidine treatment in PDXs generated from the initial biopsy (PDX18 *n *= 7) or relapse biopsy (PDX18bis *n *= 8) of the same patient. Black dots represent placebo-treated mice and red dots azacitidine-treated mice.

After detection of circulating neoplastic cells by flow cytometry (days 7–20), mice were administered five doses of azacitidine 2.5 mg/kg or placebo every other day and the treatment was repeated every 28 days until the mice reached the humane endpoint (Fig. 3A). PDX12 and PDX18 were generated from patients who achieved a complete response after azacitidine treatment. Relative to those in the placebo group, the percentage of circulating hCD45^+^PD1^+^ cells in the blood of the treated group in PDX12 and PDX18 was significantly lower after two cycles of azacitidine treatment. The same was true after three cycles of treatment in PDX11, generated from a patient who had stable disease during azacitidine treatment. Notably, a reduced number of circulating cells was clearly detectable during the week of treatment. PDX12, PDX18, and PDX11 showed significantly longer overall survival (Fig. 3B).

By contrast, PDX13 and PDX6bis, generated from refractory patients, did not demonstrate a significant response in terms of either the percentage of circulating tumor cells or the overall survival of the mice (Fig. 3B).

In the responsive models, we also observed less tumor cell infiltration in various organs of azacitidine-treated mice than in placebo-treated mice (Fig. S6A). The spleen/body weight ratio was also significantly lower for azacitidine-treated mice than for placebo-treated mice (Fig. S6B/C). In addition, azacitidine-treated cells from the three responsive models showed lower ICOS expression (Fig. S6D). Of note, PD1 expression also decreased on PDX11 and PDX12 cells, but not on PDX18 cells or cells from resistant models (data not shown).

Interestingly, two TFHL-PDXs (PDX18 and PDX18bis) were generated from a single patient at relapse. PDX18 was obtained before the patient received azacitidine, resulting in a durable response for 18 cycles before experiencing progression, whereas PDX18bis was obtained from a new biopsy after progression. Although PDX18 was sensitive to azacitidine, PDX18bis was resistant, recapitulating the sensitivity of the patient (Fig. 3C).

### Azacitidine induces DNA hypomethylation in sensitive models

**Fig. 4:**
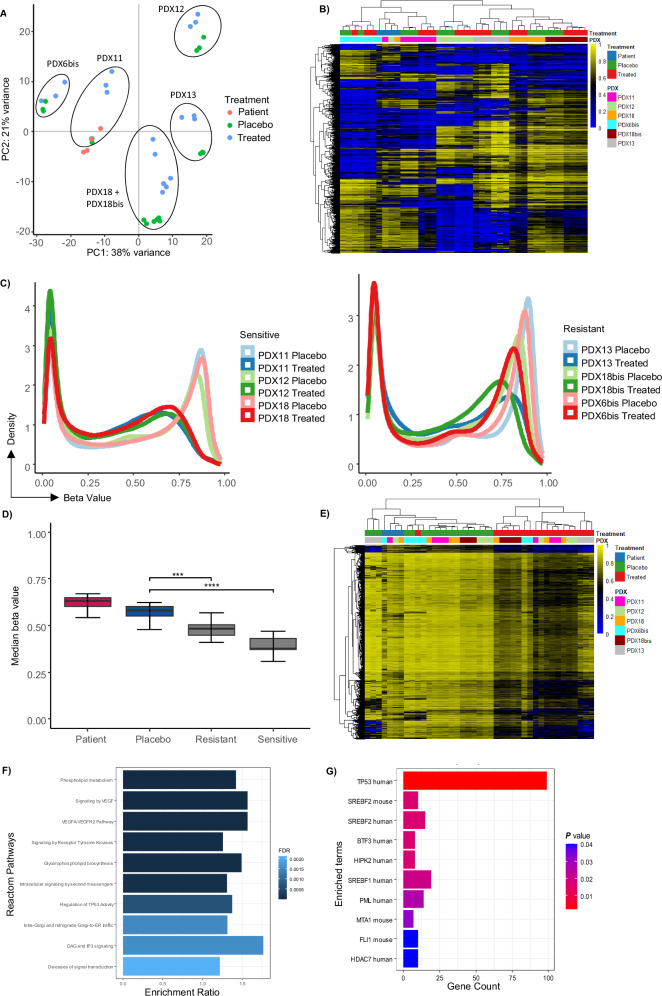
Changes in DNA methylation induced by azacitidine treatment. **A** Principal component analysis with the 10,000 most variable CpGs. Colors indicate the treatment status or primary tumor sample. Circles highlight the six different TFHL-PDX models. **B** Heatmap showing the beta values for the 10,000 most variable CpGs. Hierarchical clustering shows separation between the six different TFHL-PDX models. **C** Density plot showing mean DNA methylation for the sensitive (PDX11, PDX12, and PDX18) and the resistant (PDX6bis, PDX13 and PDX18bis) models. **D** Median global DNA methylation for the patient tissue group, as well as the placebo- and azacitidine-treated TFHL-PDX models. T test, ***p < 0.001, **** *p *< 0.0001. **E** Heatmap showing the beta values for differentially methylated CpGs of sensitive models. Treated vs Placebo (PDX11, 12, 18) FDR < 0.0000001 ΔBeta_mean_ = 0.2 *n *= 34,835 CpGs. **F** Enrichment analysis of genes associated with differentially methylated CpGs by Webgestalt: Reactome. **G** Enrichment analysis of genes associated with differentially methylated CpGs for transcription factors by EnrichR: TRRUST Transcription factors 2019.

To better understand the effect and mechanism of action of azacitidine in TFHL, we treated six models (PDX6bis, PDX11, PDX12, PDX13, PDX18 and PDX18bis) with azacitidine, three of which (PDX11, PDX12, and PDX18) were sensitive to azacitidine, whereas PDX6bis, PDX13 and PDX18bis were resistant. We modified the experimental design to allow for sufficient exposure to the drug to evaluate a biological effect, as well as to collect material for subsequent analyses. DNA methylation analyses were performed using Illumina Infinium Methylation BeadChips. The results were visualized using a two-dimensional PC projection. The PDX samples clustered predominately by model and were distinct from the primary tumors, although the distribution in PC1 and PC2 was maintained (Fig. 4A). Among the sensitive models, the azacitidine-treated and untreated samples clustered distinctly on separate branches, whereas the difference was less pronounced in the resistant models (Fig. 4B). The difference between the treated and untreated models was mainly related to a shift toward lower DNA methylation levels (Fig. 4C). Although TFHL-PDX generally showed lower global DNA methylation than the patient samples, there was a significant decrease in global DNA methylation after azacitidine treatment (Fig. 4D). Searching for commonly hypomethylated loci within the sensitive and resistant models, we identified 34,835 differentially methylated CpGs in the sensitive models and none in the resistant models (Fig. 4E). The analysis of genes linked to those differentially methylated CpGs using the Reactome database revealed an enrichment in signaling, and metabolism, but also in the regulation of TP53 activity (Fig. 4F), TP53 was identified as the most enriched transcription factor associated with these genes (Fig. 4G). Overall, TFHL-PDX maintained the primary lymphoma-associated DNA methylation profile after treatment, and azacitidine administration was associated with a global loss of DNA methylation that was seemingly more pronounced in azacitidine-sensitive than resistant models, and resulted in DNA methylation changes across various regions interacting with the TP53 and belonging to various pathways.

### Azacitidine induces p53-mediated apoptosis

**Fig. 5:**
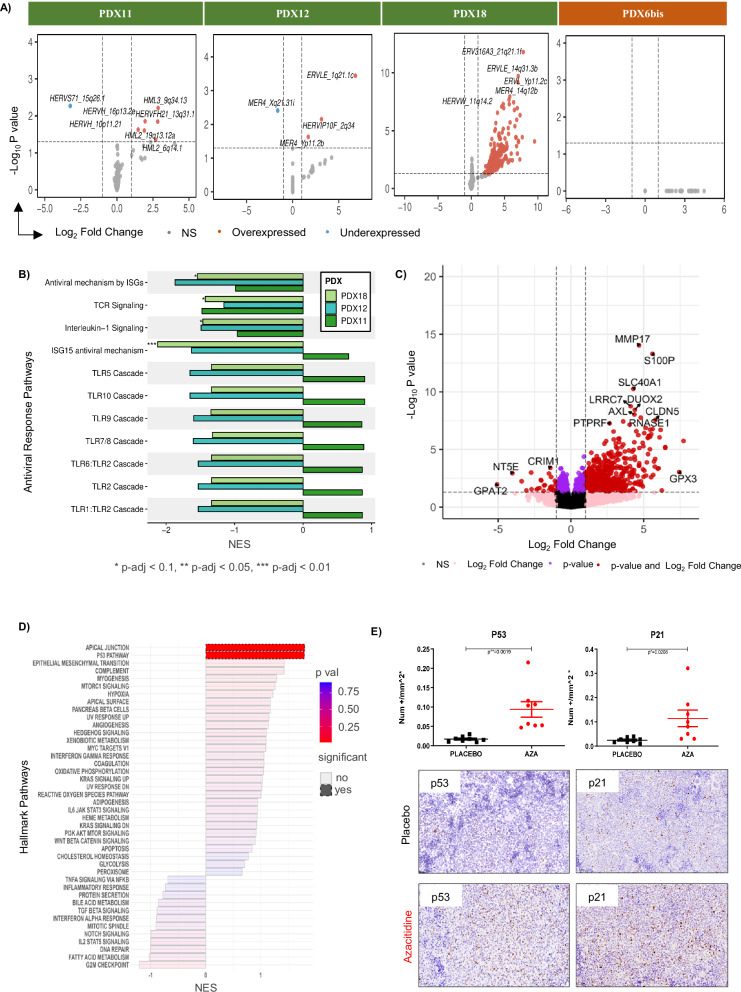
Effect of azacitidine on HERV and gene expression profiles. **A** Volcano plots of differentially expressed HERV genes in PDX11, PDX12, PDX18, and PDX6bis for treated versus placebo mice. **B** Selected antiviral response gene set enrichment of responsive models (*p-adj < 0.1, **p-adj < 0.05, ***p-adj < 0.01). NES: normalized enrichment score. **C** Profile of changes in expression of protein-coding genes after azacitidine treatment in sensitive models (PDX11, PDX12, and PDX18). **D** Hallmark gene set enrichment of DEGs (i.e. genes with |log2(foldchange)| > 1 and *p*-value < 0.05) in sensitive models (PDX11, PDX12, and PDX18). The X-axis shows normalized gene enrichment, and the Y-axis shows hallmarks. **E** Effect of azacitidine on the p53 pathway at the protein level by IHC. Positive cells were quantified using QuPath software and normalized to the CD4^+^ cell count and the placebo.

We further evaluated the effect of azacitidine treatment on sensitive models (PDX11, PDX12, and PDX18) and a resistant model (PDX6bis) at the transcriptomic level by RNA sequencing. The analysis first focused on human endogenous retroviruses (HERVs). Differential gene expression analysis showed slight changes in HERV expression for PDX11 and PDX12 after azacitidine treatment, whereas overexpression of HERV genes was evident for PDX18. There was no significant change in HERV transcription upon treatment in the TFHL-PDX6bis azacitidine-resistant model (Fig. 5A). Gene set enrichment analysis (GSEA) of antiviral response genes showed significant downregulation in PDX18 but not PDX11 or PDX12. Despite significant changes in HERV expression in responsive models, no significant enrichment in antiviral response genes was identified (Fig. 5B) and no significant transcriptomic change was observed for viral mimicry-associated genes after azacitidine treatment (Fig. S7A).

Finally, we analyzed the effects of azacitidine treatment on the global transcriptome. We observed global gene overexpression, with 488, 309, and 784 upregulated genes versus 46, 11, and 107 downregulated genes in the sensitive PDX11, PDX12, and PDX18, respectively, whereas the resistant PDX6 showed 599 upregulated and 48 and downregulated genes (Fig. S7B). We performed functional analysis of differentially expressed genes (DEGs) for each model using hallmark gene sets. Genes upregulated by azacitidine treatment in PDX11 were significantly associated with p53 pathway and TNF-alpha signaling via the NF-κβ pathway, whereas changes in gene expression after azacitidine treatment in PDX18 were associated with the upregulation of genes involved in apoptosis and downregulation of those of the mitotic spindle, TNF-alpha signaling via the NF-κβ pathway, the G2M checkpoint, and E2F targets. No significant enrichment was observed for PDX12. The resistant PDX6bis showed significant enrichment in genes of the p53 pathway, as well as the downregulation of those of mitotic spindles and G2M checkpoints (Fig. S7C).

Although these analyses identified the deregulation of critical hallmarks of cancer in each model, we failed to identify a common pathway, most likely due to the heterogeneity of TFHL. Thus, data from the responsive models (7 treated mice vs. 9 placebo mice from PDX11, PDX12, and PDX18) were pooled: 671 genes were upregulated and six downregulated in azacitidine-treated samples versus untreated samples (Fig. 5C). In addition, GSEA showed two significantly upregulated pathways, p53 and apical junction signaling (Fig. 5D). Interestingly, by GSEA, we found 11 genes differentially expressed related to the p53 pathway in the three azacytidine-sensitive TFHL-PDXs. They included *PLK2, APP, CTSD, CEPBA, PHLDA3*, and *AK1* (Fig. S7D), with increased expression paralleling promotor hypomethylation (Fig. S8A).

These changes in apoptosis and proliferation of azacitidine-exposed cells were validated at the protein level by IHC of tumor samples (spleen) of TFHL-PDX, which showed a significant increase in p53 and p21 expression in azacitidine-treated samples (Fig. 5E), as well as that of cleaved-caspase 3 as an indicator of apoptosis (Fig. S8B), whereas the expression of BCL2 and MIB1/Ki67 was downregulated, indicating lower proliferation in azacitidine-treated mice (Fig. S8C).

## Discussion

Although significant progress has been made in understanding the oncogenesis of TFHL in recent years, including the mutational landscape, high frequency of clonal hematopoiesis, and role of the TME in supporting the initiation and maintenance of TFHL, therapeutic progress has remained limited, leading to only limited improvement in the survival of TFHL patients. Therapeutic progress is hindered by the lack of preclinical models, particularly TFH-derived cell lines. Notably, no permanent cell line has ever been established, indicating that TFHL cell survival likely relies on interactions with the TME. Recent data on murine models suggest that B cells play a critical role in supporting TFHL development [21–23], whereas the role of stromal cells is still unclear. Although the engraftment rate for most cancers ranges from 10% to 60% [24], we observed a high success rate of engraftment, with 11 of 21 (55%) TFHL, suggesting that the murine microenvironment might contribute to support the growth and survival of neoplastic TFH cells. Subcutaneous implantation of tumor pieces, comprising neoplastic, reactive hematopoietic, and stromal cells, resulted in a higher success rate than intravenous inoculation lacking the stromal microenvironment. This suggests that the human TME may aid successful initiation of PDXs, although it appears to not be critical. In our model, neoplastic TFH cells continued to expand, whereas B cells disappeared after 3–4 passages, indicating that neoplastic TFH cells become independent of B cells but likely require signals from the mouse microenvironment, likely provided by mouse stromal or myeloid cells, as NSG mice lack T, B, and NK cells. However, no strong data have been generated so far to support this hypothesis and further studies are needed to identify the murine elements and mechanisms that support TFHL growth, as they could be exploited therapeutically.

A notable observation of this study was the emergence of oligoclonal TFHL, characterized by the coexistence of the neoplastic clone and other TCR-unrelated clones, all bearing the same primary *TET2* mutations, mimicking the findings of a previous study on oligoclonal TFHL [25]. Furthermore, we observed lymphoproliferative disease (LPD), referred to as TCR clonally unrelated TFHL (CU-TFHL), in certain PDX models. This form is aggressive, involves multiple organs, impacts mouse survival, and is transplantable, exhibiting hallmarks of cancer.

The independent clones observed in both oligoclonal TFHL and CU-TFHL were not detected in the primary samples -within the sensitivity limits of our assay- suggesting they were not pre-expanded clones. These clones were characterized by *TET2* ± *DNMT3A* mutations and a TFH phenotype, suggesting a link between *TET2* mutations, TFH differentiation, and cellular transformation. However, it is well established that *TET2* mutation alone is insufficient to drive tumorigenesis. Therefore, identifying additional factors, such as secondary mutations in T-cells or crosstalk with the murine microenvironment, would be essential to better understand the oncogenic mechanisms underlying these lymphoproliferative disorders.

The major application of PDX is to test new drugs or develop co-clinical trials. The relevance of our TFHL-PDX may be debatable due to the progressive loss of the human TME during passage. However, we demonstrate that they recapitulate the patient’s sensitivity to azacitidine. The generation of TFHL-PDX18 and TFHL-PDX18bis from a single patient, with one PDX being sensitive and the other being resistant to azacitidine, may be particularly useful for studying the mechanism of azacitidine resistance and how to overcome it.

By analyzing changes in DNA methylation and the expression of antiviral mimicry and hallmark genes in azacitidine-treated mice, we observed global DNA hypomethylation, particularly in sensitive TFHL cells, and the deregulation of gene expression, particularly of those involving cancer-related pathways. Remarkably, a common effect consisted of the activation of p53-related apoptosis, similar to the cytotoxic effect of chemotherapy [26], although it is unclear whether this activation is a direct effect of the azacitidine or a consequence of cell cycle progression and DNA damage. Despite the upregulation of HERV gene transcripts, we did not detect the induction of viral mimicry-related gene expression. The lack of an increased antiviral response in TFHL-PDX could be because NSG mice possibly possess strong basal antiviral immunity.

Overall, we show here that TFHL-PDX models are suitable for testing new drugs and studying disease biology. We improve current understanding of the effects of azacitidine, which induces DNA hypomethylation and associated with for p53-mediated apoptosis of neoplastic cells.

## Supplementary information

Supplementary Material and Method and Figures

Supplementary Table

## Data Availability

The data generated in this study are freely available to any researcher wishing to use them upon request from the corresponding author. RNA sequencing data is available in the NCBI Sequence Read Archive (SRA) repository, Bioproject PRJNA1242739.
