# Supplementary material for: Induction of p53-mediated apoptosis by azacitidine in patient-derived xenograft follicular helper T-cell lymphoma model
Source: Leukemia. 2025 May 20;39(7):1744–55. doi: 10.1038/s41375-025-02628-0 (PMC12208902; doi:10.1038/s41375-025-02628-0)
Supplement: Supplementary file 1 — Supplementary Material and Method and Figures [file 41375_2025_2628_MOESM1_ESM.pdf]

Animal studies

The sample size was chosen with resource equation method. The mice were randomized into different groups by using GraphPad software. No investigator blinding was done.

Mouse Dissection and Tumor Cell Isolation

The disease development in the mice was followed by circulating tumor cells, body weight loss and clinical signs of disease and pain. Circulating tumor cells were weekly detected on flow cytometry on the peripheral blood. The weekly body weight change was measured. Clinical signs of disease and pain were observed daily. The mice were sacrificed at humane endpoint.

The mouse was dissected and the internal organs were gently collected. Spleen size and weight were measured. Pieces of each organ were transferred to formaldehyde for histopathological analysis. The spleen was mechanically dissociated to collect the tumor cells. Liver, kidney and lung tissues were enzymatically and mechanically dissociated. The organs were treated with PBS containing 500 mg/lt Collagenase IV (Sigma Aldrich, USA), 50 µg/lt DNAse I (Thermo Fisher Scientific, USA), 2% Fetal Bovine Serum (FBS)(Thermo Fisher Scientific, USA), and 0,6% Bovine Serum Albumin (BSA) (Thermo Fisher Scientific, USA) at 37° and the cell suspension was collected after mechanical dissection. The skin from cutaneous lesions of the mice was recovered after depilation and the subdermal fat was removed. The skin tissue was treated with RPMI containing 250 mg/lt Liberase™ (Sigma Aldrich, USA) and DNAse I addition (20 mg/lt, Sigma Aldrich, USA) at 37° C.

Isolated tumor cells and tissues were cryopreserved into freezing medium including 10% DMSO, 50% FBS and 40% RPMI and stored in -150°C.

Immunohistochemisty

Organs were fixed with buffered 4% formaldehyde, pH 7, and embedded in paraffin. Immunohistochemical (IHC) studies on the formalin-fixed paraffin-embedded (FFPE) tissue sections were conducted using an automated immunostainer (BON-III Autostainer, Leica Microsystems, UK) following the manufacturer's protocols with antibodies directed against CD20, CD30, CD3, CD4, CD8, ICOS, PD1, BCL6, CXCL13, IDH2R172K, LMP, EBNA2, p53, p21, cleaved-Caspase3, and MIB1/Ki-67, whereas EBV was detected by in-situ hybridization with EBER probes (1).

The slides were scanned using a Nanozoomer 360 (Hamamatsu, Japan) and examined using CaseViewer software. Qupath software was used to quantiyfy staining.

	Antibody	*Clone*	*Dilution*	*Manufacturer*
B cell	*CD20*	L26	1 :500	Dako France Sas
	CD30	Ber-H2	1 :70	Dako France Sas
T cell	*CD3*	F7.2.38	1 :50	Dako France Sas
	CD8	4B12	1 :100	Leica Biosystems
TFH cell	*CD4*	C8/144B	1 :200	Dako France Sas
	ICOS	SP98	1 :100	Abcam
	PD1	NAT105	1 :200	Abcam
	BCL6	LN22	1 :50	Leica Biosystems
	CXCL13	53610	1 :50	R&D Systems
	IDH2R172K	R172K	1 :200	Eweast Biosciences
EBV	*EBNA2*	PE2	1 :50	Abcam
	LMP	CS 1-4	1 :400	Dako France Sas
Apoptosis Proliferation	*p21*	EA10	1 :50	Calbiochem
	p53	D70	1 :20	Dako France Sas
	Cleaved-Caspase 3	Polyclonal	1 :100	Sigma Aldrich
	MIB1	30-9	Prediluted 1 :1000	Roche

<u>In Situ Hybridization</u>

Deparaffinized tissue sections were stained with a fluorescein isothiocyanate-coupled specific peptidic nucleic acid probe to detect Epstein-Barr encoded RNA transcripts 1 and 2 (EBERs). The tissue sections were heat denatured, digested with proteinase K and incubated overnight. Antifluorescein isothiocyanate monoclonal mouse antibodies, rabbit antimouse antibodies followed by alkaline phosphatase and antialkaline phosphatase monoclonal mouse antibodies were used for the detection of heterodimers. 5-bromo-4-chloro-3-indolyl phosphate and nitroblue tetrazolium were used for the observation of the staining, as previously described (2),(1).

<u>Flow Cytometry</u>

Isotype controls, compensation beads (Biolegend) and rainbow calibration particles (Biolegend) were used for the optimal analyses.

Antibody	Clone	Fluorochrome	Isotype	Manufacturer
CD45	HI30	FITC	Mouse IgG1, κ	Biolegend
CD19	HIB19	APC.Cy7	Mouse IgG1, κ	Biolegend
CD3	SK7	BV510	Mouse IgG1, κ	Biolegend
CD4	RPA-T4	BV605	Mouse IgG1, κ	Biolegend
CD8	RPA-T8	BV711	Mouse IgG1, κ	Biolegend
ICOS	C398.4A	BV421	Armenian Hamster IgG	Biolegend
PD1	A17188B	PE.Cy7	Mouse IgG2b, κ	Biolegend

Nucleic Acid Extraction

Genomic DNA (gDNA) or RNA was extracted from FFPE tissue or fresh cells using the Maxwell FFPE Plus DNA Kit (Promega, USA) or AllPrep DNA/RNA Mini/Micro Kits (Qiagen, CA) according to the manufacturers' recommendations, respectively. Nucleic acids were quantified via Qubit 3.0 using the Qubit dsDNA/RNA HS (Thermo Fisher, USA) assay kits.

Mutational Profiling by Next Generation Sequencing

Mutation analysis was performed on gDNA samples after amplification using 15 ng of tumoral gDNA as template and utilizing an Ampliseq custom panel (Thermo Fisher Scientific, USA). This includes a multiplex PCR-based library preparation method that targeted various coding regions (70–150 bp), involved in T cell lymphoma. Subsequently, the amplicons underwent digestion, barcoding, and amplification using the Iom Ampliseq Kit for Chef DL8 (Thermo Fisher Scientific, USA), following the provided instructions. Following DNA library preparation and quantification, each library, at a concentration of 25 pM, underwent multiplexing and clonal amplification on ion-sphere particles (ISP) through emulsion PCR performed on The Ion Gene Studio S5 (ion, Thermo Fisher, USA) supported by the Genomic Platform of Mondor Biomedical Research Institute. The ISP templates were loaded onto an Ion-510, 520 or 530 chip and subjected to sequencing on an S5 sequencer with the Ion 510TM & Ion 520TM & Ion 530TM Kit–Chef, following the manufacturer's instructions. The minimum sequencing depth of the samples was 1000x. Run performance was evaluated, and data analysis was conducted using the TORRENT SUITE Software versions between v.5.10 and v.5.16 (Thermo Fisher Scientific, USA). Sequencing data were processed through SeqPilot (JSI Medical Systems, Germany). Visualization of single-nucleotide variants (SNV) was accomplished using the Integrative Genomics Viewer, employing low stringency settings (threshold: 1%). The specific classes of variants detected included extronic (nonsynonymous SNV, stop gain, stop loss, frameshift deletion or insertion, non-frameshift deletion or insertion),

and intronic (only splicing, only +/- 1-2 bp before/after exon) variants. The overage of the panel was 3001,4x [616-6398], the minimum coverage of the target region was 500 and minimum VAF was determined as 1%.

NGS T cell lymphoma panel expanded through 3 different versions: V.1.1, V.1.2 and V.1.3. The first panel V.1.1 included the genes encoding CD28, DNMT3A, IDH2, PLCG1, RHOA, SETD2, STAT3, STAT5B and TET2. In the second version V.1.2, the JAK1 and JAK3 genes were added to the panel together with an improved coverage quality of genes of V.1.1. Finally, V.1.3 had an improved coverage quality on V1.2 genes and addition of the MSC, TNFRSF1B, TP53 and VAV1 genes.

NGS B cell lymphoma panel included the genes encoding ARID1A, B2M, BCL2, BRAF, BTK, CARD11, CCND3, CD58, CD79A, CD79B, CDKN2A, CDKN2B, CIITA, CREBBP, CXCR4, EP300, EZH2, FOXO1, GNA13, ID3, IRF4, MEF2B, MYC, MYD88, NOTCH1, NOTCH2, PIM1, PLCG2, PRDM1, SOCS1, STAT6, TCF3, TNFAIP3, TNFRSF14, TP53, and XPO1.

DNA Methylation Analysis

DNA from PDX and the corresponding patient tumor samples was bisulfite-converted for DNA methylation analysis using the EZ DNA Methylation kit (Zymo Research, USA) according to manufacturer's instructions. DNA methylation was interrogated by the Infinium® MethylationEPIC (EPIC) and Infinium Methylation v2.0 BeadChips (Illumina Inc., USA) following the manufacturer's guidelines. Raw intensity files (idat) were imported into the R programming (v4.3.1) using the minfi package. Data was normalized to controls using the preprocess Noob function without background correction. Beta values were calculated representing the percentage of DNA methylation at a certain cytosine base. For downstream analysis, loci on gonosomes were excluded. Based on variance between all samples, the 10,000 most variable CpGs were selected and displayed as unsupervised analysis. Sample PDX13 was excluded from the calculation of the 10,000 most variable CpGs due to high sample-specific variance and was only displayed in the unsupervised analyses. Principal component analysis (PCA) was performed using the prcomp function and heatmaps were generated with the pheatmap package. Differentially methylated CpGs were calculated using the limma package and adjusted for multiple testing using Benjamini-Hochberg. For enrichment analysis, genes associated with differentially methylated CpGs were used. Enrichmenr analysis was performed using the WebGestaltR package (pathway Reactome) and the enrichR package (TRRUST_Transcription_Factors_2019).

RNA-sequencing

RNA sequencing libraries were prepared with the Illumina Stranded mRNA Prep, Ligation (Illumina, USA) kit following manufacturer's recommendations. The protocol consisted of PolyA mRNA capture with oligo dT magnetic beads (25-1000 ng total RNA), purification and fragmentation of mRNA to approximately 300-400 pb, synthesis of first strand DNA (cDNA) using reverse transcriptase and random primers, replacement of dUTP to dTTP for the synthesis of second cDNA strand, addition of adenine and thymine bases to fragment ends, and ligation of IDT for Illumina TruSeq UD Indexes (Illumina, USA) adaptors for amplification of the library by PCR for sequencing. The library sequencing was performed by NextSeq 500 sequencer (Illumina, USA) with 21 millions reads in 75 bp paired-end mode following Mid Output Kit v2.5 1x150 cycles/phiX kit protocol.

Transcriptomic and Functional Analysis

RNA obtained from PDX cells was used for general transcriptomics, Human Endogeneous Retrovirus (HERV) and antiviral response gene expression analysis which were quantified using a custom pipeline, as previously described (3). Briefly, RNA-seq reads were aligned to the GRCh38.p14 reference genome using bowtie2 (v2.5.3) with custom parameters to retain multimaps (--no-unal --score-min L,0,1.6 -k 100 --very-sensitive-local). SAM outputs were converted to BAM files using SAMtools (v1.19). HERV was quantified using Telescope (4) (v1.0.3, options --reassign_mode exclude) and featureCounts (v2.0.6, options -Q 10 -s 2) respectively.

For HERVs, a custom transcriptome with 14 968 HERV transcriptional units compiled from RepeatMasker annotations was used as reference. For antiviral genes, the GENCODE v45 reference transcriptome was used. Raw counts were concatenated and normalized independently for each PDX using DESeq2 (v1.42.0) with variance stabilizing transformation (VST). Differential expression analysis was performed using DESeq2 (v1.42.0).

Differentially expressed human genes (DEG) were subjected to gene set enrichment analysis. DEG, functional gene set enrichment analyses were performed with clusterProfiler (v4.10.1) and fgsea (v1.24.0) (5). Immune gene sets obtained from the Reactome database and a custom viral mimicry gene set manually curated from relevant papers (6–9). For visualization of the

results through volcano plots and heatmaps, several packages as enhancedVolcano (1.20.0), ComplexHeatmap (v2.18.0) and pheatmap (v1.0.12) in R program were utilized.

References

1. Nicolae A, Bouilly J, Lara D, Fataccioli V, Lemonnier F, Drieux F, et al. Nodal cytotoxic peripheral T-cell lymphoma occurs frequently in the clinical setting of immunodysregulation and is associated with recurrent epigenetic alterations. Mod Pathol. 2022;35(8):1126-36.

2. Ortonne N, Dupuis J, Plonquet A, Martin N, Copie-Bergman C, Bagot M, et al. Characterization of CXCL13+ neoplastic t cells in cutaneous lesions of angioimmunoblastic T-cell lymphoma (AITL). Am J Surg Pathol. 2007;31(7):1068-76.

3. Alcazer V, Bonaventura P, Tonon L, Michel E, Mutez V, Fabres C, et al. HERVs characterize normal and leukemia stem cells and represent a source of shared epitopes for cancer immunotherapy. Am J Hematol. 2022;97(9):1200-14.

4. Bendall ML, De Mulder M, Iñiguez LP, Lecanda-Sánchez A, Pérez-Losada M, Ostrowski MA, et al. Telescope: Characterization of the retrotranscriptome by accurate estimation of transposable element expression. PLoS Comput Biol. 2019;15(9):e1006453.

5. Korotkevich G, Sukhov V, Budin N, Shpak B, Artyomov MN, Sergushichev A. Fast gene set enrichment analysis. biorxiv. 2016;060012.

6. Roulois D, Yau HL, Singhania R, Wang Y, Danesh A, Shen SY, et al. DNA-demethylating agents target colorectal cancer cells by inducing viral mimicry by endogenous transcripts. Cell. 2015;162(5):961-73.

7. Chiappinelli KB, Strissel PL, Desrichard A, Li H, Henke C, Akman B, et al. Inhibiting DNA methylation causes an interferon response in cancer via dsRNA including endogenous retroviruses. Cell. 2015;162(5):974-86.

8. Scheller M, Ludwig AK, Göllner S, Rohde C, Krämer S, Stäble S, et al. Hotspot DNMT3A mutations in clonal hematopoiesis and acute myeloid leukemia sensitize cells to azacytidine via viral mimicry response. Nat Cancer. 2021;2(5):527-44.

9. Cuellar TL, Herzner AM, Zhang X, Goyal Y, Watanabe C, Friedman BA, et al. Silencing of retrotransposons by SETDB1 inhibits the interferon response in acute myeloid leukemia. J Cell Biol. 2017;216(11):3535-49.

Supplementary Figure 1 – Pathophysiology of TFHL-PDX models

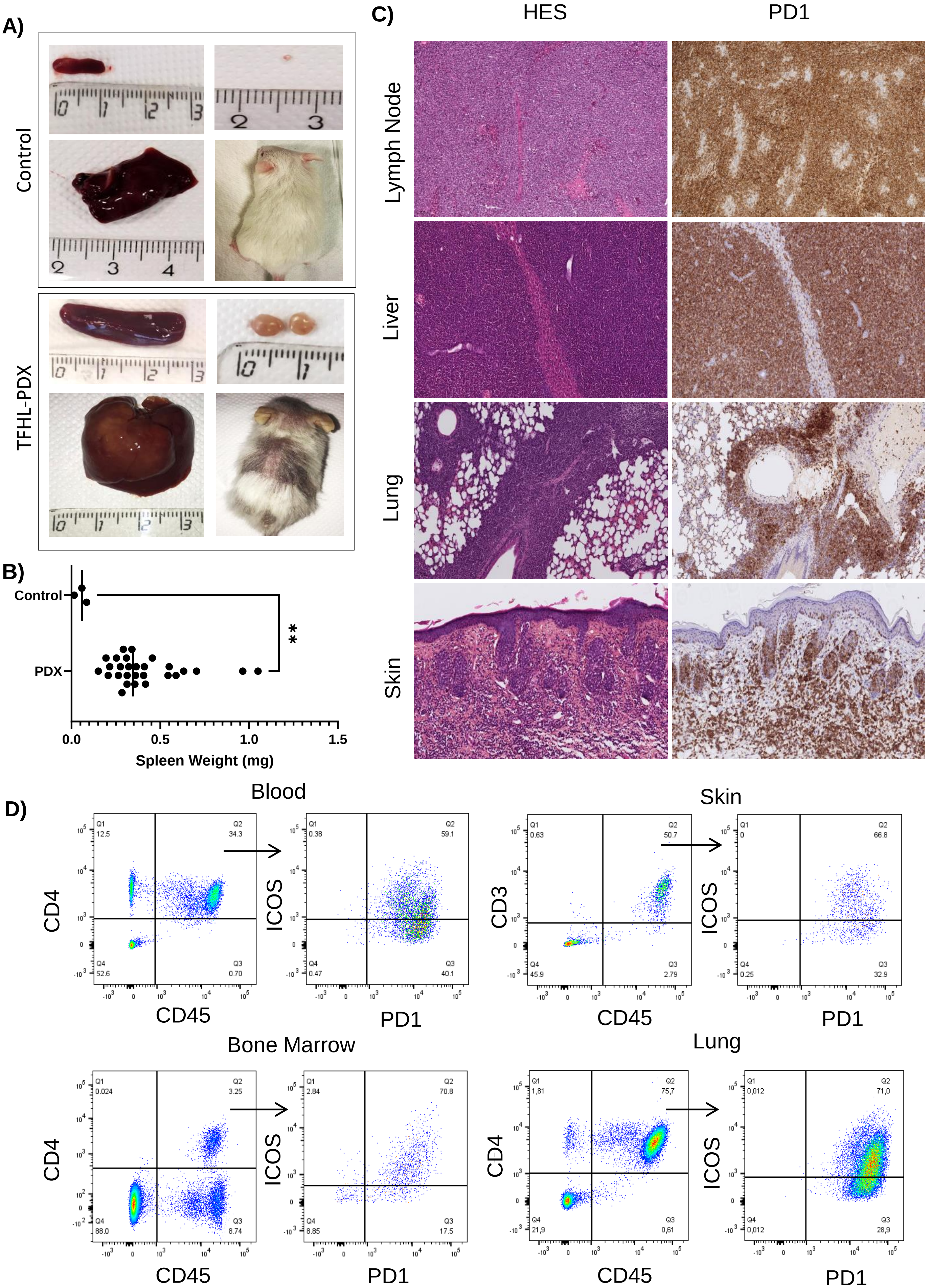

Supplementary Figure 1. Pathological findings of TFHL PDX models
A) Spleen, lymph node, liver and skin aspect in control and in TFHL PDX
B) Spleen weight of TFHL PDX compared to control mice
C) IHC phenotype of different organs lymhp nodule, liver lung and skin HES (10X), PD1 (10X)
D) FACS phenotype of different organs (blood, bone marrow, skin, and lung)

Supplementary Figure 2 – Tumor microenvironment

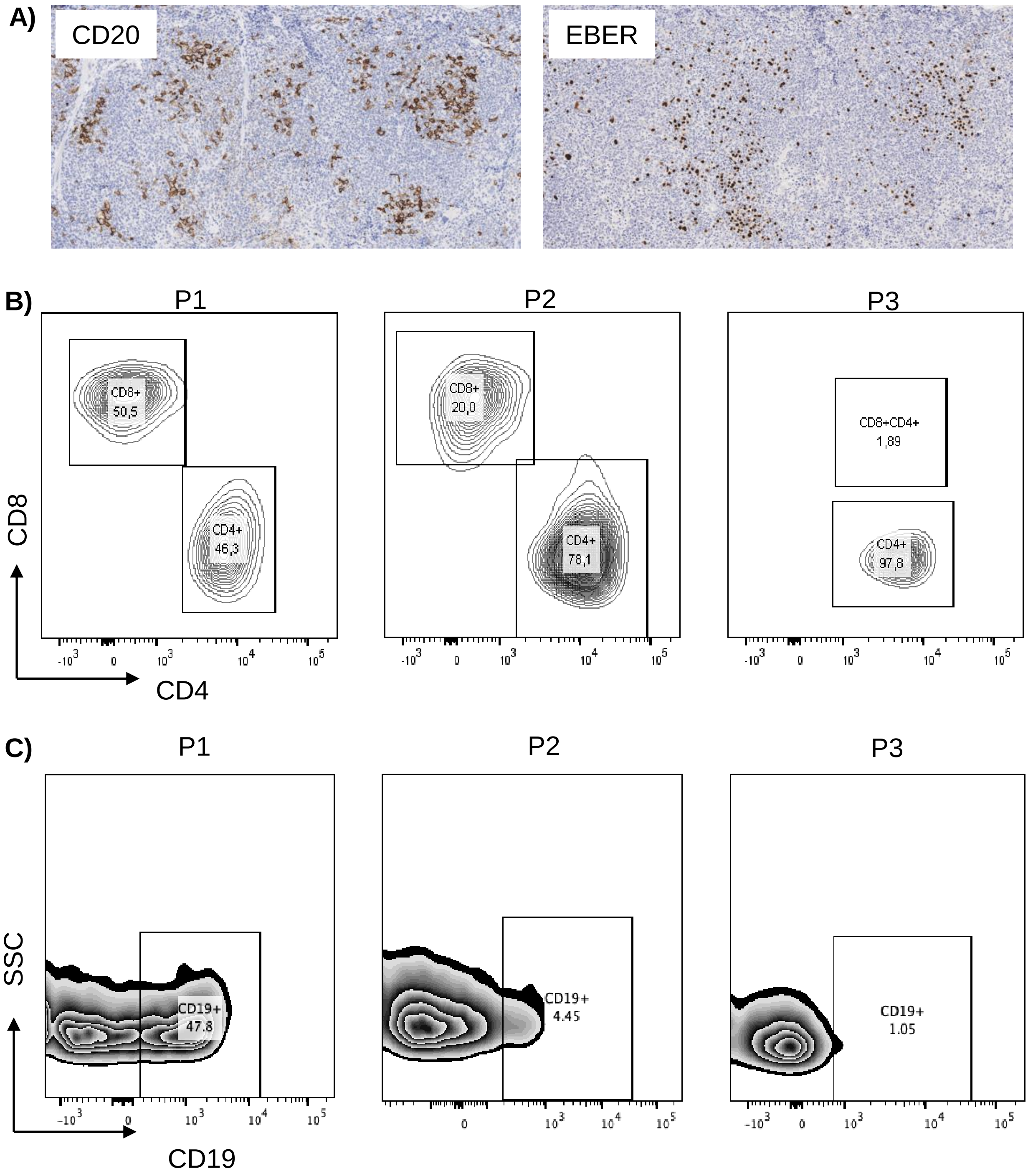

A) Phenotype of spleen for EBV infected B blast in tumor microenvironment. IHC; CD20 (10X), FISH EBER (10X)

B) FACS phenotype of splenocytes through passages showing decrease on CD8+ population. Gating on CD45+CD3+ population.

C) FACS phenotype of splenocytes through passages showing decrease on B lymphocytes. Gating on CD45+ population.

P1:Passage 1, P2: Passage 2, P3: Passage 3

Supplementary Figure 3 – TCR Clonality of PDX Models

A)

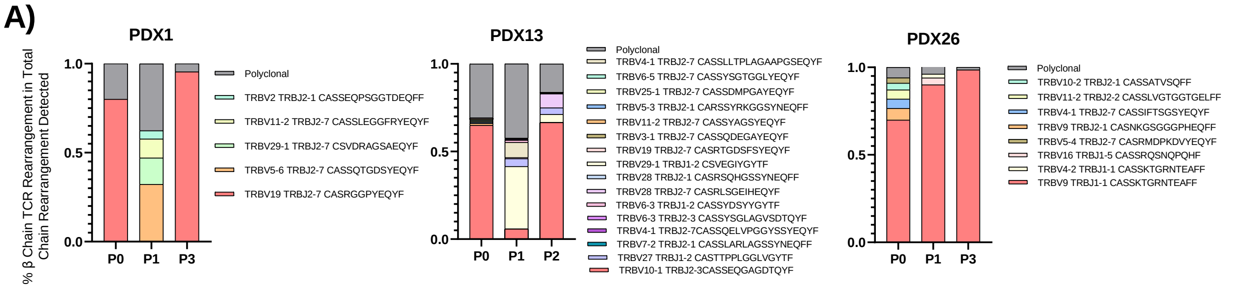

B)

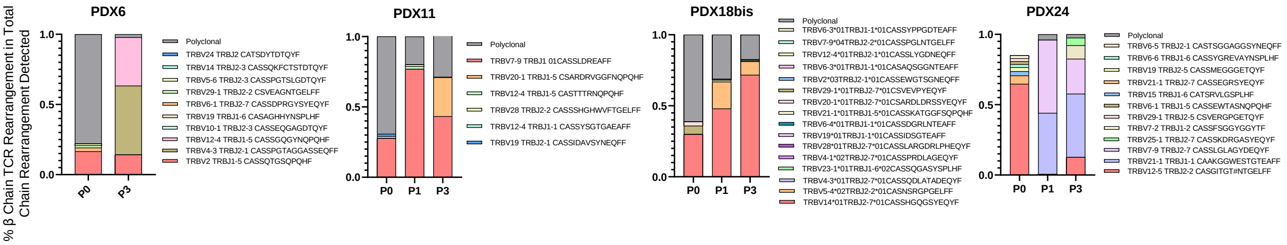

C)

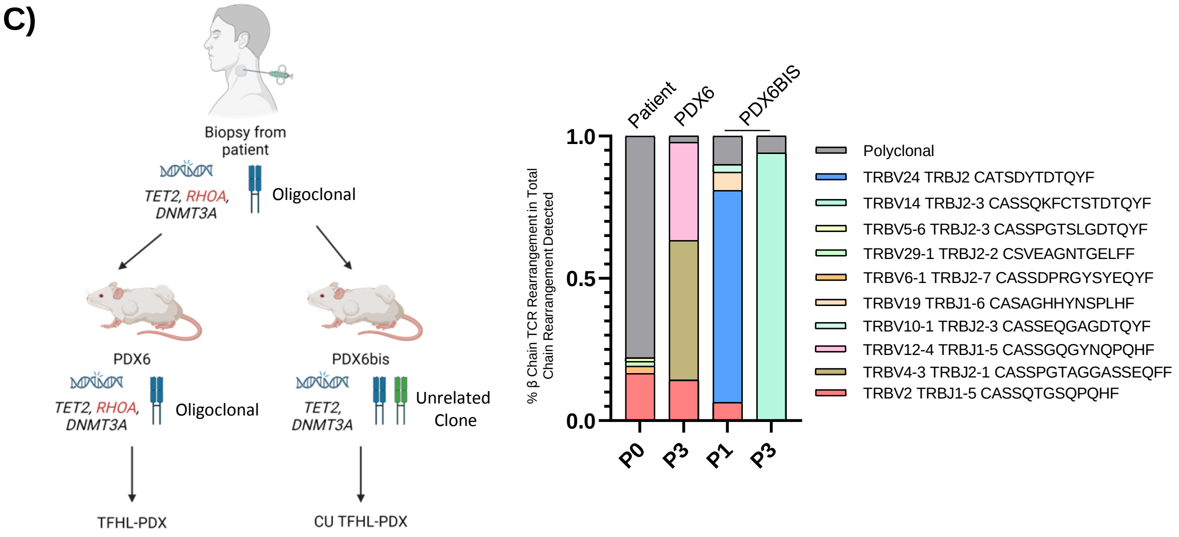

D)

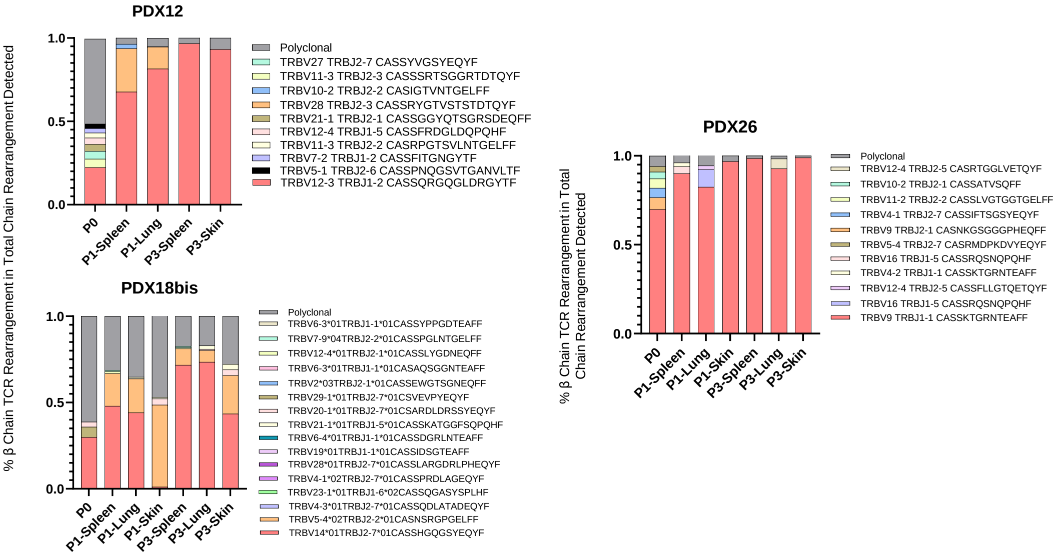

Supplementary Figure 3 – TCR Clonality of PDX Models

A) Clonal evolution of clonal TFHL PDX1, PDX13 and PDX26 models. Percentage of β Chain TCR rearrangement in total chain rearrangement detected on spleen cells was presented. The neoplastic subclones in these models expanded with a competitive advantage and became the dominant clone.

B) Clonal evolution of oligoclonal TFHL PDX6, PDX11, PDX18BIS and PDX24 models. Percentage of β Chain TCR rearrangement in total chain rearrangement detected on spleen cells was presented. The coexistence of oligoclonal tumors, with the coexistence of the neoplastic clone and one or several clone that were not expanded in the primary tumor was observed.

C) Schematic representation and clonal evolution of TFHL-PDX6 and CU-TFHL-PDX6bis generated from Patient 6. Percentage of β Chain TCR rearrangement in total chain rearrangement detected was presented spleen cells of PDX6 and PDX6bis.

D) Clonal composition of different organs. Percentage of β Chain TCR rearrangement in total chain rearrangement detected was presented for spleen, lung and skin of PDX12, PDX18bis and PDX26.

P0: Patient tumor P1: Passage 1, P2: Passage 2, P3: Passage 3

Supplementary Figure 4 – IDH2 mutated TFHL-PDX

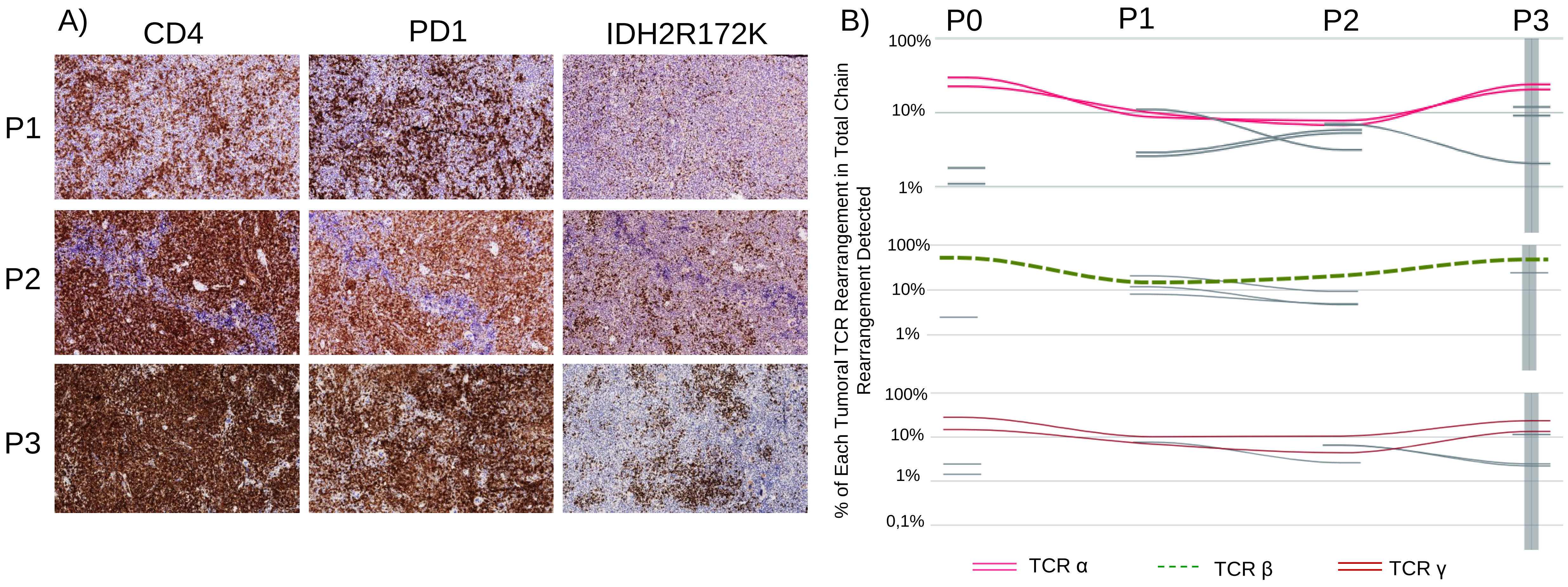

A) Pathology of spleen tissue from PD26 mice CD3 (20X), PD1 (20X), and IDH2 R172K (20X)
B) The clonal evolution of PDX26 model. Tumoral clone was caracterised by 2 alpha (pink lines), 1 beta (green line) and 2 gamma beta (dark red lines) chain gene rearrangements. Specific tumoral rearrangements were then tracked in passage 1, passage 2 and passge 3 PDX mice. Gray lines represents different clones.

P0: Patient P1: Passage 1, P2: Passage 2, P3: Passage 3

Supplementary Figure 5 – EBV+ B Cell Lymphoma

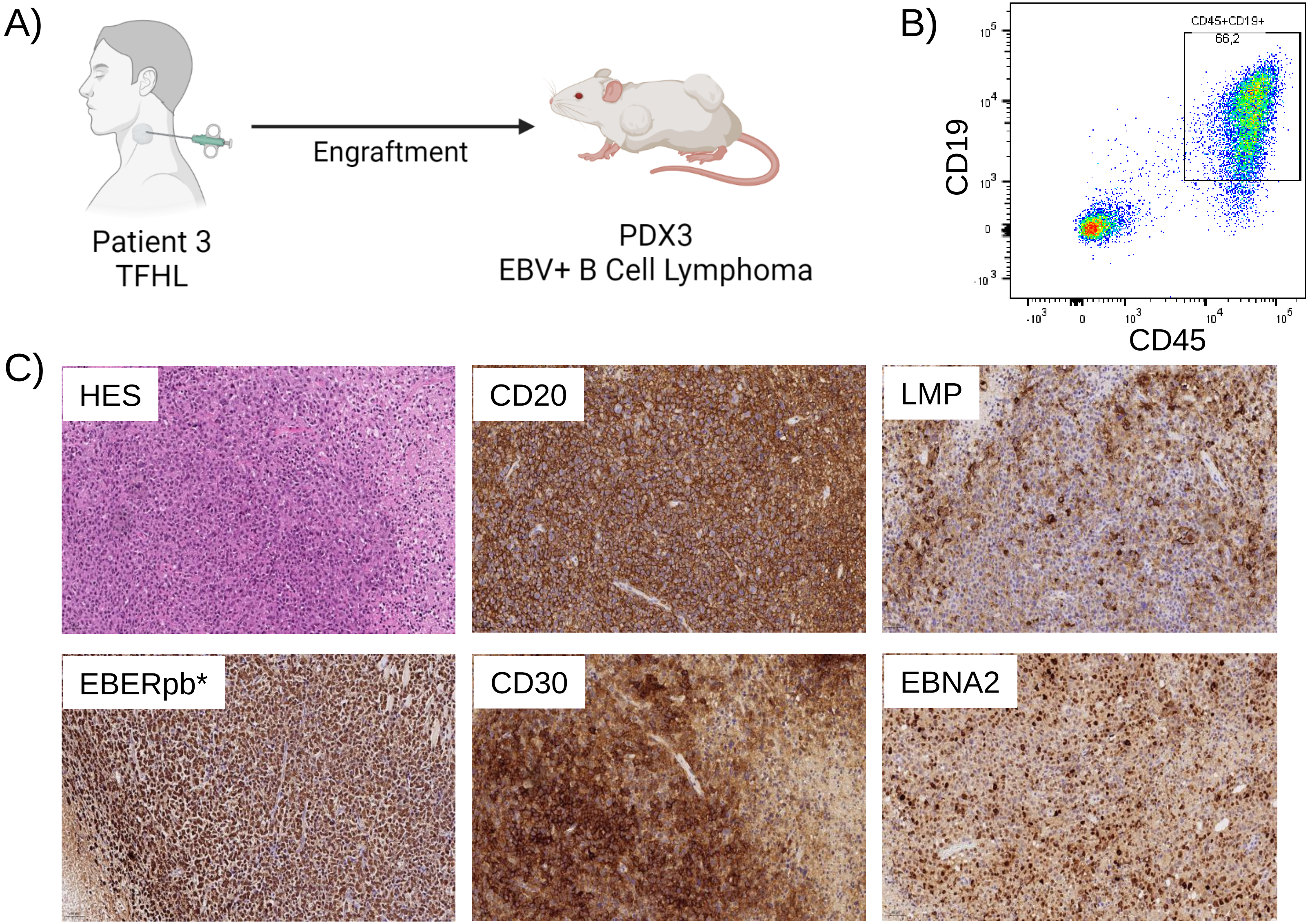

D)

Mutation	DNMT3A	TET2	CD79B
VAF	48%	40%	50%

A) Schematic representation of EBV+ B Cell Lymphoma PDX generation from TFHL patient
B) FACS phenotype of splenocytes CD45+CD19+
C) Pathology of tumor HES (20X), CD20 (20X), CD30 (20X), LMP (20X), EBNA2 (20X) and EBER (20X)
D) Mutational profile of tumor cells (Some mutations of B cell lymphoma panel could not be confirmed due to the technical problem, e.g. ARID1A)

Supplementary Figure 6 – Azacitidine treatment response

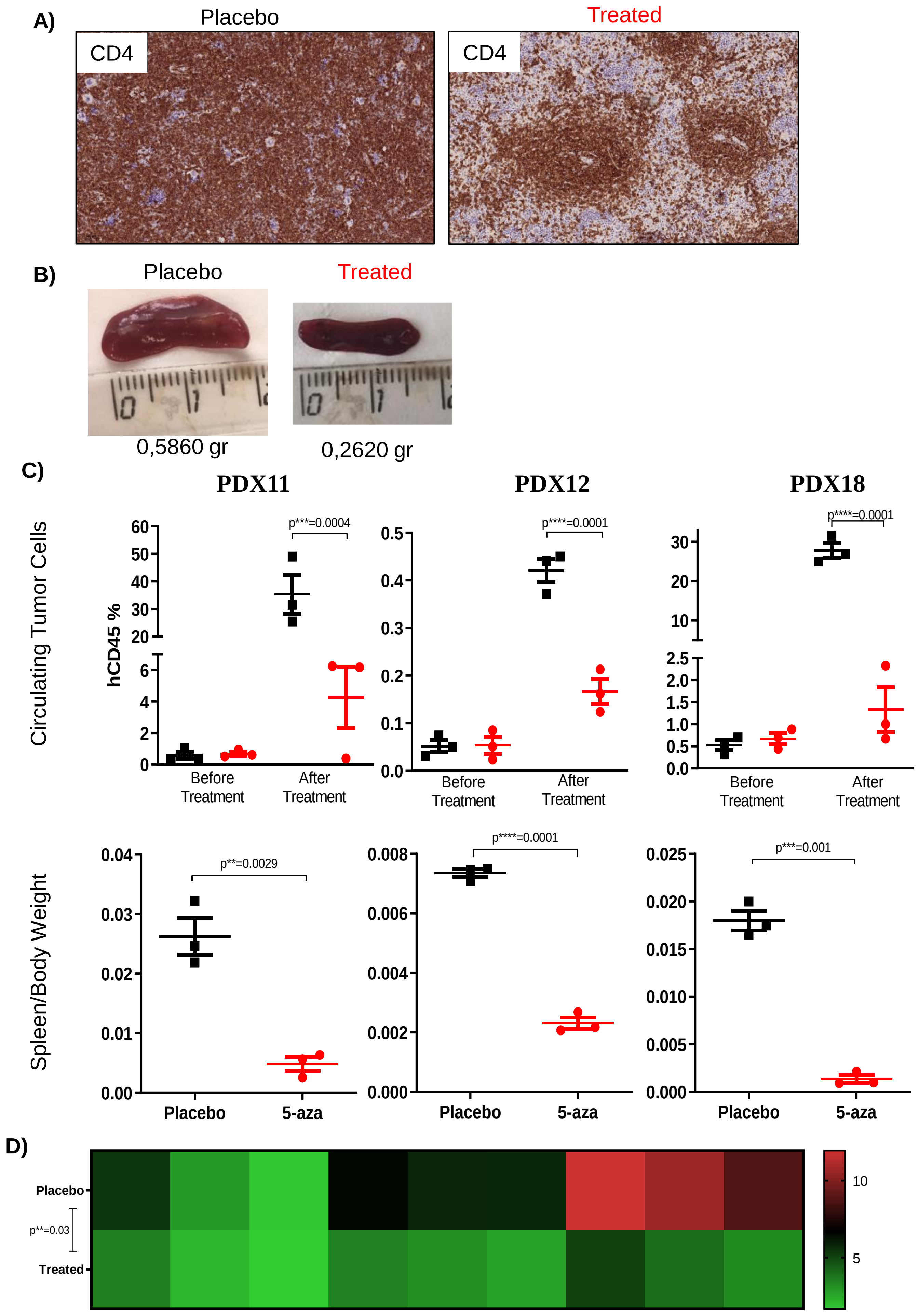

Supplementary Figure 6 – Azacitidine treatment response

A) IHC phenotype of splenocytes aza vs Placebo treated mice. CD4(20X) staining showed less tumor cells in azacitidine treated mice.

B) Splenomegaly difference aza vs Placebo treated mice

C) Treatment efficacy on PDX11, PDX12 and PDX18. Shown here with decreased circulating tumor cell and spleen/body weight ratio on azacitidine treated mice.

D) Heapmap of ICOS expression. MFI of ICOS expression was normalized to healthy PBMC.

Supplementary Figure 7. Transcriptomic effects and functional analysis of azacitidine treatment

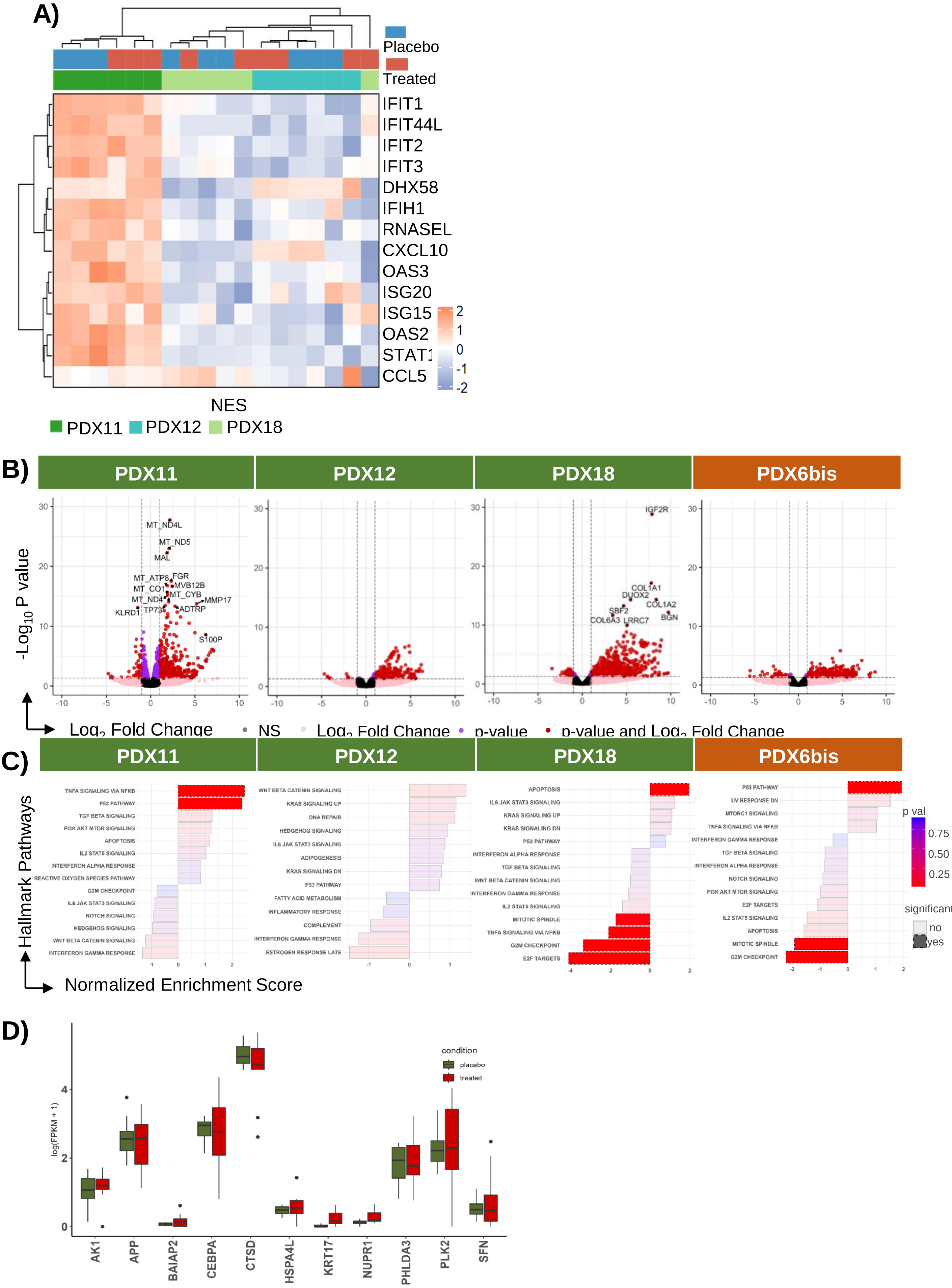

A) The heatmaps of selected viral mimicry associated genes of responsive models

B) The volcanoplots of expression change of protein-coding genes in the models as compared to placebo after 5-aza treatment.

C) Selected hallmark gene set enrichment of Differentially Expressed Genes (i.e. |log2(foldchange)| > 1 and p-value < 0.05). Hallmarks with boxed filled red and in black dashed line showed significant changes.

D) Comparison of the expression of genes found to be associated with hallmarks p53 pathway in DEGs set of sensitive models.

Supplementary Figure 8 – p53 mediated apoptosis effect of azacitidine

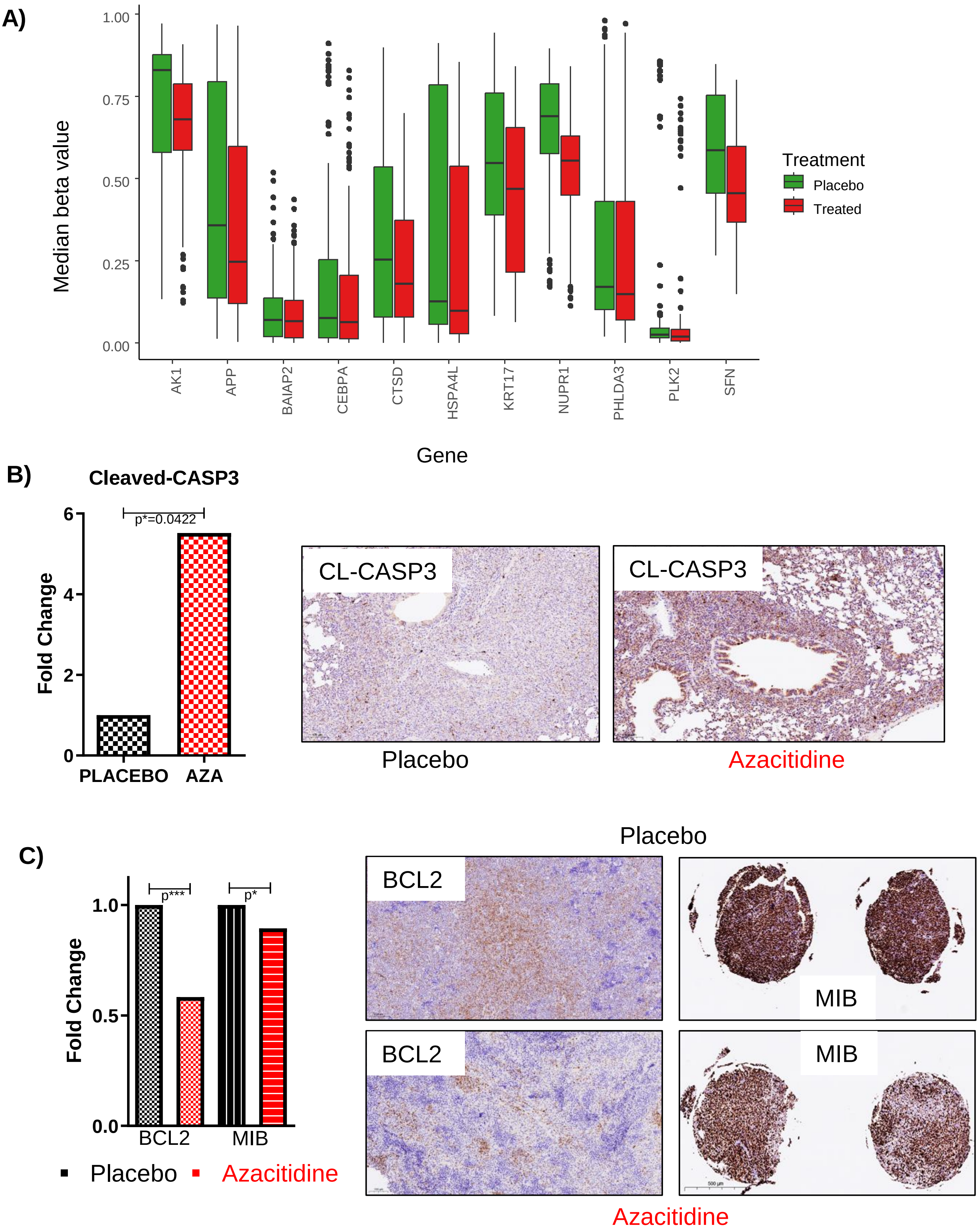

A) Median promoter DNA methylation of the p53-related differentially expressed genes for placebo and azacitidine treated sensitive models.
B)Azacitidine increases apoptosis shown here with cleaved caspase 3 staining
C) Azacitidine decreases anti-apoptotic protein BCL2 and proliferation shown here with MIB/Ki-67 staining
Positive cells were quantified via Quapath software and normalized to CD4+ cell count and placebo
