# Supplementary material for: Induction of p53-mediated apoptosis by azacitidine in patient-derived xenograft follicular helper T-cell lymphoma model
Source: Leukemia. 2025 May 20;39(7):1744–55. doi: 10.1038/s41375-025-02628-0 (PMC12208902; doi:10.1038/s41375-025-02628-0)
Supplement: Supplementary file 2 — Supplementary Table [file 41375_2025_2628_MOESM2_ESM.pdf]

			patient tumor	PDX1-P1-_Spleen	PDX1-P3_Spleen
mutational status (NGS)	gene	p.AA	variant allele frequency (%)		
	CD28	0,00000	0,00000	0,00000	0,00000
	IDH2	0,00000	0,00000	0,00000	0,00000
	IDH2	0,00000	0,00000	0,00000	0,00000
	RHOA	0,00000	0,00000	0,00000	0,00000
	VAV1	0,00000	0,00000	0,00000	0,00000
	TET2	0,00000	0,00000	0,00000	0,00000
	TET2	0,00000	0,00000	0,00000	0,00000
TCRbeta repertoire	beta gene rearrangement	Junction AA (majority coding sequences)	% of rearrangement withing all TCRB gene rearrangements		
	**TRBV19*03TRBJ2-7*01**	**CASRGGPYEQYF**	**0,81068**	**0,00000**	**0,93249**
	TRBV12-5*01TRBJ1-5*01	CASAKDRGR#NQPQHF	0,01226	0,00000	0,00000
	TRBV5-6*01TRBJ1-5*01	CASSSTRGLGQPQHF	0,00894	0,00000	0,00000
	TRBV3-1*01TRBJ2-7*01	CASSQVPRTGGEQYF	0,00856	0,00000	0,00000
	TRBV5-4*01TRBJ2-7*01	CASSLSLGDYEQYF	0,00843	0,00000	0,00000
	TRBV21-1*01TRBJ1-2*01	CASREIGQ#YGYTF	0,00805	0,00000	0,00000
	TRBV20-1*04TRBJ2-7*01	CSARPRRAP#SYEQYF	0,00792	0,00000	0,00000
	TRBV19*03TRBJ2-7*01	CASRGCPYEQYF	0,00728	0,00000	0,00008
	TRBV6-3*01TRBJ1-3*01	CASSYPTGTAGNTIYF	0,00715	0,00000	0,00000
	TRBV5-6*01TRBJ2-7*01	CASSQTGDSYEQYF	0,00000	0,32173	0,00000
	TRBV21-1*01TRBJ1-1*01	CASSKAPG#NTEAFF	0,00000	0,20982	0,00000
	TRBV29-1*01TRBJ2-7*01	CSVDRAGSAEQYF	0,00000	0,14890	0,00000
	TRBV11-2*01TRBJ2-7*01	CASSLEGGFRYEQYF	0,00000	0,10631	0,00000
	TRBV27*01TRBJ2-6*01	CASRGIQ#SGANVLTF	0,00000	0,06901	0,00000
	TRBV2*03TRBJ2-1*01	CASSEQPSGGTDEQFF	0,00000	0,04694	0,00000
	TRBV11-3*01TRBJ1-3*01	CASSLEGRI#SGNTIYF	0,00000	0,02052	0,00000
	TRBV29-1*02TRBJ1-4*01	CSVEGGDRATNEKLFF	0,00000	0,00901	0,00000
	TRBV11-2*01TRBJ1-6*01	CASSTPRDRGSPLHF	0,00000	0,00808	0,00000
	TRBV3-2*03TRBJ2-7*01	CASSQTGDSYEQYF	0,00000	0,00715	0,00000
	TRBV7-1*01TRBJ2-3*01	CASSSNLGSTDTQYF	0,00000	0,00062	0,00000
TCRalpha repertoire	alpha gene rearrangement	Junction AA (around half non coding)	% of rearrangement withing all TCRA gene rearrangements		
	TRAV39*01TRAJ50*01	CAVDSMKTSYDKVIF	0,69524	0,00465	0,71611
	TRAV5*01TRAJ5*01	CAE*GTGRRALTF	0,18540	0,00000	0,25674
	TRAV1-2*01TRAJ11*01	CAVKSGYSTLTF	0,00384	0,00000	0,00000
	TRAV17*01TRAJ45*01	CATPGGGADGLTF	0,00322	0,00000	0,00000

	TRAV12-3*01TRAJ49*01	CAMTP#NTGNQFYF	0,00304	0,00000	0,00000
	TRAV4*01TRAJ5*01	CLVGVH#DTGRRALTF	0,00257	0,00000	0,00000
	TRAV23/DV6*01TRAJ44*01	CAASL#NTGTASKLTF	0,00217	0,00000	0,00000
	TRAV5*01TRAJ6*01	CAETSGGSYIPTF	0,00189	0,00000	0,00000
	TRAV20*01TRAJ10*01	CAVSILTGGGNKLTF	0,00180	0,00000	0,00000
	TRAV21*02TRAJ3*01	FL#SSASKIIF	0,00177	0,00000	0,00000
	TRAV12-2*03TRAJ52*01	CAVNRGTSYGKLTF	0,00000	0,18442	0,00000
	TRAV12-1*01TRAJ28*01	CVV#YS	0,00000	0,16972	0,00000
	TRAV5*01TRAJ48*01	CAEMQLNFGNEKLTF	0,00000	0,11181	0,00000
	TRAV8-2*01TRAJ23*01	CVVSLNP#YNQGGKLIF	0,00000	0,08078	0,00000
	TRAV8-6*01TRAJ28*01	CAVSLG#S	0,00000	0,07487	0,00000
	TRAV13-1*01TRAJ37*01	CAASI*#TGKLIF	0,00000	0,06646	0,00000
	TRAV5*01TRAJ29*01	CAES#SGNTPLVF	0,00000	0,04485	0,00000
	TRAV13-2*02TRAJ48*01	CAEMQLNFGNEKLTF	0,00000	0,03304	0,00000
	TRAV8-6*02TRAJ50*01	CAVSD*##TSYDKVIF	0,00000	0,03028	0,00000
	TRAV24*01TRAJ4*01	CV#SGGYNKLIF	0,00000	0,02814	0,00000
	TRAV5*01TRAJ36*01	CAEREETGANNLFF	0,00000	0,02513	0,00000
	TRAV21*01TRAJ30*01	CAVLNRDDKIIF	0,00000	0,02324	0,00000
	TRAV5*01TRAJ34*01	CAESPLDKLIF	0,00000	0,02010	0,00000
	TRAV8-6*01TRAJ50*01	CAVSD*R#TSYDKVIF	0,00000	0,01847	0,00000
	TRAV35*01TRAJ40*01	not found	0,00000	0,01834	0,00000
	TRAV12-1*01TRAJ16*02	CVVTEGDGLLFARG	0,00000	0,01332	0,00000
	TRAV26-1*01TRAJ36*01	CIVRPAANNLFF	0,00000	0,01269	0,00000
	TRAV16*01TRAJ23*01	CALSRYQGGKLIF	0,00000	0,01231	0,00000
	TRAV29/DV5*02TRAJ30*01	CAA#NRDDKIIF	0,00000	0,00678	0,00000
TCRgamma repertoire	**gamma gene rearrangement**	**Junction AA (Mostly non coding)**	**% of rearrangement withing all TCRG gene rearrangements**		
	**TRGV8*01TRGJ1*02**	**CATWDRVG#YKKLF**	**0,44207**	**0,00000**	**0,47292**
	**TRGV2*01TRGJ1*02**	**CATWDGPRRKLF**	**0,41885**	**0,00000**	**0,48711**
	TRGV8*01TRGJ1*01	not found (uncoding)	0,00762	0,00000	0,00000
	TRGV11*01TRGJ1*02	CACWIS#YYKKLF	0,00529	0,00000	0,00000
	TRGV2*01TRGJ1*01	CATWDGW#KLF	0,00481	0,00000	0,00000
	TRGV1*01TRGJ1*02	CATWDWYYKKLF	0,00367	0,00000	0,00000

TRGV2*01TRGJ1*02	CATWDGPRQKLF	0,00200	0,00000	0,00557
TRGV8*01TRGJ1*01	CATWD#NYYKKLF	0,00189	0,03100	0,00000
TRGV3*01TRGJ1*01	CATWDRP*IA#YKKLF	0,00000	0,26375	0,00000
TRGV3*02TRGJ1*01	CATWVPWDKKLF	0,00000	0,21841	0,00000
TRGV5*01TRGJ1*02	CATWDAY#YYKKLF	0,00000	0,17370	0,00000
TRGV10*02TRGJP1*01	not found (uncoding)	0,00000	0,10142	0,00000
TRGV2*01TRGJ1*02	CATWDGPCYKKLF	0,00000	0,04518	0,00000
TRGV10*02TRGJ1*02	CAAWDP#NYYKKLF	0,00000	0,03568	0,00000
TRGV2*01TRGJP1*01	CATWDGPQST#FKIF	0,00000	0,03521	0,00000
TRGV10*02TRGJP1*01	not found (uncoding)	0,00000	0,01994	0,00000
TRGV9*01TRGJ1*01	CALWEAQSPH*#YYKKLF	0,00000	0,01792	0,00000
TRGV2*02TRGJP2*01	CATWEA#SDWIKTF	0,00000	0,01667	0,00000
TRGV2*01TRGJ1*02	CATW#NYYKKLF	0,00000	0,00872	0,00000
TRGV10*02TRGJ1*01	CAAWGC*YWG#YKKLF	0,00000	0,00592	0,00000

			Patient tumor	PDX6-P3_spleen	PDX6BIS-P1_spleen	PDX6BIS-P3_spleen
**mutational status (NGS)**	**gene**	**p.AA**	**variant allele frequency (%)**			
	RHOA	p.G17V	0,127	0,436	0	0
	TET2	p.Q962X	0,388	0,903	0,738	0,998
	TET2	p.N1698fs	0,038	0,0072	0,0043	0
	DNMT3A	p.R882H	**0,206**	0,0058	0,389	0,554
**TCRbeta repertoire (NGS)**	**beta gene rearrangement**	**Junction AA**	**% of rearrangement withing all TCRB gene rearrangements**			
	**TRBV2*02TRBJ1-5*01**	**CASSQTGSQPQHF**	**0,1653465**	**0,1421725**	**0,063560629**	**0**
	TRBV27*01TRBJ1-4*01	CASKFGGDEKLFF	0,0259901	0	0	0
	TRBV6-1*01TRBJ2-7*01	CASSDPRGYSYEQYF	0,0256188	0	0	0
	TRBV29-1*01TRBJ2-2*01	CSVEAGNTGELFF	0,0170792	0	0	0
	TRBV5-6*01TRBJ2-3*01	CASSPGTSLGDTQYF	0,0121287	0	0	0
	TRBV14*01TRBJ2-3*01	CASSQKFCTSTDTQYF	0	0	0	0,940059134
	TRBV24/OR9-2*01TRBJ2-3*01	CATSDYTDTQYF	0	0	0,745526072	0
	TRBV19*03TRBJ1-6*02	CASAGHHYNSPLHF	0	0	0,064177723	0
	TRBV10-1*01TRBJ2-3*01	CASSEQGAGDTQYF	0	0	0,02630361	0
	TRBV20-1*04TRBJ2-3*01	CSVNRDY#STDTQYF	0	0	0,014501697	0
	TRBV4-3*01TRBJ2-1*01	CASSPGTAGGASSEQFF	0	0,4904153	0	0
	TRBV12-4*01TRBJ1-5*01	CASSGQGYNQPQHF	0	0,3450479	0	0
**TCRalpha repertoire**	**alpha gene rearrangement**	**Junction AA (around half non coding)**	**% of rearrangement withing all TCRA gene rearrangements**			
	**TRAV17*01TRAJ26*01**	**CATSLGGQNFVF**	**0,2240522**	**0,5734217**	**0**	**0**
	TRAV25*01TRAJ49*01	CAG#NTGNQFYF	0,0525321	0	0,015889232	0
	TRAV16*01TRAJ31*01	CALSHRNNNARLMF	0,0508482	0	0,004220997	0
	TRAV23/DV6*01TRAJ34*01	CAASTSTDKLIS	0,0243151	0,0729677	0	0
	TRAV12-2*02TRAJ50*01	CAGGETSYDKVIF	0,0087485	0	0	0
	TRAV6*01TRAJ32*02	CALGNGGATNKLIF	0,0061609	0	0	0
	TRAV8-1*01TRAJ37*02	CAVNGGSSNTGKLIF	0,0047644	0	0	0
	TRAV8-3*01TRAJ50*01	CAVGGEHASYDKVIF	0,0041894	0	0	0
	TRAV8-6*01TRAJ54*01	CAVSGTQKLVF	0,0041484	0	0	0
	TRAV39*01TRAJ58*01	CAVGS#*ETSGSRLTF	0,0041073	0	0	0
	TRAV14/DV4*03TRAJ53*01	CAMEGSNYKLTF	0,0040662	0	0	0
	TRAV12-1*01TRAJ9*01	CVV*HR#TGGFKTIF	0,0039841	0	0	0
	TRAV10*01TRAJ42*01	CVVSA#SYGGSQGNLIF	0,003943	0	0	0
	TRAV22*01TRAJ34*01	CAFWGDNTDKLIF	0,0037787	0	0	0

	TRAV20*01TRAJ59*01	CAVQTKEGNRKFTF	0,0036555	0	0	0
	TRAV8-4*01TRAJ49*01	CAVS#NTGNQFYF	0,0036144	0	0	0
	TRAV35*01TRAJ40*01	CAGHP#LPSGTYKYIF	0,003409	0	0	0
	TRAV12-2*03TRAJ4*01	CAVTSLFSGGYNKLIF	0,0033269	0	0	0
	TRAV12-2*03TRAJ33*01	CAVNG#NYQLIW	0,0032858	0	0	0
	TRAV12-2*03TRAJ11*01	CALNSGYSTLTF	0,0032448	0	0	0
	TRAV14/DV4*02TRAJ28*01	CAMRET	0,0031626	0	0	0
	TRAV41*01TRAJ42*01	CAA#GGSQGNLIF	0,0030805	0	0	0
	TRAV1-1*01TRAJ20*01	CAVRGSNDYKLSF	0	0,00687	0	0
	TRAV17*01TRAJ26*01	CATSFGGQNFVF	0	0,00572	0	0
	TRAV17*01TRAJ26*01	CATSLRGQNFVF	0	0,0046607	0	0
	TRAV12-3*01TRAJ22*01	CATLKPGSARQLTF	0	0,0042673	0	0
	TRAV14/DV4*02TRAJ7*01	CAMREDH*SKYYGSNRLAF	0	0,0028751	0	0
	TRAV17*01TRAJ26*01	CATSL#GQNFVF	0	0,0027238	0	0
	TRAV17*01TRAJ26*01	CAMSLGGQNFVF	0	0,0022698	0	0
	TRAV8-4*01TRAJ29*01	CAVSP#NSGNTPLVF	0	0	0,430246001	0
	TRAV5*01TRAJ40*01	CAERDSGTYKYIF	0	0	0,340556526	0
	TRAV3*02TRAJ29*01	not found	0	0	0,030057803	0
	TRAV20*01TRAJ34*01	CAVLYNTDKLIF	0	0	0,030030918	0
	TRAV1-2*01TRAJ27*01	CAVRDGTNAGKSTF	0	0	0,01653448	0
	TRAV12-3*01TRAJ44*01	LE#PALPVNSHL	0	0	0,011453152	0
	TRAV34*01TRAJ45*01	CGAAL#S	0	0	0,010969216	0
	TRAV13-1*01TRAJ26*01	CAASKG#DNYGQNFVF	0	0	0,007635435	0
	TRAV41*01TRAJ58*01	CAVR#TSGSRLTF	0	0	0,004409195	0
	TRAV2*01TRAJ24*02	CAVLTDSWGKLQF	0	0	0,003495093	0
	TRAV39*01TRAJ59*01	CAVDRKEGNRKFTF	0	0	0	0,61443337
	TRAV12-3*01TRAJ48*01	CAMREGFGNEKLTF	0	0	0	0,372942045
TCRgamma repertoire	**gamma gene rearrangement**	**Junction AA (Mostly non coding)**	**% of rearrangement withing all TCRG gene rearrangements**			
	**TRGV10*02TRGJ1*01**	**CAAWEGKKLF**	**0,3098092**	**0,5808914**	**0**	**0**
	**TRGV5*01TRGJ1*02**	**CATWEV#NYYKKLF**	**0,1671028**	**0,353502**	**0**	**0**
	TRGV10*02TRGJ1*02	CAACAPS#YYKKLF	0,059556	0	0,024517766	0
	TRGV2*01TRGJ1*02	CATWDGP#NYYKKLF	0,041744	0	0,016091371	0
	TRGV5P*01TRGJ1*02	CATWGGP#YKKLF	0,0079774	0	0	0

TRGV5*01TRGJ2*01	CATWD#YKKLF	0,0076819	0	0	0
TRGV2*01TRGJ1*02	CATWDAP#KKLF	0,0062046	0	0	0
TRGV2*01TRGJP2*01	CATWDGP#SSDWIKTF	0,0047273	0	0	0
TRGV8*01TRGJ1*02	CATRR#LF	0,0045163	0	0	0
TRGV2*01TRGJ2*01	CATWDGL#KLF	0,004263	0	0	0
TRGV10*02TRGJP1*01	CAAWL#TGWFKIF	0,0039676	0	0	0
TRGV9*01TRGJ1*02	CALCLSVR#KLF	0,003841	0	0	0
TRGV5*01TRGJ1*01	not found (uncoding)	0,0010552	0,0111011	0	0
TRGV10*02TRGJ1*02	CAAWGYYKKLF	0	0,0291029	0	0
TRGV3*02TRGJ1*02	CATWDKT#NYYKKLF	0	0	0,494720812	0
TRGV4*01TRGJ1*02	CATCETPAE#YKKLF	0	0	0,248680203	0
TRGV4*02TRGJ1*02	CATWG#KLF	0	0	0,041269036	0
TRGV5P*01TRGJ1*02	CATWG#KLF	0	0	0,022538071	0
TRGV5P*01TRGJ1*02	CATWG#KLF	0	0	0,020304569	0
TRGV2*01TRGJP1*01	CATWDVSSWFKIF	0	0	0,016649746	0
TRGV4*02TRGJ1*02	CATWE#KLF	0	0	0,00857868	0
TRGV4*02TRGJ1*01	not found (uncoding)	0	0	0,008477157	0
TRGV8*01TRGJ1*02	CATPC#YYKKLF	0	0	0	0,487073751
TRGV7*01TRGJ1*02	not found (uncoding)	0	0	0	0,413481364
TRGV7*01TRGJ1*01	not found (uncoding)	0	0	0	0,038984933
TRGV1*01TRGJ2*01	CATWDRR#YKKLF	0	0	0	0,014655036
TRGV8*01TRGJ1*01	not found (uncoding)	0	0	0	0,009706582
TRGV8*01TRGJ1*01	not found (uncoding)	0	0	0	0,005233941
TRGV2*02TRGJ1*02	CATSC#YYKKLF	0	0	0	0,003203807

mutational status (NGS)	gene	p.AA	Patient tumor	PDX11-P1_Spleen	PDX11-P3_Spleen
			variant allele frequency (%)		
	CD28	p.D124E	0,01000	0,00000	0,00000
	IDH2	p.R172W	0,00800	0,00000	0,00000
	IDH2	p.R172G	0,00800	0,00000	0,00000
	RHOA	p.G17V	0,10800	0,38100	0,22900
	TET2	p.S602fs	0,11300	0,44300	0,52200
	TET2	p.D1402fs	0,15200	0,45400	0,46900
	DNMT3A	p.V328D	0,00000	0,39100	0,45800

TCRbeta repertoire	beta gene rearrangement	Junction AA	% of rearrangement withing all TCRB gene rearrangements		
	TRBV7-9*01TRBJ1-1*01	CASSLDREAFF	0,27712	0,76901	0,43279
	TRBV19*01TRBJ2-1*01	CASSIDAVSYNEQFF	0,01670	0,00000	0,00000
	TRBV5-6*01TRBJ2-7*01	CASSLASQGYYEQYF	0,01634	0,00000	0,00000
	TRBV12-4*01TRBJ1-1*01	CASSYSGTGAEAFF	0,01397	0,00000	0,00000
	TRBV4-1*01TRBJ1-2*01	CASSQDTGTGGASGYTF	0,01090	0,00000	0,00000
	TRBV3-1*01TRBJ1-4*01	CASNEGSGTTKLFF	0,00959	0,00000	0,00000
	TRBV23-1*01TRBJ1-2*01	CASSRG#NYGYTF	0,00959	0,00000	0,00000
	TRBV4-1*01TRBJ1-3*01	CASSQEEARQNTIYF	0,00947	0,00000	0,00000
	TRBV4-3*01TRBJ2-1*01	CASSQAGSGGVHEQFF	0,00876	0,00000	0,00000
	TRBV28*01TRBJ2-7*01	CASSSYHIAGSSYEQYF	0,00865	0,00000	0,00000
	TRBV28*01TRBJ2-7*01	CASSLPSSYEQYF	0,00805	0,00000	0,00000
	TRBV20/OR9-2*01TRBJ1-6*02	CSARDPDRGLNSPLHF	0,00793	0,00000	0,00000
	TRBV4-3*01TRBJ2-7*01	CASSQVTGGRYEQYF	0,00758	0,00000	0,00000
	TRBV21-1*01TRBJ1-2*01	CASSKEGEGHGYTF	0,00734	0,00000	0,00000
	TRBV7-9*01TRBJ1-1*01	CASSSGGEAFF	0,00616	0,00000	0,00000
	TRBV29-1*03TRBJ1-4*01	CSVGTGGTNEKLFF	0,00580	0,00000	0,00000
	TRBV19*01TRBJ2-7*01	CASSIVAGGYEQYF	0,00533	0,00000	0,00000
	TRBV6-1*01TRBJ2-7*01	CASSQDRTASYEQYF	0,00533	0,00000	0,00000
	TRBV7-2*01TRBJ1-2*01	CASSLAGGWDSTNYGYTF	0,00509	0,00000	0,00000
	TRBV6-5*01TRBJ1-2*01	CASSPLGGFDGYTF	0,00474	0,00000	0,00000
	TRBV11-2*01TRBJ1-2*01	CASSLDREAFFGQGTRLTVVGKTFFRFFCRSVTGKSGSTVSLLEWLYSYV#NYGYTF	0,00308	0,01137	0,00041
	TRBV12-4*01TRBJ1-2*01	CASRATGGYGISGYTF	0,00296	0,00000	0,00000
	TRBV5-1*01TRBJ2-7*01	CASSSPGQGVYEQYF	0,00261	0,00000	0,00000
	TRBV9*01TRBJ1-3*01	CASSSSDLGNTIYF	0,00249	0,00000	0,00000
	TRBV19*01TRBJ2-7*01	CASSIGGKGYEQYF	0,00118	0,00000	0,00000

	TRBV20-1*05TRBJ2-5*01	CSARKGGGKETQYF	0,00107	0,00000	0,00000
	TRBV7-8*03TRBJ2-2*01	CASSNPGELFF	0,00024	0,00687	0,00000
	TRBV20-1*01TRBJ1-5*01	CSARDRVGGFNQPQHF	0,00000	0,00000	0,27662
	TRBV19*01TRBJ1-2*01	CASSIDPNYGYTF	0,00000	0,00247	0,14927
	TRBV6-1*01TRBJ1-5*01	CASSERGKASGS#INQPQHF	0,00000	0,02820	0,09919
	TRBV10-1*01TRBJ1-6*02	CASSVEDSS#YNSPLHF	0,00000	0,00000	0,00320
	TRBV12-4*01TRBJ1-5*01	CASTTTRNQPQHF	0,00000	0,01895	0,00222
	TRBV28*01TRBJ1-2*01	CASSRAFPYGYTF	0,00000	0,01269	0,00000
	TRBV28*01TRBJ2-2*01	CASSSHGHWVFTGELFF	0,00000	0,01216	0,00000
	TRBV6-1*01TRBJ1-5*01	CASSWTGGGGQPQHF	0,00000	0,00917	0,00000
	TRBV2*01TRBJ1-6*02	CASSEVKKGIGTLHF	0,00000	0,00767	0,00000
	TRBV18*01TRBJ1-1*01	CASSPRGTEAFF	0,00000	0,00767	0,00000
	TRBV7-9*01TRBJ1-2*01	CASSHRAGTTS#YGYTF	0,00000	0,00590	0,00000
	TRBV4-2*01TRBJ2-4*01	CASSQSDRGSDIQYF	0,00000	0,00555	0,00000
**TCRalpha repertoire**	**alpha gene rearrangement**	**Junction AA (around half non coding)**	**% of rearrangement withing all TCRA gene rearrangements**		
	**TRAV35*01TRAJ17*01**	**CAG#KAAGNKLTF**	**0,21961**	**0,49053**	**0,23783**
	**TRAV8-3*01TRAJ44*01**	**CASPEKTGTASKLTF**	**0,15181**	**0,36236**	**0,19456**
	TRAV21*01TRAJ38*01	CAV#WQQP*ADL	0,01223	0,00000	0,00000
	TRAV24*01TRAJ45*01	CAFGGGS	0,00851	0,00000	0,00000
	TRAV6*01TRAJ15*01	CALG#NQAGTALIF	0,00754	0,00000	0,00000
	TRAV2*01TRAJ37*02	CAVEERGSSSNTGKLIF	0,00744	0,00000	0,00000
	TRAV27*01TRAJ53*01	CAGVKDGNYKLTF	0,00718	0,00000	0,00000
	TRAV12-2*02TRAJ36*01	CAVQTGANNLFF	0,00606	0,00000	0,00000
	TRAV1-2*01TRAJ15*01	CAVISRQAGTALIF	0,00581	0,00000	0,00000
	TRAV12-2*01TRAJ37*01	CAVNTGGTGKLIF	0,00550	0,00000	0,00000
	TRAV12-1*01TRAJ41*01	CVVDSGYALNF	0,00545	0,00000	0,00000
	TRAV26-1*01TRAJ9*01	CIAR#TGGFKTIF	0,00504	0,00000	0,00000
	TRAV19*01TRAJ30*01	CALSESKRDDKIIF	0,00479	0,00000	0,00000
	TRAV12-2*01TRAJ42*01	CAVNMG#YGGSQGNLIF	0,00474	0,00000	0,00000
	TRAV22*01TRAJ40*01	CAGVHLSGTYKYIF	0,00423	0,00000	0,00000
	TRAV12-2*02TRAJ22*01	CAVSAQLTF	0,00402	0,00000	0,00000
	TRAV5*01TRAJ30*01	CAGFF#RDDKIIF	0,00377	0,00000	0,00000

TRAV1-1*02TRAJ16*02	CAVLRFSDGQKLLFARG	0,00367	0,00000	0,00000
TRAV38-1*01TRAJ29*01	CAFMKPYSGNTPLVF	0,00362	0,00000	0,00000
TRAV14/DV4*03TRAJ40*01	CAL#TSGTYKYIF	0,00321	0,00000	0,00000
TRAV41*01TRAJ61*01	CAVRF#NRVNRKLTF	0,00316	0,00000	0,00000
TRAV8-1*01TRAJ32*02	CAAYPG#GGATNKLIF	0,00311	0,00194	0,00000
TRAV21*01TRAJ3*01	CAVP#DSSASKIIF	0,00311	0,00000	0,00000
TRAV26-2*01TRAJ29*01	CIYSGNTPLVF	0,00301	0,00000	0,00000
TRAV13-1*01TRAJ16*01	CAATPLGDGQKLLF	0,00295	0,00000	0,00000
TRAV19*01TRAJ48*01	CALSDPFRNEKLTF	0,00280	0,00000	0,00000
TRAV8-1*01TRAJ32*02	CAVS#NYGGATNKLIF	0,00275	0,00000	0,00000
TRAV1-1*01TRAJ34*01	CAVRDQVNTDKLIF	0,00265	0,00000	0,00000
TRAV9-1*01TRAJ43*01	CALRTNNNDMRF	0,00260	0,00000	0,00000
TRAV10*01TRAJ32*01	CVVSA#GGATNKLIF	0,00260	0,00000	0,00000
TRAV25*01TRAJ26*01	CAGVG#GQNFVF	0,00255	0,00000	0,00000
TRAV5*01TRAJ10*01	CAELFTGGGNKLTF	0,00239	0,00000	0,00000
TRAV14/DV4*02TRAJ42*01	CAMREASNYGGSQGNLIF	0,00234	0,00000	0,00000
TRAV38-1*01TRAJ39*01	CAFRNAGNMLTF	0,00224	0,00000	0,00000
TRAV14/DV4*02TRAJ52*01	CAMRAQNYGKLTF	0,00214	0,00000	0,00000
TRAV3*01TRAJ50*01	CAVRDMTTSYDKVIF	0,00209	0,00000	0,00000
TRAV5*01TRAJ20*01	CAEKPMDKLSF	0,00087	0,00272	0,00000
TRAV17*01TRAJ8*01	CATDGGNTGFQKLVF	0,00036	0,00000	0,00000
TRAV29/DV5*01TRAJ33*01	CAASANSNYQLIW	0,00010	0,00000	0,00000
TRAV24*01TRAJ34*01	CAFNTDKLIF	0,00010	0,00000	0,00000
TRAV25*01TRAJ21*01	GGGVYNFNKFYF	0,00010	0,00622	0,00003
TRAV8-3*01TRAJ33*01	CAVGAE#DSNYQLIW	0,00005	0,00000	0,00000
TRAV22*01TRAJ23*01	CAALIYNQGGKLIF	0,00000	0,00387	0,19319
TRAV13-2*01TRAJ20*01	CAENMSSNDYKLSF	0,00000	0,00439	0,06419
TRAV35*01TRAJ40*01	CAGQLVSSGTYKYIF	0,00000	0,00611	0,00000
TRAV6*01TRAJ38*01	CALG#CWQQP*ADL	0,00000	0,00406	0,00000
TRAV19*01TRAJ20*01	CALHDDYKLSF	0,00000	0,00264	0,00003
TRAV14/DV4*02TRAJ28*01	CAMREGFYS	0,00000	0,00421	0,00000
TRAV13-1*01TRAJ29*01	CAPDSGNTPLVF	0,00000	0,00294	0,00000
TRAV17*01TRAJ53*01	CATEGGGHNSGGSNYKLTF	0,00000	0,00428	0,00000

	TRAV17*01TRAJ36*01	CATEKTGANNLFF	0,00000	0,00335	0,00000
	TRAV20*01TRAJ26*01	CAV*GNYGQNFVF	0,00000	0,00443	0,09936
	TRAV12-2*02TRAJ22*01	CAVGRKPN#SSGSARQLTF	0,00000	0,00890	0,00000
	TRAV8-1*01TRAJ24*02	CAVIVDSWGKLQF	0,00000	0,00421	0,00000
	TRAV20*01TRAJ58*01	CAVPRETSGSRLTF	0,00000	0,00350	0,00000
	TRAV12-2*02TRAJ23*01	CAVS#NQGGKLIF	0,00000	0,00000	0,17203
	TRAV38-2/DV8*01TRAJ48*01	CAYRRR#GNEKLTF	0,00000	0,00287	0,00000
	TRAV12-1*01TRAJ37*02	CVVNA#SSNTGKLIF	0,00000	0,00346	0,00000
TCRgamma repertoire	**gamma gene rearrangement**	**Junction AA (Mostly non coding)**	**% of rearrangement withing all TCRG gene rearrangements**		
	**TRGV5*01TRGJ1*02**	**CATWDRQ#YYKKLF**	**0,19307**	**0,60188**	**0,25810**
	TRGV10*02TRGJ1*02	CAAWDPNLG#YYKKLF	0,01680	0,00000	0,00000
	TRGV10*02TRGJP2*01	CAAWDFR#IKTF	0,01538	0,00000	0,00000
	TRGV8*01TRGJ1*02	CATWD#YYKKLF	0,00886	0,00000	0,00000
	TRGV10*02TRGJ2*01	CAAWDYFMNLT#KKLF	0,00851	0,00000	0,00000
	TRGV8*01TRGJ1*02	CATWDR#NYYKKLF	0,00801	0,00000	0,00000
	TRGV4*01TRGJ1*02	CATWGWGVHKKLF	0,00765	0,00000	0,00000
	TRGV11*01TRGJ1*02	CACWIRHVRYKKLF	0,00737	0,00000	0,00000
	TRGV3*01TRGJ1*02	CATWDGL#YYKKLF	0,00716	0,02162	0,00000
	TRGV2*02TRGJ1*02	CATWDGLNE*R#YKKLF	0,00680	0,00000	0,00000
	TRGV8*01TRGJ1*02	CATWDP#YYKKLF	0,00673	0,00000	0,00000
	TRGV8*01TRGJ1*02	CATWDIDYKKLF	0,00652	0,00000	0,00000
	TRGV2*03TRGJ2*01	CATWDGR#NYYKKLF	0,00546	0,00000	0,00000
	TRGV11*01TRGJP2*01	CACWIRQLL*EGDWIKTF	0,00447	0,00000	0,00000
	TRGV11*01TRGJ1*02	CACWIFLDYKKLF	0,00432	0,00000	0,00000
	TRGV8*01TRGJ1*02	CATWAQ#KKLF	0,00432	0,00000	0,00000
	TRGV4*01TRGJ1*02	CAT#NYYKKLF	0,00425	0,00000	0,00000
	TRGV10*02TRGJ1*02	CAAWDSLPS#YYKKLF	0,00411	0,00000	0,00000
	TRGV2*01TRGJ2*01	CATWDG#HKKLF	0,00404	0,00000	0,00000
	TRGV5*01TRGJ1*02	CATWD#*YKKLF	0,00376	0,00000	0,00000
	TRGV3*02TRGJ1*01	CATWDS#YYKKLF	0,00376	0,00000	0,00000
	TRGV10*01TRGJ1*02	CAAWGR#KLF	0,00369	0,00000	0,00000
	TRGV8*01TRGJ2*01	CATWATSP#NYYKKLF	0,00347	0,00000	0,00000

TRGV8*01TRGJ2*01	CATWDRVS#YYKKLF	0,00347	0,00000	0,00000
TRGV9*01TRGJ1*01	CALWEVW#FKKLF	0,00333	0,00000	0,00000
TRGV9*01TRGJ1*02	CALWEVQL#NYYKKLF	0,00326	0,00000	0,00000
TRGV2*03TRGJ2*01	CATWPSERG#KKLF	0,00326	0,00000	0,00000
TRGV3*02TRGJ1*01	CATWDSH#YYKKLF	0,00312	0,00000	0,00000
TRGV2*03TRGJ2*01	CATWDG#FYKKLF	0,00298	0,00000	0,00000
TRGV8*01TRGJ1*02	CATWDRG#YKKLF	0,00298	0,00000	0,00000
TRGV9*01TRGJ2*01	CALWSLKLF	0,00248	0,00000	0,00000
TRGV2*02TRGJ1*02	CATW#NYYKKLF	0,00241	0,00000	0,00000
TRGV9*01TRGJ1*02	CALWEVQPG#YKKLF	0,00234	0,00000	0,00000
TRGV5*01TRGJ1*02	CATWS#NYYKKLF	0,00149	0,01124	0,00000
TRGV10*02TRGJ1*01	CAAWELKKLF	0,00135	0,01669	0,00000
TRGV10*02TRGJ1*01	CAAWD#YYKKLF	0,00092	0,00000	0,00000
TRGV8*01TRGJP1*01	CATWAASTGWFKIF	0,00078	0,06025	0,00000
TRGV9*01TRGJ1*01	CALWEVKVG#YKKLF	0,00014	0,00000	0,00000
TRGV4*02TRGJ1*01	CATWDGR#YKKLF	0,00014	0,00000	0,00000
TRGV4*02TRGJ1*02	CATWDPPDKKLF	0,00007	0,00000	0,00000
TRGV10*02TRGJ1*01	CAACTT#YKKLF	0,00000	0,00762	0,00000
TRGV10*02TRGJ1*02	CAAWDCNYYKKLF	0,00000	0,00953	0,00000
TRGV10*02TRGJ1*01	CAAWDFNSTGDGHH#NYYKKLF	0,00000	0,01321	0,00055
TRGV10*02TRGJ1*02	CAAWV#YYKKLF	0,00000	0,00460	0,00000
TRGV11*01TRGJ2*01	CACWISL#YYKKLF	0,00000	0,02398	0,00000
TRGV9*01TRGJ1*01	CALWDQE#KKLF	0,00000	0,00565	0,00000
TRGV9*01TRGJ1*02	CALWEGPVG#YKKLF	0,00000	0,01150	0,00000
TRGV9*01TRGJ1*02	CALWEVR#YKKLF	0,00000	0,00158	0,12867
TRGV5*01TRGJ2*01	CAPPLF	0,00000	0,01005	0,00000
TRGV8*01TRGJ2*01	CATWAER#KLF	0,00000	0,01084	0,00000
TRGV4*01TRGJP2*01	CATWD*YNW#DWIKTF	0,00000	0,00000	0,09524
TRGV2*02TRGJ1*02	CATWDDP#YYKKLF	0,00000	0,00611	0,00000
TRGV3*01TRGJ1*02	CATWDGF#YYKKLF	0,00000	0,00493	0,00185
TRGV2*01TRGJ1*01	CATWDGHKKLF	0,00000	0,00696	0,00000
TRGV2*03TRGJ1*02	CATWDGHYYKKLF	0,00000	0,00473	0,00000
TRGV2*01TRGJP1*01	CATWDGPRDTTGWFKIF	0,00000	0,00414	0,07444

TRGV2*03TRGJ1*02	CATWDGPRKKLF	0,00000	0,00749	0,00000
TRGV8*01TRGJ1*01	CATWDLPGV#KLF	0,00000	0,00920	0,00000
TRGV3*02TRGJ2*01	CATWDR#KKLF	0,00000	0,00447	0,00000
TRGV8*01TRGJ2*01	CATWDRAT#YKKLF	0,00000	0,00269	0,00000
TRGV8*01TRGJ2*01	CATWDRED#KLF	0,00000	0,00427	0,00000
TRGV8*01TRGJP*01	CATWDRQELGKKIKVF	0,00000	0,00000	0,36401
TRGV5*01TRGJ1*02	CATWDRQTRLN#YYKKLF	0,00000	0,00762	0,00000
TRGV3*01TRGJ1*02	CATWDRRV#YKKLF	0,00000	0,00670	0,00000
TRGV8*01TRGJ1*02	CATWDSPHYKKLF	0,00000	0,00880	0,00000
TRGV1*01TRGJ1*02	LPPGTGRYYKKLF	0,00000	0,00000	0,00336

			Patient tumor	PDX12-P1_Spleen	PDX12-P1_Lung	PDX12-P3_Spleen	PDX12-P3_Skin
mutational status (NGS)	gene	p.AA	variant allele frequency (%)				
	RHOA	p.G17V	0,17400	0,03800	ND	0,57400	ND
	TET2	p.R1261C	0,19600	0,05200	ND	0,47400	ND
	TET2	p.R1465X	0,26600	0,05800	ND	0,43500	ND
TCRbeta repertoire	beta gene rearrangement	Junction AA	% of rearrangement withing all TCRB gene rearrangements				
	TRBV12-3*01TRBJ1-2*01	CASSQRGQGLDRGYTF	0,22385	0,27555	0,81579	0,97541	0,93190
	TRBV11-3*04TRBJ2-3*01	CASSSRTSGGRTDTQYF	0,05095	0,00000	0,00000	0,00000	0,00000
	TRBV27*01TRBJ2-7*01	CASSYVGSYEQYF	0,04543	0,00000	0,00000	0,00000	0,00000
	TRBV21-1*01TRBJ2-1*01	CASSGGYQTSGRSDEQFF	0,04275	0,00000	0,00000	0,00000	0,00000
	TRBV12-4*01TRBJ1-5*01	CASSFRDGLDQPQHF	0,03849	0,00000	0,00000	0,00000	0,00000
	TRBV11-3*03TRBJ2-2*01	CASRPGTSVLNTGELFF	0,02982	0,00000	0,00000	0,00000	0,00000
	TRBV7-2*02TRBJ1-2*01	CASSFITGNGYTF	0,02698	0,00000	0,00000	0,00000	0,00000
	TRBV5-1*01TRBJ2-6*01	CASSPNQGSVTGANVLTF	0,02619	0,00000	0,00000	0,00000	0,00000
	TRBV4-1*02TRBJ1-1*01	CASSQGQGRGNTEAFF	0,00000	0,14537	0,00000	0,00000	0,00000
	TRBV6-5*01TRBJ1-4*01	CASSLTGGNEKLFF	0,00000	0,10601	0,00000	0,00000	0,00000
	TRBV19*03TRBJ1-5*01	CASSAYTGVLSNQPQHF	0,00000	0,05525	0,00000	0,00000	0,00000
	TRBV14*01TRBJ1-1*01	CASSQDGLPAFF	0,00000	0,02072	0,00000	0,00000	0,00000
	TRBV6-6*01TRBJ1-1*01	CASSSLAGKNTEAFF	0,00000	0,01485	0,00000	0,00000	0,00000
	TRBV7-9*01TRBJ2-1*01	CASSFHRDEQFF	0,00000	0,01347	0,00000	0,00000	0,00000
	TRBV7-6*01TRBJ1-2*01	CASSSTTGPPRAGYTF	0,00000	0,01209	0,00000	0,00000	0,00000
	TRBV28*01TRBJ2-3*01	CASSRYGTVSTSTDTQYF	0,00000	0,00000	0,13123	0,00000	0,00000
	TRBV10-2*01TRBJ2-2*01	CASIGTVNTGELFF	0,00000	0,00000	0,00213	0,00000	0,00000
TCRalpha repertoire	alpha gene rearrangement	Junction AA (around half non coding)	% of rearrangement withing all TCRA gene rearrangements				
	TRAV8-6*01TRAJ48*01	CAVTVNFGNEKLTF	0,15949	0,25409	0,36445	0,47731	0,80084
	TRAV20*01TRAJ40*01	CAVC#GTYKYIF	0,12590	0,21736	0,32807	0,51362	0,19916
	TRAV12-3*01TRAJ54*01	CAMSAA#GAQKLVF	0,01840	0,00000	0,00000	0,00000	0,00000
	TRAV12-3*01TRAJ39*01	CAMSDNAGNMLTF	0,01813	0,00000	0,00000	0,00000	0,00000
	TRAV21*01TRAJ28*01	CAVPRGAGSYQLTF	0,01520	0,00000	0,00000	0,00000	0,00000
	TRAV23/DV6*01TRAJ48*01	CAARGC#FGNEKLTF	0,01435	0,00000	0,00000	0,00000	0,00000
	TRAV27*01TRAJ37*01	CAFSGNTGKLIF	0,01402	0,00000	0,00000	0,00000	0,00000
	TRAV12-2*02TRAJ28*01	CAV*GS	0,01370	0,00000	0,00000	0,00000	0,00000
	TRAV19*01TRAJ40*01	CALRASGTYKYIF	0,01220	0,00000	0,00000	0,00000	0,00000

	TRAV26-2*01TRAJ31*01	CILRDVDNARLMF	0,01168	0,00000	0,00000	0,00000	0,00000
	TRAV38-1*01TRAJ48*01	CAFMKH#GNEKLTF	0,01155	0,00000	0,00000	0,00000	0,00000
	TRAV13-2*01TRAJ7*01	CAENFYGNNRLAF	0,01129	0,00000	0,00000	0,00000	0,00000
	TRAV13-1*01TRAJ36*01	CAASINDGANNLFF	0,01063	0,00000	0,00000	0,00000	0,00000
	TRAV9-2*01TRAJ21*01	CALANFNKFYF	0,01037	0,00000	0,00000	0,00000	0,00000
	TRAV35*01TRAJ57*01	CAGP*HRLTQGGSEKLVF	0,01011	0,00000	0,00000	0,00000	0,00000
	TRAV38-2/DV8*01TRAJ33*01	CALGEHTSNYQLIW	0,00978	0,00000	0,00000	0,00000	0,00000
	TRAV12-2*02TRAJ13*01	CAVPVFLGGYQKVTF	0,00952	0,00000	0,00000	0,00000	0,00000
	TRAV13-1*01TRAJ11*01	CAALLIASGYSTLTF	0,00000	0,24820	0,16673	0,00000	0,00000
	TRAV8-1*01TRAJ44*01	CAVRTAY#NTGTASKLTF	0,00000	0,22462	0,09354	0,00000	0,00000
	TRAV9-2*01TRAJ3*01	C#SSASKIIF	0,00000	0,01040	0,00000	0,00000	0,00000
	TRAV3*01TRAJ44*01	CAVRTSY#NTGTASKLTF	0,00000	0,00853	0,00000	0,00000	0,00000
	TRAV2*02TRAJ20*01	CAVSLG#NDYKLSF	0,00000	0,00691	0,00000	0,00000	0,00000
	TRAV12-3*01TRAJ53*01	CAMS#*GSNYKLTF	0,00000	0,00441	0,00511	0,00000	0,00000
	TRAV24*01TRAJ48*01	CAF#NFGNEKLTF	0,00000	0,00271	0,01259	0,00000	0,00000
	TRAV20*04TRAJ40*01	CAMC#GTYKYIF	0,00000		0,00568	0,00000	0,00000
TCRgamma repertoire	**gamma gene rearrangement**	**Junction AA (Mostly non coding)**	**% of rearrangement withing all TCRG gene rearrangements**				
	**TRGV4*02TRGJ1*02**	**CAAP#KKLF**	**0,14393**	**0,15124**	**0,30127**	**0,21527**	**0,45032**
	**TRGV5*01TRGJ1*02**	**CATWDRLR#YYKKLF**	**0,13490**	**0,21161**	**0,39499**	**0,63438**	**0,24313**
	TRGV10*02TRGJ2*01	CAAWD*R#YYKKLF	0,02067	0,00000	0,00000	0,00000	0,00000
	TRGV8*01TRGJ1*01	CATWD#KKLF	0,01980	0,00000	0,00000	0,00000	0,00000
	TRGV2*01TRGJ1*02	CATWDL#KLF	0,01933	0,00000	0,00000	0,00000	0,00000
	TRGV10*02TRGJ1*01	CAAWDYNP#NYYKKLF	0,01909	0,00000	0,00000	0,00000	0,00000
	TRGV5*01TRGJ1*02	CATWDRRV#YYKKLF	0,01838	0,00000	0,00000	0,00000	0,00000
	TRGV10*02TRGJ1*02	CAAW#YYKKLF	0,01782	0,00000	0,00000	0,00000	0,00000
	TRGV10*02TRGJ1*02	CAAWD#YYKKLF	0,01719	0,00000	0,00000	0,00000	0,00000
	TRGV10*01TRGJ1*02	CAAGSI*G#YKKLF	0,01719	0,00000	0,00000	0,00000	0,00000
	TRGV8*01TRGJ2*01	CATWDSSKNYYKKLF	0,01711	0,00000	0,00000	0,00000	0,00000
	TRGV9*01TRGJP*01	CALWEV#ELGKKIKVF	0,01608	0,00000	0,00000	0,00000	0,00000
	TRGV4*01TRGJ1*02	CATF#NYYKKLF	0,01513	0,00000	0,00000	0,00000	0,00000
	TRGV5P*01TRGJ2*01	CATWAR#YYKKLF	0,01481	0,00000	0,00000	0,00000	0,00000
	TRGV10*02TRGJ1*02	CAAWDYYKKLF	0,01465	0,00000	0,00000	0,00000	0,00000

TRGV10*02TRGJ1*02	CAAWD#NYYKKLF	0,01442	0,00000	0,00000	0,00000	0,00000
TRGV3*02TRGJ1*02	CATWESKEKLF	0,01434	0,00000	0,00000	0,00000	0,00000
TRGV10*02TRGJ1*01	CAAWD*#YKKLF	0,01291	0,00000	0,00000	0,00000	0,00000
TRGV10*02TRGJ1*01	CAA*GGDKKLF	0,01267	0,00000	0,00000	0,00000	0,00000
TRGV10*02TRGJP1*01	CAAWDYR#TGWFKIF	0,01164	0,00000	0,00000	0,00000	0,00000
TRGV4*01TRGJ2*01	CATWD#SYKKLF	0,01157	0,00000	0,00000	0,00000	0,00000
TRGV4*01TRGJ2*01	CATWDV#YYKKLF	0,00840	0,00000	0,00000	0,00000	0,00000
TRGV5*01TRGJ1*02	CATWNLPR#KKLF	0,00000	0,26830	0,15770	0,00000	0,00000
TRGV10*02TRGJP1*01	CAAWGPS#TGWFKIF	0,00000	0,25568	0,10125	0,00000	0,00000
TRGV10*02TRGJ1*01	CAAWDPR*IYYKKLF	0,00000	0,02961	0,00630	0,00000	0,00000
TRGV8*01TRGJ1*02	CATWDS#YKKLF	0,00000	0,01843	0,00000	0,00000	0,00000
TRGV4*01TRGJ1*02	CATWGYIG#YYKKLF	0,00000	0,01640	0,00203	0,00000	0,00000
TRGV5*01TRGJ1*02	CATWDRQ#YYKKLF	0,00000	0,01334	0,00000	0,00000	0,00000
TRGV1*01TRGJ1*02	CATWDRLR#YYKKLF	0,00000	0,00106	0,00392	0,13780	0,28541

mutational status (NGS)	gene	p.AA	Patient tumor	PDX13-P1_Spleen	PDX13-P2_Spleen	PDX13-P2_ spleen CD4+ T cells	PDX13-P2_ spleen CD8+ T cells
			variant allele frequency (%)				
	CD28	p.D124E	0,16400	0,00000	0,00000	0,00000	0,00000
	RHOA	p.G17V	0,24800	0,06700	0,42300	0,47900	0,00000
	TET2	c.3955-2A>G	0,25200	0,04400	0,41600	0,53400	0,00000
	TET2	c.3803+2T>-	0,45900	0,48400	0,52200	0,48600	0,50900
	DNMT3A	p.N612fs	0,20800	0,44000	0,46000	0,47900	0,48500

TCRbeta repertoire	beta gene rearrangement	Junction AA	% of rearrangement withing all TCRB gene rearrangements				
	TRBV10-1*01TRBJ2-3*01	CASSEQGAGDTQYF	0,64941	0,05764	0,66563	0,88689	0,00051
	TRBV11-2*01TRBJ2-7*01	CASSYAGSYEQYF	0,01106	0,00000	0,00000	0,00000	0,00000
	TRBV6-5*01TRBJ2-7*01	CASSWTG*#SYEQYF	0,00796	0,00000	0,00000	0,00000	0,00000
	TRBV5-3*01TRBJ2-1*01	CARSSYRKGGSYNEQFF	0,00664	0,00000	0,00000	0,00000	0,00000
	TRBV25-1*01TRBJ2-7*01	CASSDMPGAYEQYF	0,00619	0,00000	0,00000	0,00000	0,00000
	TRBV6-5*01TRBJ2-7*01	CASSYSGTGGLYEQYF	0,00575	0,00000	0,00000	0,00000	0,00000
	TRBV3-1*01TRBJ2-7*01	CASSQDEGAYEQYF	0,00575	0,00000	0,00000	0,00000	0,00000
	TRBV21-1*01TRBJ2-5*01	CASSTRQGH*ETQYF	0,00575	0,00000	0,00000	0,00000	0,00000
	TRBV19*03TRBJ2-7*01	CASRTGDSFSYEQYF	0,00553	0,00000	0,00000	0,00000	0,00000
	TRBV29-1*01TRBJ1-2*01	CSVEGIYGYTF	0,00000	0,35733	0,04636	0,00000	0,00040
	TRBV27*01TRBJ1-2*01	CASTTPPLGGLVGYTF	0,00000	0,04425	0,03796	0,00000	0,79060
	TRBV12-4*01TRBJ2-2*01	CASSYRW#NTGELFF	0,00000	0,26465	0,03758	0,00057	0,00071
	TRBV28*01TRBJ2-7*01	CASRLSGEIHEQYF	0,00000	0,00582	0,07979	0,00014	0,00243
	TRBV4-1*02TRBJ2-7*01	CASSLLTPLAGAAPGSEQYF	0,00000	0,08695	0,00331	0,00000	0,05149
	TRBV28*01TRBJ2-1*01	CASRSQHGSSYNEQFF	0,00000	0,00017	0,00321	0,00000	0,04299
	TRBV6-3*01TRBJ1-2*01	CASSYDSYYGYTF	0,00000	0,01210	0,00000	0,00000	0,00000
	TRBV3-2*03TRBJ2-6*01	CASSPKTGS*#SGANVLTF	0,00000	0,01030	0,00000	0,00000	0,00000
	TRBV6-3*01TRBJ2-3*01	CASSYSGLAGVSDTQYF	0,00000	0,00523	0,00000	0,00000	0,00000
	TRBV4-1*01TRBJ2-7*01	CASSQELVPGGYSSYEQYF	0,00000	0,00498	0,00000	0,00000	0,00000

	TRBV7-2*01TRBJ2-1*01	CASSLARLAGSSYNEQFF	0,00000	0,00205	0,00000	0,00000	0,00000
**TCRalpha repertoire**	**alpha gene rearrangement**	**Junction AA (around half non coding)**	**% of rearrangement withing all TCRA gene rearrangements**				
	**TRAV12-3*01TRAJ28*01**	**CAGL#GAGSYQLTFGKGTKLSVIPSKFFFLANYST**	**0,47114**	**0,05897**	**0,32407**	**0,38430**	**0,00117**
	**TRAV8-6*01TRAJ42*01**	**CAVSEGSQGNLIF**	**0,32353**	**0,08033**	**0,46712**	**0,52203**	**0,00039**
	TRAV8-3*01TRAJ6*01	CAVGAVGGSYIPTF	0,00206	0,00000	0,00000	0,00000	0,00000
	TRAV12-2*03TRAJ13*02	CAVSGGYQKVTF	0,00206	0,00000	0,00000	0,00000	0,00000
	TRAV29/DV5*03TRAJ61*01	CAASAT*V#RVNRKLTF	0,00190	0,00000	0,00000	0,00000	0,00000
	TRAV2*01TRAJ40*01	CAVRGSGTYKYIF	0,00185	0,00000	0,00000	0,00000	0,00000
	TRAV13-1*01TRAJ44*01	CAASIRVE#TGTASKLTF	0,00185	0,00000	0,00000	0,00000	0,00000
	TRAV22*01TRAJ39*01	WV#NNAGNMLTF	0,00185	0,00000	0,00000	0,00000	0,00000
	TRAV26-2*01TRAJ56*01	CIG#TGANSKLTF	0,00180	0,00000	0,00000	0,00000	0,00000
	TRAV1-2*01TRAJ20*01	CAVNDYKLSF	0,00175	0,00000	0,00000	0,00000	0,00000
	TRAV10*01TRAJ43*01	CVVSAKSNNDMRF	0,00159	0,00000	0,00000	0,00000	0,00000
	TRAV13-2*02TRAJ53*01	CAEIWDSGGSNYKLTF	0,00159	0,00000	0,00000	0,00000	0,00000
	TRAV16*01TRAJ43*01	CDNNNDMRF	0,00159	0,00000	0,00000	0,00000	0,00000
	TRAV21*01TRAJ23*01	CAVR#NQGGKLIF	0,00159	0,00000	0,00000	0,00000	0,00000
	TRAV35*01TRAJ57*01	CAGPLTQGGSEKLVF	0,00139	0,00000	0,00000	0,00000	0,00000
	TRAV8-4*03TRAJ41*01	CAVTNSNSGYALNF	0,00139	0,00000	0,00000	0,00000	0,00000
	TRAV24*01TRAJ45*01	CAF#RRRC*RTHLWQRDSS	0,00134	0,00000	0,00000	0,00000	0,00000
	TRAV2*01TRAJ26*01	CAAYNYGQNFVF	0,00134	0,00000	0,00000	0,00000	0,00000
	TRAV29/DV5*03TRAJ49*01	AANVPP#NTGNQFYF	0,00134	0,00000	0,00000	0,00000	0,00000
	TRAV19*01TRAJ39*01	CALSEASRRGNNAGNMLTF	0,00128	0,00000	0,00000	0,00000	0,00000
	TRAV14/DV4*01TRAJ52*01	CAPRP#GGTSYGKLTF	0,00000	0,21768	0,01139	0,00058	0,00000
	TRAV9-2*01TRAJ49*01	CALSDPTGNQFYF	0,00000	0,17864	0,00886	0,00000	0,00031
	TRAV38-2/DV8*01TRAJ53*01	CAYRVTYSGGSNYKLTF	0,00000	0,10733	0,00257	0,00000	0,03491
	TRAV19*01TRAJ54*01	CAP#TQGAQKLVF	0,00000	0,08060	0,00092	0,00000	0,02799
	TRAV12-1*01TRAJ10*01	CVVNEG#TGGGNKLTF	0,00000	0,05252	0,01797	0,00000	0,47301
	TRAV4*01TRAJ36*01	CLVGDRTGANNLFF	0,00000	0,03268	0,01422	0,00006	0,30804
	TRAV39*01TRAJ42*01	CPDYGGSQGNLIF	0,00000	0,00537	0,00000	0,00000	0,00000
	TRAV26-1*01TRAJ48*01	CIVRL#FGNEKLTF	0,00000	0,00526	0,00000	0,00000	0,00000
	TRAV8-3*01TRAJ13*02	CAVGA#NSGGYQKVTF	0,00000	0,00459	0,02468	0,00014	0,00074

TRAV19*01TRAJ5*01	CALSGE#DTGRRALTF	0,00000	0,00429	0,00000	0,00000	0,00000
TRAV17*01TRAJ8*01	CATAPEVVVNTGFQKLVF	0,00000	0,00376	0,00000	0,00000	0,00000
TRAV35*01TRAJ40*01	CAGQSLWSGTYKYIF	0,00000	0,00367	0,00000	0,00000	0,00000
TRAV13-2*02TRAJ8*01	CAERSGFQKLVF	0,00000	0,00351	0,01628	0,00016	0,00076
TRAV22*01TRAJ31*01	CAVGRNNNARLMF	0,00000	0,00302	0,00000	0,00000	0,00000
TRAV8-3*01TRAJ10*01	CAVG#STGGGNKLTF	0,00000	0,00273	0,00000	0,00000	0,00000
TRAV29/DV5*03TRAJ43*01	CAGQH#NDMRF	0,00000	0,00208	0,00000	0,00000	0,00000
TRAV12-3*01TRAJ52*01	CAMSAYAGGTSYGKLTF	0,00000	0,00139	0,00000	0,00000	0,00000
TRAV3*02TRAJ52*01	CAPRP#GGTSYGKLTF	0,00000	0,00114	0,00022	0,00000	0,00000
TRAV8-6*01TRAJ13*02	CAGGGYQKVTF	0,00000	0,00031	0,00112	0,00000	0,01920
TRAV4*01TRAJ27*01	CLVGEEPL#NTNAGKSTF	0,00000	0,00000	0,00043	0,00000	0,01713

TCRgamma repertoire

gamma gene rearrangement	Junction AA (Mostly non coding)	% of rearrangement withing all TCRG gene rearrangements				
**TRGV8*01TRGJ1*02**	**CATWDRRHYKKLF**	**0,41336**	**0,07188**	**0,44245**	**0,49812**	**0,00124**
**TRGV7*01TRGJ1*02**	**VPPGTGAFQSG#KLF**	**0,34887**	**0,06649**	**0,33358**	**0,40421**	**0,00135**
TRGV9*01TRGJ1*02	CAL#IKKLF	0,00428	0,00000	0,00000	0,00000	0,00000
TRGV10*02TRGJ1*01	CAAWD#NYYKKLF	0,00379	0,00000	0,00000	0,00000	0,00000
TRGV2*01TRGJ1*02	CATWDGR#YKKLF	0,00316	0,00000	0,00000	0,00000	0,00000
TRGV10*02TRGJP1*01	not determined	0,00288	0,00000	0,00000	0,00000	0,00000
TRGV9*01TRGJ2*01	CALWEV#NYYKKLF	0,00267	0,00000	0,00000	0,00000	0,00000
TRGV11*01TRGJ2*01	CACWIRHR#YKKLF	0,00246	0,00000	0,00000	0,00000	0,00000
TRGV10*02TRGJ2*01	CAAWD*NHKKLF	0,00232	0,00000	0,00000	0,00000	0,00000
TRGV10*02TRGJP1*01	CAAWATYNATGWFKIF	0,00225	0,00000	0,00000	0,00000	0,00000
TRGV10*02TRGJP1*01	CAAWDSACKFKIF	0,00218	0,00000	0,00000	0,00000	0,00000
TRGV2*02TRGJ1*02	CATWIE#YKKLF	0,00218	0,00000	0,00000	0,00000	0,00000
TRGV5*01TRGJ2*01	CATWDGQRYKKLF	0,00211	0,00000	0,00000	0,00000	0,00000
TRGV2*01TRGJ2*01	CATWDR#YKKLF	0,00211	0,00000	0,00000	0,00000	0,00000
TRGV4*01TRGJ1*01	CATWEE#YKKLF	0,00211	0,00000	0,00000	0,00000	0,00000
TRGV4*01TRGJ1*02	CATWVLLPLWR#KLF	0,00211	0,00000	0,00000	0,00000	0,00000
TRGV4*01TRGJ1*02	CAPS#KLF	0,00197	0,00000	0,00000	0,00000	0,00000
TRGV5*01TRGJ1*02	CATWDGTNYYKKLF	0,00197	0,00000	0,00000	0,00000	0,00000
TRGV10*02TRGJ1*01	CAAWDLWVYKKLF	0,00189	0,00000	0,00000	0,00000	0,00000
TRGV2*01TRGJP1*01	CATWDGHI#TTGWFKIF	0,00189	0,00000	0,00000	0,00000	0,00000

TRGV8*01TRGJP1*01	CATWDR#GWFKIF	0,00189	0,00000	0,00000	0,00000	0,00000
TRGV11*01TRGJ2*01	CACWI#SEKLF	0,00182	0,00000	0,00000	0,00000	0,00000
TRGV4*01TRGJ1*02	CATWDG#NYKKLF	0,00182	0,00000	0,00000	0,00000	0,00000
TRGV3*02TRGJ2*01	CATWDSCWLF#LF	0,00182	0,00000	0,00000	0,00000	0,00000
TRGV5*01TRGJ1*02	CATWDRHVFYKKLF	0,00175	0,00000	0,00000	0,00000	0,00000
TRGV1*01TRGJ2*01	not determined	0,00021	0,00002	0,00086	0,00068	0,00000
TRGV10*02TRGJ1*02	CAAWASSPLSGKLF	0,00000	0,00258	0,00000	0,00000	0,00000
TRGV10*02TRGJ1*02	CAAWD*#KLF	0,00000	0,00214	0,00000	0,00000	0,00000
TRGV10*02TRGJP1*01	CAAWGLL#TTGWFKIF	0,00000	0,13699	0,00823	0,00000	0,00023
TRGV9*01TRGJ1*01	CALESRYKKLF	0,00000	0,11271	0,00179	0,00000	0,04225
TRGV9*01TRGJ1*02	CALWEA#NYYKKLF	0,00000	0,24236	0,01517	0,00014	0,00100
TRGV9*01TRGJ1*02	CALWEL#YYKKLF	0,00000	0,00012	0,00010	0,00000	0,00157
TRGV9*01TRGJ1*02	CALWEV#YYKKLF	0,00000	0,04991	0,02200	0,00000	0,49050
TRGV9*01TRGJ1*02	CALWEV#YYKKLF	0,00000	0,00025	0,00023	0,00000	0,00297
TRGV2*01TRGJP2*01	CAT#SSDWIKTF	0,00000	0,00322	0,01593	0,00018	0,00124
TRGV8*01TRGJ1*02	CATF#YKKLF	0,00000	0,00023	0,00086	0,00000	0,01979
TRGV2*02TRGJ1*01	CATWD#FYKKLF	0,00000	0,00268	0,00000	0,00000	0,00000
TRGV2*02TRGJP1*01	CATWDFVS#PGWFKIF	0,00000	0,00468	0,00000	0,00000	0,00000
TRGV2*01TRGJ1*02	CATWDGP#NYYKKLF	0,00000	0,03108	0,01371	0,00000	0,24833
TRGV2*01TRGJ1*01	CATWDGRRYYKKLF	0,00000	0,00101	0,00000	0,00000	0,00000
TRGV2*01TRGJP2*01	CATWDGTYPSDWIKTF	0,00000	0,00375	0,00000	0,00000	0,00000
TRGV4*01TRGJ1*02	CATWDGWL#NYYKKLF	0,00000	0,00000	0,00066	0,00000	0,01552
TRGV3*01TRGJP2*01	CATWDRPGVRSDWIKTF	0,00000	0,00155	0,00000	0,00000	0,00000
TRGV3*02TRGJ2*01	CATWDS#YYKKLF	0,00000	0,00157	0,00000	0,00000	0,00000
TRGV2*02TRGJ1*02	CATWDSPNYYKKLF	0,00000	0,00340	0,00000	0,00000	0,00000
TRGV8*01TRGJ1*02	CATWDSPQP#YYKKLF	0,00000	0,00683	0,02990	0,00012	0,00154
TRGV2*01TRGJP2*01	CATWE#SSDWIKTF	0,00000	0,08291	0,00062	0,00000	0,02964
TRGV4*01TRGJ2*01	CATWEG#LYKKLF	0,00000	0,00367	0,00000	0,00000	0,00000

			Patient tumor	PDX16-P1_Spleen	PDX16-P3_Spleen
**mutational status (NGS)**	**gene**	**p.AA**	**variant allele frequency (%)**		
	RHOA	p.G17V	0,23600	0,01600	0,00000
	TET2	p.G1861R	0,37300	0,50400	0,51100
	TET2	p.H1380Y	0,38900	0,43200	0,43600
	DNMT3A	p.R882H	0,38500	0,49700	0,43800
**TCRbeta repertoire**	**beta gene rearrangement**	**Junction AA**	**% of rearrangement withing all TCRB gene rearrangements**		
	TRBV5-6*01TRBJ2-5*01	CASSFGGIETQYF	0,14916	0,00000	0,00000
	TRBV6-1*01TRBJ1-1*01	CASRVATEAFF	0,11840	0,00000	0,00000
	TRBV27*01TRBJ2-3*01	CASSLATGGGEGSTDTQYF	0,10157	0,00000	0,00000
	TRBV19*01TRBJ1-5*01	CASSIAGTSNQPQHF	0,08706	0,00000	0,00000
	TRBV6-3*01TRBJ2-7*01	CASNILTW#SYEQYF	0,08474	0,00000	0,00000
	TRBV28*01TRBJ1-1*01	CASSLLRATEAFF	0,00000	0,29618	0,57311
	TRBV28*01TRBJ1-4*01	CASRFDRGLSNEKLFF	0,00000	0,03493	0,27052
	TRBV9*01TRBJ1-4*01	CASSVGGQDGEKLFF	0,00000	0,01800	0,06534
	TRBV21-1*01TRBJ1-1*01	CASSKALGRNTEAFF	0,00000	0,00056	0,06474
	TRBV3-1*01TRBJ2-1*01	CASSPPGRITYNEQFF	0,00000	0,13518	0,00000
	TRBV12-4*01TRBJ2-4*01	CASSESRGTLSQYF	0,00000	0,05373	0,00000
	TRBV28*01TRBJ2-7*01	CASSLSLGRLGEQYF	0,00000	0,04827	0,00000
	TRBV13*01TRBJ1-1*01	CASSSQPHTEAFF	0,00000	0,04755	0,00000
	TRBV6-5*01TRBJ1-4*01	CASTGGEKLFF	0,00000	0,04599	0,00000
	TRBV19*03TRBJ2-3*01	CASRKAGTVTDTQYF	0,00000	0,03409	0,00000
	TRBV5-1*01TRBJ2-5*01	CASSLLLTGYSQETQYF	0,00000	0,02902	0,00000
	TRBV4-2*01TRBJ1-4*01	CASSQDEGAVNEKLFF	0,00000	0,02303	0,00000
	TRBV21-1*01TRBJ1-2*01	CASSKYYRANYGYTF	0,00000	0,01892	0,00000
	TRBV10-1*01TRBJ2-3*01	CASSEQGAGDTQYF	0,00000	0,01369	0,00000
**TCRalpha repertoire**	**alpha gene rearrangement**	**Junction AA (around half non coding)**	**% of rearrangement withing all TCRA gene rearrangements**		

TRAV9-2*03TRAJ10*01	CAIRTGGGNKLTF	0,61019	0,00000	0,00000
TRAV8-2*02TRAJ42*01	CVVQGGSQGNLIF	0,03269	0,00000	0,00000
TRAV8-6*01TRAJ32*02	CAVSPYGGATNKLIF	0,02918	0,00000	0,00000
TRAV3*01TRAJ15*01	CAVREW#QAGTALIF	0,02083	0,00000	0,00000
TRAV4*01TRAJ26*01	CLVGHPL#NYGQNFVF	0,01857	0,00000	0,00000
TRAV17*01TRAJ43*01	CATATYNNNDMRF	0,01694	0,00000	0,00000
TRAV39*01TRAJ58*01	CAVEGIKW#ETSGSRLTF	0,01493	0,00000	0,00000
TRAV23/DV6*01TRAJ54*01	CAPQGAQKLVF	0,01399	0,00000	0,00000
TRAV9-2*03TRAJ52*01	CALNAGGTSYGKLTF	0,01337	0,00000	0,00000
TRAV21*01TRAJ29*01	CAVWYR#RSGNTPLVF	0,01305	0,00000	0,00000
TRAV2*01TRAJ24*02	CAALTDSWGKLQF	0,01217	0,00000	0,00000
TRAV12-1*01TRAJ40*01	CAVKG#SGTYKYIF	0,01130	0,00000	0,00000
TRAV9-2*01TRAJ41*01	CALSFSNSGYALNF	0,01048	0,00000	0,00000
TRAV35*01TRAJ52*01	CAGQTGTSYGKLTF	0,00879	0,00000	0,00000
TRAV26-1*01TRAJ37*01	CIV#GNTGKLIF	0,00879	0,00000	0,00000
TRAV6*01TRAJ11*01	CALGANSGYSTLTF	0,00853	0,00000	0,00000
TRAV39*01TRAJ42*01	CAPRYYGGSQGNLIF	0,00000	0,20905	0,39335
TRAV9-2*01TRAJ39*01	CALSYNAGNMLTF	0,00000	0,06428	0,00000
TRAV8-1*01TRAJ39*01	CAVLNNAGNMLTF	0,00000	0,05914	0,00000
TRAV12-1*01TRAJ17*01	CVVNINISG*#AAGNKLTF	0,00000	0,05399	0,00000
TRAV12-3*01TRAJ38*01	CA*#CWQQP*ADL	0,00000	0,04254	0,00000
TRAV9-2*01TRAJ43*01	CALRRLNDMRF	0,00000	0,04222	0,00000
TRAV22*01TRAJ50*01	CAV#KTSYDKVIF	0,00000	0,03845	0,00000
TRAV17*01TRAJ18*01	CATDARRGSTLGRLYF	0,00000	0,03649	0,00000
TRAV8-6*01TRAJ52*01	CAVNAGGTSYGKLTF	0,00000	0,03272	0,00000
TRAV27*01TRAJ31*01	CAGAHP#NNARLMF	0,00000	0,03204	0,00000

	TRAV12-2*02TRAJ20*01	CAVENDYKLSF	0,00000	0,02808	0,00000
	TRAV1-2*01TRAJ6*01	CAVAS#GGSYIPTF	0,00000	0,02151	0,20713
	TRAV36/DV7*05TRAJ45*01	CAVG#DS	0,00000	0,01567	0,00000
	TRAV30*03TRAJ27*01	CGIYNTNAGKSTF	0,00000	0,01467	0,00000
	TRAV12-2*02TRAJ5*01	CAVCMDTGRRALTF	0,00000	0,01409	0,01789
	TRAV4*01TRAJ53*01	CLVGA#NSGGSNYKLTF	0,00000	0,01319	0,00000
	TRAV1-1*01TRAJ36*01	CAGL#NGANNLFF	0,00000	0,01293	0,00000
	TRAV13-1*01TRAJ3*01	CAAKGYSSASKIIF	0,00000	0,00955	0,35307
	TRAV1-1*02TRAJ26*01	CAVFRGNYGQNFVF	0,00000	0,00665	0,00856
TCRgamma repertoire	**gamma gene rearrangement**	**Junction AA (Mostly non coding)**	**% of rearrangement withing all TCRG gene rearrangements**		
	**TRGV4*02TRGJ1*02**	**CATWV#YKKLF**	**0,78457**	**0,00081**	**0,00000**
	TRGV8*01TRGJ2*01	CATWDRW#YKKLF	0,02013	0,00000	0,00000
	TRGV8*01TRGJ2*01	CATWDNYYKKLF	0,01566	0,00000	0,00000
	TRGV2*03TRGJ1*01	CATWD*R#YYKKLF	0,01561	0,00000	0,00000
	TRGV8*01TRGJ1*01	CATWI#RKKLF	0,01409	0,00000	0,00000
	TRGV9*01TRGJ1*01	CALIH#KKLF	0,01351	0,00000	0,00000
	TRGV4*02TRGJ1*01	CATWDLCLLKLF	0,01090	0,00000	0,00000
	TRGV3*02TRGJ1*02	CATWD#NYYKKLF	0,01047	0,00000	0,00000
	TRGV4*01TRGJ1*02	CATWDGRNYYKKLF	0,00933	0,00000	0,00000
	TRGV2*03TRGJ2*01	CANKKLF	0,00909	0,00000	0,00000
	TRGV2*03TRGJ1*02	CATWDGHYKKLF	0,00799	0,00000	0,00000
	TRGV4*01TRGJ2*01	CATWDGG#YKKLF	0,00780	0,00043	0,00000
	TRGV3*02TRGJ1*02	CATCE#KKLF	0,00757	0,00000	0,00000
	TRGV8*01TRGJ2*01	CATWDS#YYKKLF	0,00562	0,00000	0,00000
	TRGV2*01TRGJ2*01	CATWDGPGRYKKLF	0,00547	0,00000	0,00000
	TRGV2*03TRGJ1*02	CATWDPG#YKKLF	0,00000	0,18061	0,26090
	TRGV7*01TRGJ1*02	not found (uncoding)	0,00000	0,10810	0,16331
	TRGV11*01TRGJ1*02	CACWIRHVRYYKKLF	0,00000	0,06872	0,00000

TRGV11*01TRGJ1*02	CACWIRHARVWGG#YYKKLF	0,00000	0,05409	0,00000
TRGV8*01TRGJ1*02	CATWPRG#YKKLF	0,00000	0,04844	0,00000
TRGV4*02TRGJ1*02	CAT*TF	0,00000	0,03529	0,20445
TRGV10*02TRGJ1*02	CAA*#YKKLF	0,00000	0,03038	0,00000
TRGV1*01TRGJ1*02	CATWDRR#YYKKLF	0,00000	0,02078	0,06002
TRGV4*01TRGJ1*02	CATWDGNNYYKKLF	0,00000	0,01945	0,00000
TRGV9*01TRGJ1*02	CALFPQLYYKKLF	0,00000	0,01788	0,00000
TRGV4*02TRGJ1*02	CATWDGP#SKKLF	0,00000	0,01764	0,00000
TRGV2*03TRGJ2*01	CATWDRD#NYYKKLF	0,00000	0,01505	0,00000
TRGV2*03TRGJ2*01	CATWDG*#YKKLF	0,00000	0,01479	0,00000
TRGV2*03TRGJ1*01	CATWDGKSNYYKKLF	0,00000	0,01454	0,00000
TRGV10*02TRGJ1*02	CAAWDYFYKKLF	0,00000	0,01326	0,00000
TRGV2*03TRGJ1*02	CATLIGKLF	0,00000	0,01227	0,00000
TRGV4*02TRGJ2*01	CATWK#YKKLF	0,00000	0,01072	0,00000
TRGV2*03TRGJP2*01	CATWDGLL#DWIKTF	0,00000	0,01070	0,00000
TRGV2*03TRGJ1*02	CATWDGP#YYKKLF	0,00000	0,00731	0,01975
TRGV3*01TRGJP2*01	CATWDRPSSDWIKTF	0,00000	0,00633	0,19566
TRGV4*01TRGJ1*02	CAPT#KKLF	0,00000	0,00155	0,02005

	gene	p.AA	Patient tumor	PDX18-P1 Spleen	PDX18-P3 Spleen	Patient Relaps	PDX18R-P1 Spleen	PDX18R-P1 Lung	PDX18R-P1 Skin	PDX18R-P2 Spleen	PDX18R-P2 Lung	PDX18R-P2 Skin	PDX18R-P3 Spleen
**mutational status (NGS)**	**gene**	**p.AA**	**variant allele frequency (%)**										
	VAV1	p.Asp161_Glu175delinsVal	0,01500	0,05300	0,14000	0,08400	0,25000	ND	ND	0,44000	ND	ND	ND
	TET2	p.E1162fs	0,08600	0,14300	0,49000	0,18800	0,52500	ND	ND	0,44200	ND	ND	ND
	TET2	c.4045-2A>-	0,08700	0,19200	0,47900	0,16800	0,45000	ND	ND	0,49200	ND	ND	ND
**TCRbeta repertoire**	**beta gene rearrangement**	**Junction AA**	**% of rearrangement withing all TCRB gene rearrangements**										
	TRBV14*01TRBJ2-7*01	CASSHGQGSYEQYF	0,01217	0,18825	0,22704	0,29859	0,47979	0,44148	0,01104	0,71681	0,73461	0,43426	0,88352
	**TRBV23-1*01TRBJ1-6*02**	**CASSQGASYSPLHF**	**0,05208**	**0,00000**	**0,00000**	**0,00000**	**0,00000**	**0,00000**	**0,00000**	**0,00000**	**0,00000**	**0,00000**	**0,00000**
	TRBV6-3*01TRBJ1-1*01	CASAQSGGNTEAFF	0,04454	0,00000	0,00000	0,00000	0,00000	0,00000	0,00000	0,00000	0,00000	0,00000	**0,00000**
	TRBV6-3*01TRBJ1-2*01	CASTPGDVSGYTF	0,04454	0,00000	0,00000	0,00000	0,00000	0,00000	0,00000	0,00000	0,00000	0,00000	**0,00000**
	TRBV11-3*04TRBJ1-2*01	CASSLPTRRGQYGYTF	0,04116	0,00000	0,00000	0,00000	0,00000	0,00000	0,00000	0,00000	0,00000	0,00000	**0,00000**
	TRBV4-1*02TRBJ2-7*01	CASSPRDLAGEQYF	0,04077	0,00000	0,00000	0,00000	0,00000	0,00000	0,00000	0,00000	0,00000	0,00000	**0,00000**
	TRBV28*01TRBJ2-7*01	CASSLARGDRLPHEQYF	0,03865	0,00000	0,00000	0,00000	0,00000	0,00000	0,00000	0,00000	0,00000	0,00000	**0,00000**
	TRBV4-2*01TRBJ1-2*01	CASSQVWEVLG#IGYTF	0,03614	0,00000	0,00000	0,00000	0,00000	0,00000	0,00000	0,00000	0,00000	0,00000	**0,00000**
	TRBV14*01TRBJ1-2*01	CASSQFRGYGYTF	0,03440	0,00000	0,00000	0,00000	0,00000	0,00000	0,00000	0,00000	0,00000	0,00000	**0,00000**
	TRBV5-6*01TRBJ2-2*01	CASS*QGAGGELFF	0,03227	0,00000	0,00000	0,00000	0,00000	0,00000	0,00000	0,00000	0,00000	0,00000	**0,00000**
	TRBV6-4*01TRBJ1-1*01	CASSDGRLNTEAFF	0,03159	0,00000	0,00000	0,00000	0,00000	0,00000	0,00000	0,00000	0,00000	0,00000	**0,00000**
	TRBV9*01TRBJ2-1*01	CASSLGRGGSEQFF	0,02889	0,00000	0,00000	0,00000	0,00000	0,00000	0,00000	0,00000	0,00000	0,00000	**0,00000**
	TRBV3-1*01TRBJ1-5*01	CASSQWGGSGQPQHF	0,02599	0,00000	0,00000	0,00000	0,00000	0,00000	0,00000	0,00000	0,00000	0,00000	**0,00000**
	TRBV11-3*04TRBJ1-4*01	CASSLNHLANEKLFF	0,02560	0,00000	0,00000	0,00633	0,00000	0,00000	0,00000	0,00000	0,00000	0,00000	**0,00000**
	TRBV19*01TRBJ2-2*01	CASSRGELFF	0,02541	0,00000	0,00000	0,00000	0,00000	0,00000	0,00000	0,00000	0,00000	0,00000	**0,00000**
	TRBV7-3*02TRBJ2-5*01	RASSEVRTSL#QETQYF	0,02522	0,00000	0,00000	0,00000	0,00000	0,00000	0,00000	0,00000	0,00000	0,00000	**0,00000**
	TRBV21-1*01TRBJ1-2*01	CASSKVSRGQQ#NYGYTF	0,02444	0,00000	0,00000	0,00000	0,00000	0,00000	0,00000	0,00000	0,00000	0,00000	**0,00000**
	TRBV7-2*04TRBJ2-1*01	CANTRGGYNEQFF	0,02251	0,00000	0,00000	0,00000	0,00000	0,00000	0,00000	0,00000	0,00000	0,00000	**0,00000**
	TRBV19*01TRBJ1-1*01	CASSIGTGTEAFF	0,02184	0,00000	0,00000	0,00000	0,00000	0,00000	0,00000	0,00000	0,00000	0,00000	**0,00000**
	TRBV9*01TRBJ1-1*01	CASSVWDRTNTEAFF	0,01884	0,00000	0,00000	0,00000	0,00000	0,00000	0,00000	0,00000	0,00000	0,00000	**0,00000**
	TRBV19*01TRBJ1-5*01	CASSTQEVSNQPQHF	0,01333	0,00000	0,00000	0,00000	0,00000	0,00000	0,00000	0,00000	0,00000	0,00000	**0,00000**
	TRBV4-3*01TRBJ2-7*01	CASSQDLATADEQYF	0,00000	0,38999	0,20456	0,00000	0,00000	0,00000	0,00000	0,00000	0,00000	0,00000	**0,00000**
	TRBV21-1*01TRBJ1-5*01	CASSKATGGFSQPQHF	0,00000	0,14532	0,22848	0,00000	0,00000	0,00000	0,00000	0,00000	0,00000	0,00000	**0,00000**
	TRBV19*01TRBJ1-1*01	CASSIDSGTEAFF	0,00000	0,04061	0,06047	0,00000	0,00000	0,00000	0,00000	0,00000	0,00000	0,00000	**0,00000**
	TRBV6-3*01TRBJ1-1*01	CASSYPPGDTEAFF	0,00000	0,03481	0,00000	0,00000	0,00000	0,00000	0,00000	0,00000	0,00000	0,00000	**0,00000**
	TRBV12-4*02TRBJ1-5*01	CASSAGGTNQPQHF	0,00000	0,02567	0,01398	0,00000	0,00000	0,00000	0,00000	0,00000	0,00000	0,00000	**0,00000**
	TRBV6-6*01TRBJ1-5*01	CASSSSTAYSNQPQHF	0,00000	0,02059	0,00937	0,00000	0,00000	0,00000	0,00000	0,00000	0,00000	0,00000	**0,00000**
	TRBV11-3*03TRBJ1-6*02	CASSPLGSSYNSPLHF	0,00000	0,01508	0,00000	0,00000	0,00000	0,00000	0,00000	0,00000	0,00000	0,00000	**0,00000**

TRBV24/OR9-2*01TRBJ2-7*01	CAPRKGPPTHEQYF	0,00000	0,01276	0,00000	0,00000	0,00000	0,00000	0,00000	0,00000	0,00000	0,00000	**0,00000**
TRBV28*01TRBJ2-1*01	CASSQSQGSIDNEQFF	0,00000	0,01276	0,00000	0,00000	0,00000	0,00000	0,00000	0,00000	0,00000	0,00000	**0,00000**
TRBV3-2*03TRBJ1-2*01	CASSRAGDYGYTF	0,00000	0,01233	0,00000	0,00000	0,00000	0,00000	0,00000	0,00000	0,00000	0,00000	**0,00000**
TRBV7-2*04TRBJ2-2*01	CASSPTLTGELFF	0,00000	0,01015	0,00000	0,00000	0,00000	0,00000	0,00000	0,00000	0,00000	0,00000	**0,00000**
TRBV19*01TRBJ2-1*01	CASSIGWRGRSYNEQFF	0,00000	0,00957	0,00000	0,00000	0,00000	0,00000	0,00000	0,00000	0,00000	0,00000	**0,00000**
TRBV23-1*01TRBJ1-4*01	CASSPLSARA*RG#EKLFF	0,00000	0,00000	0,00000	0,00000	0,22565	0,22125	0,35105	0,11516	0,09407	0,23047	0,03134
TRBV5-4*02TRBJ2-2*01	CASNSRGPGELFF	0,00000	0,00000	0,00000	0,00000	0,18918	0,19619	0,47483	0,09507	0,06765	0,22259	0,02555
TRBV4-1*01TRBJ1-2*01	CASSQDLGPRDSYTF	0,00000	0,00000	0,00000	0,00000	0,00008	0,03099	0,00117	0,00000	0,02568	0,00000	**0,00000**
TRBV12-4*01TRBJ2-1*01	CASSLYGDNEQFF	0,00000	0,00000	0,00000	0,00000	0,02470	0,01973	0,05754	0,01831	0,03556	0,06510	**0,00000**
TRBV5-1*01TRBJ2-1*01	CASSLGGGYNEQFF	0,00000	0,00000	0,00000	0,00000	0,01482	0,01489	0,00263	0,00000	0,00000	0,00000	**0,00000**
TRBV6-4*01TRBJ2-7*01	CATGTGVYEQYF	0,00000	0,00000	0,00000	0,00000	0,00165	0,00853	0,01890	0,00000	0,00000	0,00000	**0,00000**
TRBV4-1*02TRBJ2-7*01	CASGTGFLEQYF	0,00000	0,00000	0,00000	0,00000	0,01070	0,00825	0,01010	0,00000	0,00000	0,00000	**0,00000**
TRBV4-1*02TRBJ2-2*01	CASSLNGTI*#TGELFF	0,00000	0,00000	0,00000	0,00000	0,00000	0,00613	0,00000	0,00000	0,00000	0,00000	**0,00000**
TRBV20-1*02TRBJ2-3*01	CSPGGG#SADTQYF	0,00000	0,00000	0,00000	0,00000	0,00000	0,00000	0,00000	0,00024	0,00038	0,00000	0,00875
TRBV20/OR9-2*01TRBJ2-2*01	CSARGGFTGELFF	0,00000	0,00000	0,00000	0,00000	0,00000	0,00000	0,00000	0,00142	0,00031	0,00122	0,00699
TRBV12-4*02TRBJ2-7*01	CASSSPSGGASHEQYF	0,00000	0,00000	0,00000	0,00000	0,00000	0,00000	0,00000	0,00788	0,00038	0,00665	0,00384
TRBV20-1*01TRBJ2-7*01	CSARDLDRSSYEQYF	0,00000	0,00000	0,00000	0,02913	0,00000	0,00000	0,00000	0,00000	0,00000	0,00000	**0,00000**
TRBV29-1*01TRBJ2-7*01	CSVEVPYEQYF	0,00000	0,00000	0,00000	0,05993	0,00000	0,00000	0,00000	0,00000	0,00000	0,00000	**0,00000**
TRBV5-1*02TRBJ1-2*01	CASFPGTSPYGYTF	0,00000	0,00000	0,00000	0,00263	0,00000	0,00000	0,00000	0,00000	0,00000	0,00000	**0,00000**
TRBV6-5*01TRBJ1-4*01	CASSDVAGPGEKLFF	0,00000	0,00000	0,00000	0,00000	0,00000	0,00000	0,00773	0,00403	0,00579	0,00000	**0,00000**
TRBV10-3*01TRBJ1-1*01	CAMGRGVDTEAFF	0,00000	0,00000	0,00000	0,00000	0,00453	0,00000	0,01181	0,00000	0,00000	0,00000	**0,00000**
TRBV10-3*01TRBJ2-2*01	CAIKGTSGNTGELFF	0,00000	0,00000	0,00000	0,00000	0,00000	0,00000	0,00865	0,00000	0,00000	0,00000	**0,00000**
TRBV26/OR9-2*01TRBJ1-4*01	CASSSLSARA*RG#EKLFF	0,00000	0,00000	0,00000	0,00000	0,00008	0,00000	0,00367	0,00000	0,00000	0,00000	**0,00000**
TRBV5-4*01TRBJ2-1*01	CASSFMSGVHEQFF	0,00000	0,00000	0,00000	0,00000	0,00000	0,00000	0,00319	0,00000	0,00000	0,00000	**0,00000**
TRBV6-6*05TRBJ1-5*01	CASSVRNSNQPQHF	0,00000	0,00000	0,00000	0,00000	0,00420	0,00000	0,00000	0,00000	0,00000	0,00000	**0,00000**
TRBV6-3*01TRBJ2-1*01	CASSYSLTGGHYNEQFF	0,00000	0,00000	0,00000	0,01874	0,00000	0,00000	0,00000	0,00000	0,00000	0,00000	**0,00000**
TRBV28*01TRBJ1-2*01	CASSLAGGYTF	0,00000	0,00000	0,00000	0,01660	0,00000	0,00000	0,00000	0,00000	0,00000	0,00000	**0,00000**
TRBV7-2*04TRBJ2-5*01	CASSSGREETQYF	0,00000	0,00000	0,00000	0,01433	0,00000	0,00000	0,00000	0,00000	0,00000	0,00000	**0,00000**
TRBV9*01TRBJ2-7*01	CASSVVGTGLYEQYF	0,00000	0,00000	0,00000	0,01433	0,00000	0,00000	0,00000	0,00000	0,00000	0,00000	**0,00000**
TRBV6-4*01TRBJ2-1*01	CASSDSPAGEQFF	0,00000	0,00000	0,00000	0,01373	0,00000	0,00000	0,00000	0,00000	0,00000	0,00000	**0,00000**
TRBV9*01TRBJ2-2*01	CASSDGFGELFF	0,00000	0,00000	0,00000	0,01074	0,00000	0,00000	0,00000	0,00000	0,00000	0,00000	**0,00000**
TRBV28*01TRBJ2-7*01	CASSSPDEQYF	0,00000	0,00000	0,00000	0,01039	0,00000	0,00000	0,00000	0,00000	0,00000	0,00000	**0,00000**
TRBV9*01TRBJ2-5*01	CASSVHLAGTPETQYF	0,00000	0,00000	0,00000	0,01039	0,00000	0,00000	0,00000	0,00000	0,00000	0,00000	**0,00000**
TRBV13*01TRBJ2-7*01	CASSFRVYEQYF	0,00000	0,00000	0,00000	0,00991	0,00000	0,00000	0,00000	0,00000	0,00000	0,00000	**0,00000**
TRBV5-5*02TRBJ2-7*01	CASSRGQYYEQYF	0,00000	0,00000	0,00000	0,00991	0,00000	0,00000	0,00000	0,00000	0,00000	0,00000	**0,00000**
TRBV7-8*03TRBJ2-1*01	CASRGRRAPEQFF	0,00000	0,00000	0,00000	0,00979	0,00000	0,00000	0,00000	0,00000	0,00000	0,00000	**0,00000**

	TRBV7-9*04TRBJ2-1*01	CASSRIPETGSNEQFF	0,00000	0,00000	0,00000	0,00979	0,00000	0,00000	0,00000	0,00000	0,00000	0,00000	**0,00000**
	TRBV19*01TRBJ2-1*01	CASSIGGNNEQFF	0,00000	0,00000	0,00000	0,00967	0,00000	0,00000	0,00000	0,00000	0,00000	0,00000	**0,00000**
	TRBV4-1*01TRBJ1-2*01	CASSQDHRFQGIGYTF	0,00000	0,00000	0,00000	0,00955	0,00000	0,00000	0,00000	0,00000	0,00000	0,00000	**0,00000**
	TRBV2*02TRBJ1-2*01	CASNLGRFYGYTF	0,00000	0,00000	0,01160	0,00000	0,00000	0,00000	0,00000	0,00000	0,00000	0,00000	**0,00000**
	TRBV10-3*01TRBJ2-3*01	CAISRTSGDTDTQYF	0,00000	0,00000	0,02177	0,00000	0,00000	0,00000	0,00000	0,00000	0,00000	0,00000	**0,00000**
	TRBV7-9*04TRBJ2-1*01	CASLITGTNEQFF	0,00000	0,00000	0,03150	0,00000	0,00000	0,00000	0,00000	0,00000	0,00000	0,00000	**0,00000**
	TRBV7-3*02TRBJ1-5*01	RASTQ#NQPQHF	0,00000	0,00000	0,03662	0,00000	0,00000	0,00000	0,00000	0,00000	0,00000	0,00000	**0,00000**
	TRBV2*03TRBJ2-1*01	CASSEWGTSGNEQFF	0,00000	0,00000	0,09132	0,00000	0,00000	0,00000	0,00000	0,00000	0,00000	0,00000	**0,00000**
**TCRalpha repertoire**	**alpha gene rearrangement**	**Junction AA (around half non coding)**	**% of rearrangement withing all TCRA gene rearrangements**										
	**TRAV14/DV4*01TRAJ11*01**	**CAS#NSGYSTLTF**	**0,03814**	**0,29705**	**0,24407**	**0,21687**	**0,39149**	**0,41838**	**0,03566**	**0,50507**	**0,52281**	**0,44057**	**0,63608**
	**TRAV21*01TRAJ33*01**	**CAAKDSNYQLIW**	**0,03002**	**0,07668**	**0,15001**	**0,09359**	**0,18157**	**0,17242**	**0,01076**	**0,28760**	**0,28133**	**0,14492**	**0,28128**
	TRAV21*01TRAJ41*01	CAVRHSP#SNSGYALNF	0,03836	0,00000	0,00000	0,00000	0,00000	0,00000	0,00000	0,00000	0,00000	0,00000	0,00000
	TRAV25*01TRAJ43*01	CAGSNNNDMRF	0,02863	0,00000	0,00000	0,00000	0,00000	0,00000	0,00000	0,00000	0,00000	0,00000	0,00000
	TRAV30*01TRAJ7*01	CGTGVYYGNNRLAF	0,02789	0,00000	0,00000	0,00000	0,00000	0,00000	0,00000	0,00000	0,00000	0,00000	0,00000
	TRAV13-1*01TRAJ37*02	CAAKIH#GSSNTGKLIF	0,02025	0,00000	0,00000	0,00000	0,00000	0,00000	0,00000	0,00000	0,00000	0,00000	0,00000
	TRAV27*01TRAJ42*01	CAGAR#GGSQGNLIF	0,01846	0,00000	0,00000	0,00000	0,00000	0,00000	0,00000	0,00000	0,00000	0,00000	0,00000
	TRAV9-2*01TRAJ53*01	CALNSGGSNYKLTF	0,01833	0,00000	0,00000	0,00000	0,00000	0,00000	0,00000	0,00000	0,00000	0,00000	0,00000
	TRAV8-4*01TRAJ34*01	CAVTLYNTDKLIF	0,01802	0,00000	0,00000	0,00000	0,00000	0,00000	0,00000	0,00000	0,00000	0,00000	0,00000
	TRAV5*01TRAJ42*01	CADLTQ#NYGGSQGNLIF	0,01676	0,00000	0,00000	0,00000	0,00000	0,00000	0,00000	0,00000	0,00000	0,00000	0,00000
	TRAV19*01TRAJ24*02	CALMTTDSWGKLQF	0,01650	0,00000	0,00000	0,00000	0,00000	0,00000	0,00000	0,00000	0,00000	0,00000	0,00000
	TRAV16*01TRAJ13*02	CALLSGGYQKVTF	0,01632	0,00000	0,00000	0,00000	0,00000	0,00000	0,00000	0,00000	0,00000	0,00000	0,00000
	TRAV6*02TRAJ3*01	CAPA#GYSSASKIIF	0,01632	0,00000	0,00000	0,00000	0,00000	0,00000	0,00000	0,00000	0,00000	0,00000	0,00000
	TRAV12-3*01TRAJ54*01	CAMSAGAQKLVF	0,01567	0,00000	0,00000	0,00000	0,00000	0,00000	0,00000	0,00000	0,00000	0,00000	0,00000
	TRAV24*01TRAJ40*01	CAPHSGTYKYIF	0,01545	0,00000	0,00000	0,00000	0,00000	0,00000	0,00000	0,00000	0,00000	0,00000	0,00000
	TRAV9-2*01TRAJ11*01	CALSDRESGYSTLTF	0,01510	0,00000	0,00000	0,00000	0,00000	0,00000	0,00000	0,00000	0,00000	0,00000	0,00000
	TRAV39*01TRAJ56*01	CAVDTGANSKLTF	0,01501	0,00000	0,00000	0,00000	0,00000	0,00000	0,00000	0,00000	0,00000	0,00000	0,00000
	TRAV17*01TRAJ48*01	CATDSM#NFGNEKLTF	0,01418	0,00000	0,00000	0,00000	0,00000	0,00000	0,00000	0,00000	0,00000	0,00000	0,00000
	TRAV8-2*01TRAJ37*02	CVVR#GSSNTGKLIF	0,01396	0,00000	0,00000	0,00000	0,00000	0,00000	0,00000	0,00000	0,00000	0,00000	0,00000
	TRAV8-2*02TRAJ16*02	CVV#YGQKLLFARG	0,01314	0,00000	0,00000	0,00000	0,00000	0,00000	0,00000	0,00000	0,00000	0,00000	0,00000
	TRAV35*01TRAJ23*01	CAGRNNQGGKLIF	0,01274	0,00000	0,00000	0,00000	0,00000	0,00000	0,00000	0,00000	0,00000	0,00000	0,00000
	TRAV23/DV6*01TRAJ54*01	CAAIVPKQGAQKLVF	0,01183	0,00000	0,00000	0,00000	0,00000	0,00000	0,00000	0,00000	0,00000	0,00000	0,00000
	TRAV12-3*01TRAJ43*01	CAM#NDMRF	0,01161	0,00000	0,00000	0,00000	0,00000	0,00000	0,00000	0,00000	0,00000	0,00000	0,00000
	TRAV23/DV6*01TRAJ36*01	CAALP#GANNLFF	0,00419	0,00000	0,00000	0,00888	0,00000	0,00000	0,00000	0,00000	0,00000	0,00000	0,00000
	TRAV12-1*01TRAJ31*01	CVVENARLMF	0,00144	0,00000	0,00000	0,00304	0,00000	0,00000	0,00000	0,00000	0,00000	0,00000	0,00000
	TRAV25*01TRAJ53*01	CAGGGSNYKLTF	0,00000	0,11878	0,13175	0,00000	0,00000	0,00000	0,00000	0,00000	0,00000	0,00000	0,00000
	TRAV13-1*01TRAJ49*01	CAAIITGNQFYF	0,00000	0,04505	0,00000	0,00000	0,00000	0,00000	0,00000	0,00000	0,00000	0,00000	0,00000

TRAV38-1*03TRAJ58*01	CAFP*#ETSGSRLTF	0,00000	0,03264	0,00328	0,00000	0,00000	0,00000	0,00000	0,00000	0,00000	0,00000	0,00000
TRAV26-2*01TRAJ33*01	CILRDVK#GSNYQLIW	0,00000	0,03107	0,00100	0,00000	0,00000	0,00000	0,00000	0,00000	0,00000	0,00000	0,00000
TRAV13-1*01TRAJ45*01	CAATS	0,00000	0,03043	0,01233	0,00000	0,00000	0,00000	0,00000	0,00000	0,00000	0,00000	0,00000
TRAV13-1*01TRAJ48*01	CAASETNFGNEKLTF	0,00000	0,02648	0,00000	0,00000	0,00000	0,00000	0,00000	0,00000	0,00000	0,00000	0,00000
TRAV22*01TRAJ43*01	CAVEKLYNNNDMRF	0,00000	0,02151	0,00000	0,00000	0,00000	0,00000	0,00000	0,00000	0,00000	0,00000	0,00000
TRAV8-4*01TRAJ22*01	CAVSGSARQLTF	0,00000	0,02041	0,00477	0,00000	0,00000	0,00000	0,00000	0,00000	0,00000	0,00000	0,00000
TRAV12-1*01TRAJ5*01	CVVNRGTGRRALTF	0,00000	0,01655	0,00000	0,00000	0,00000	0,00000	0,00000	0,00000	0,00000	0,00000	0,00000
TRAV9-2*01TRAJ22*01	CALSDFTP#GSARQLTF	0,00000	0,01499	0,00000	0,00000	0,00000	0,00000	0,00000	0,00000	0,00000	0,00000	0,00000
TRAV12-2*02TRAJ22*01	CFSGSARQLTF	0,00000	0,01333	0,00000	0,00000	0,00000	0,00000	0,00000	0,00000	0,00000	0,00000	0,00000
TRAV9-1*01TRAJ11*01	CASE#SGYSTLTF	0,00000	0,01324	0,00731	0,00000	0,00133	0,00085	0,00055	0,00967	0,00842	0,00938	0,00581
TRAV41*01TRAJ42*01	CAVENYGGSQGNLIF	0,00000	0,01048	0,00274	0,00000	0,00000	0,00000	0,00000	0,00000	0,00000	0,00000	0,00000
TRAV20*01TRAJ4*01	CAVQGVFSGGYNKLIF	0,00000	0,01039	0,00000	0,00000	0,00000	0,00000	0,00000	0,00000	0,00000	0,00000	0,00000
TRAV23/DV6*01TRAJ31*01	CAASK#ARLMF	0,00000	0,00717	0,01498	0,00000	0,00000	0,00000	0,00000	0,00000	0,00000	0,00000	0,00000
TRAV8-1*01TRAJ29*01	CAVI#NSGNTPLVF	0,00000	0,00349	0,06496	0,00000	0,00000	0,00000	0,00000	0,00000	0,00000	0,00000	0,00000
TRAV16*01TRAJ39*01	CALSGRNAGNMLTF	0,00000	0,00000	0,08036	0,00000	0,00000	0,00000	0,00000	0,00000	0,00000	0,00000	0,00000
TRAV9-2*01TRAJ49*01	CALSGCV#NTGNQFYF	0,00000	0,00000	0,03819	0,00000	0,00000	0,00000	0,00000	0,00000	0,00000	0,00000	0,00000
TRAV8-6*01TRAJ32*02	CAVSEGGATNKLIF	0,00000	0,00000	0,03171	0,00000	0,00000	0,00000	0,00000	0,00000	0,00000	0,00000	0,00000
TRAV12-2*03TRAJ47*01	CAVN*#GNKLVF	0,00000	0,00000	0,02669	0,00000	0,00000	0,00000	0,00000	0,00000	0,00000	0,00000	0,00000
TRAV9-2*01TRAJ20*01	CALSDSNDYKLSF	0,00000	0,00000	0,01955	0,00000	0,00000	0,00000	0,00000	0,00000	0,00000	0,00000	0,00000
TRAV25*01TRAJ57*01	CAVITQGGSEKLVF	0,00000	0,00000	0,00000	0,03801	0,00183	0,00000	0,00000	0,00073	0,00000	0,00000	0,00000
TRAV1-2*01TRAJ31*01	CAGLTRNNARLMF	0,00000	0,00000	0,00000	0,01571	0,00000	0,00000	0,00000	0,00000	0,00000	0,00000	0,00000
TRAV12-2*01TRAJ53*01	CAVN#SGGSNYKLTF	0,00000	0,00000	0,00000	0,01199	0,00000	0,00000	0,00000	0,00000	0,00000	0,00000	0,00000
TRAV14/DV4*03TRAJ29*01	CAMI#NSGNTPLVF	0,00000	0,00000	0,00000	0,01143	0,00000	0,00000	0,00000	0,00000	0,00000	0,00000	0,00000
TRAV8-1*01TRAJ28*01	CAVTRG#YS	0,00000	0,00000	0,00000	0,00913	0,00000	0,00000	0,00000	0,00000	0,00000	0,00000	0,00000
TRAV23/DV6*02TRAJ57*01	CAASVQGGSEKLVF	0,00000	0,00000	0,00000	0,00901	0,00000	0,00000	0,00000	0,00000	0,00000	0,00000	0,00000
TRAV27*01TRAJ52*01	CAGAPDGTSYGKLTF	0,00000	0,00000	0,00000	0,00807	0,00000	0,00000	0,00000	0,00000	0,00000	0,00000	0,00000
TRAV13-1*01TRAJ44*01	CAA#NTGTASKLTF	0,00000	0,00000	0,00000	0,00758	0,00000	0,00000	0,00000	0,00000	0,00000	0,00000	0,00000
TRAV39*01TRAJ40*01	CAVDMNWGTYKYIF	0,00000	0,00000	0,00000	0,00708	0,00000	0,00000	0,00000	0,00000	0,00000	0,00000	0,00000
TRAV25*01TRAJ47*01	CAASMEYGNKLVF	0,00000	0,00000	0,00000	0,00702	0,00000	0,00000	0,00000	0,00000	0,00000	0,00000	0,00000
TRAV9-2*01TRAJ43*01	CALSEHDMRF	0,00000	0,00000	0,00000	0,00633	0,00000	0,00000	0,00000	0,00000	0,00000	0,00000	0,00000
TRAV26-2*01TRAJ48*01	CILRLPISNFGNEKLTF	0,00000	0,00000	0,00000	0,00609	0,00000	0,00000	0,00000	0,00000	0,00000	0,00000	0,00000
TRAV17*01TRAJ6*01	CATDSGGSYIPTF	0,00000	0,00000	0,00000	0,00509	0,00000	0,00000	0,00000	0,00000	0,00000	0,00000	0,00000
TRAV16*01TRAJ30*01	CALRP#RDDKIIF	0,00000	0,00000	0,00000	0,00472	0,00000	0,00000	0,00000	0,00000	0,00000	0,00000	0,00000
TRAV21*02TRAJ3*01	CALE#SASKIIF	0,00000	0,00000	0,00000	0,00385	0,00000	0,00000	0,00000	0,00000	0,00000	0,00000	0,00000
TRAV13-1*01TRAJ28*01	CAAG#GAGSYQLTFGKGTKLSVI#T	0,00000	0,00000	0,00000	0,00000	0,18881	0,13617	0,41571	0,07002	0,04864	0,13735	0,01720
TRAV25*01TRAJ12*01	CAGVADSSYKLIF	0,00000	0,00000	0,00000	0,00000	0,10982	0,10500	0,33380	0,03639	0,03827	0,10979	0,00946
TRAV17*01TRAJ53*01	CASSGGSNYKLTF	0,00000	0,00000	0,00000	0,00000	0,00972	0,00000	0,00571	0,01662	0,00832	0,02769	0,00000
TRAV13-1*01TRAJ20*01	CAASRGSNDYKLSF	0,00000	0,00000	0,00000	0,00000	0,00675	0,00525	0,01421	0,00399	0,00818	0,03147	0,00000

	TRAV1-1*01TRAJ24*02	CAVRDL#TTDSWGKLQF	0,00000	0,00000	0,00000	0,00000	0,00557	0,00170	0,01753	0,00000	0,00000	0,00000	0,00000
	TRAV17*01TRAJ3*01	CAVYSSASKIIF	0,00000	0,00000	0,00000	0,00000	0,00526	0,00000	0,00000	0,00000	0,00000	0,00000	0,00000
	TRAV26-1*01TRAJ12*01	CIVRVE#VDSSYKLIF	0,00000	0,00000	0,00000	0,00000	0,00480	0,00560	0,02296	0,00376	0,00411	0,03387	0,00112
	TRAV5*01TRAJ3*01	CAAASGYSSASKIIF	0,00000	0,00000	0,00000	0,00000	0,00476	0,00000	0,00000	0,00000	0,00000	0,00000	0,00000
	TRAV29/DV5*02TRAJ28*01	CAAG#GAGSYQLTFGKGTKLSVIPSKFFFLANYSSREACLPSCRSCQNTGGVFFAWFVLGGRT	0,00000	0,00000	0,00000	0,00000	0,00454	0,00139	0,00000	0,00261	0,00000	0,00000	0,00000
	TRAV2*01TRAJ27*01	CAVSF#NTNAGKSTF	0,00000	0,00000	0,00000	0,00000	0,00362	0,00345	0,00000	0,00000	0,00000	0,00000	0,00000
	TRAV10*01TRAJ38*01	CVVSA#CWQQP*ADL	0,00000	0,00000	0,00000	0,00000	0,00305	0,00000	0,00000	0,00331	0,00000	0,00487	0,00000
	TRAV17*01TRAJ23*01	FPLSNQGGKLIF	0,00000	0,00000	0,00000	0,00000	0,00274	0,00701	0,00931	0,00000	0,00000	0,00000	0,00000
	TRAV9-2*01TRAJ7*01	CALPSSI#YGNNRLAF	0,00000	0,00000	0,00000	0,00000	0,00179	0,01085	0,00027	0,00000	0,00000	0,00000	0,00000
	TRAV35*01TRAJ39*01	CAGRG#AGNMLTF	0,00000	0,00000	0,00000	0,00000	0,00137	0,00975	0,00280	0,00000	0,00000	0,00000	0,00000
	TRAV17*01TRAJ29*01	CAYKR#NSGNTPLVF	0,00000	0,00000	0,00000	0,00000	0,00057	0,00561	0,00000	0,00000	0,00000	0,00000	0,00000
	TRAV13-2*02TRAJ52*01	CADDAGGTSYGKLTF	0,00000	0,00000	0,00000	0,00000	0,00000	0,00714	0,00000	0,00000	0,00212	0,00000	0,00000
	TRAV22*01TRAJ36*01	CAVVPGANNLFF	0,00000	0,00000	0,00000	0,00000	0,00000	0,00226	0,01243	0,00000	0,00000	0,00000	0,00000
	TRAV16*01TRAJ13*02	CALRSGGYQKVTF	0,00000	0,00000	0,00000	0,00000	0,00000	0,00019	0,01417	0,00000	0,00000	0,00000	0,00000
	TRAV6*02TRAJ3*01	CAPW#SSASKIIF	0,00000	0,00000	0,00000	0,00000	0,00000	0,00000	0,00000	0,00331	0,00902	0,00187	0,00151
	TRAV26-1*01TRAJ37*02	CIVPSRGSSNTGKLIF	0,00000	0,00000	0,00000	0,00000	0,00000	0,00000	0,00188	0,00115	0,00552	0,00303	0,00109
	TRAV23/DV6*01TRAJ36*01	CAAR#QTGANNLFF	0,00000	0,00000	0,00000	0,00000	0,00000	0,00000	0,00757	0,00000	0,00000	0,00000	0,00000
	TRAV8-3*01TRAJ48*01	CAVGAIGNEKLTF	0,00000	0,00000	0,00000	0,00000	0,00000	0,00000	0,00765	0,00000	0,00000	0,00000	0,00000
TCRgamma repertoire	**gamma gene rearrangement**	**Junction AA (Mostly non coding)**	**% of rearrangement withing all TCRG gene rearrangements**										
	TRGV8*01TRGJ1*02	CATPPS#F	0,04202	0,05079	0,09854	0,11817	0,24480	0,15118	0,01908	0,35088	0,26266	0,13218	0,38777
	TRGV4*02TRGJ1*02	CATWDG#YKKLF	0,01195	0,14980	0,13054	0,13105	0,21801	0,25380	0,00973	0,39227	0,36846	0,21497	0,43666
	TRGV9*01TRGJ1*02	CALWEVLFW#KLF	0,02509	0,00000	0,00000	0,00000	0,00000	0,00000	0,00000	0,00000	0,00000	0,00000	0,00000
	TRGV10*02TRGJ1*01	CAAWDLAR#KLF	0,02357	0,00000	0,00000	0,00000	0,00000	0,00000	0,00000	0,00000	0,00000	0,00000	0,00000
	TRGV11*01TRGJ2*01	CACWIRHP#YKKLF	0,02172	0,00000	0,00000	0,00000	0,00000	0,00000	0,00000	0,00000	0,00000	0,00000	0,00000
	TRGV10*02TRGJ1*01	CAAWDLWKLF	0,02144	0,00000	0,00000	0,00000	0,00000	0,00000	0,00000	0,00000	0,00000	0,00000	0,00000
	TRGV8*01TRGJP2*01	CATPW#SDWIKTF	0,02129	0,00000	0,00000	0,00000	0,00000	0,00000	0,00000	0,00000	0,00000	0,00000	0,00000
	TRGV3*02TRGJ1*02	CATRGNKKLF	0,02096	0,00000	0,00000	0,00000	0,00000	0,00000	0,00000	0,00000	0,00000	0,00000	0,00000
	TRGV3*02TRGJP2*01	CATWANSSDWIKTF	0,02082	0,00000	0,00000	0,00000	0,00000	0,00000	0,00000	0,00000	0,00000	0,00000	0,00000
	TRGV10*02TRGJP1*01	CAAWDLGE#TTGWFKIF	0,02077	0,00000	0,00000	0,00000	0,00000	0,00000	0,00000	0,00000	0,00000	0,00000	0,00000
	TRGV9*01TRGJ2*01	CALWEVPP#YKKLF	0,02049	0,00000	0,00000	0,00000	0,00000	0,00000	0,00000	0,00000	0,00000	0,00000	0,00000
	TRGV4*01TRGJ2*01	CATWDG#YYKKLF	0,01916	0,00000	0,00000	0,00000	0,00000	0,00000	0,00000	0,00000	0,00000	0,00000	0,00000
	TRGV11*01TRGJ1*02	CACWNLF	0,01897	0,00000	0,00000	0,00000	0,00000	0,00000	0,00000	0,00000	0,00000	0,00000	0,00000
	TRGV8*01TRGJP1*01	CATWDRR#TGWFKIF	0,01854	0,00000	0,00000	0,00000	0,00000	0,00000	0,00000	0,00000	0,00000	0,00000	0,00000

TRGV3*01TRGJ1*02	CATWDTKKLF	0,01840	0,00000	0,00000	0,00000	0,00000	0,00000	0,00000	0,00000	0,00000	0,00000	0,00000
TRGV10*02TRGJ1*01	CAAWDSYYKKLF	0,01826	0,00000	0,00000	0,00000	0,00000	0,00000	0,00000	0,00000	0,00000	0,00000	0,00000
TRGV3*01TRGJ1*02	CAT#NYYKKLF	0,01712	0,00000	0,00000	0,00000	0,00000	0,00000	0,00000	0,00000	0,00000	0,00000	0,00000
TRGV2*02TRGJ2*01	CATPPS#F	0,01684	0,00714	0,00958	0,00978	0,00772	0,00139	0,00000	0,04091	0,02007	0,01204	0,03758
TRGV10*02TRGJ1*02	CAAWD*W#YKKLF	0,01674	0,00000	0,00000	0,00000	0,00000	0,00000	0,00000	0,00000	0,00000	0,00000	0,00000
TRGV4*02TRGJ1*01	CAIK#KKLF	0,01655	0,00000	0,00000	0,00000	0,00000	0,00000	0,00000	0,00000	0,00000	0,00000	0,00000
TRGV8*01TRGJP2*01	CATWE#SSDWIKTF	0,01560	0,00000	0,00000	0,00000	0,00000	0,00000	0,00000	0,00000	0,00000	0,00000	0,00000
TRGV9*01TRGJP*01	CAL*K#ELGKKIKVF	0,01546	0,00000	0,00000	0,00000	0,00000	0,00000	0,00000	0,00000	0,00000	0,00000	0,00000
TRGV4*02TRGJP1*01	CATWDP#TGWFKIF	0,01437	0,00000	0,00000	0,00636	0,00000	0,00000	0,00000	0,00000	0,00000	0,00000	0,00000
TRGV9*01TRGJ1*02	CALWP#F	0,01418	0,00000	0,00000	0,00000	0,00000	0,00000	0,00000	0,00000	0,00000	0,00000	0,00000
TRGV4*01TRGJ1*02	CATY#NYYKKLF	0,01091	0,00000	0,00000	0,00000	0,00000	0,00000	0,00000	0,00000	0,00000	0,00000	0,00000
TRGV10*02TRGJP2*01	CAAWD*GSSDWIKTF	0,00441	0,00000	0,00000	0,00000	0,00586	0,00023	0,00000	0,00000	0,00000	0,00000	0,00000
TRGV10*02TRGJ1*02	CAASR#KLF	0,00000	0,22698	0,13233	0,00000	0,00000	0,00000	0,00000	0,00000	0,00000	0,00000	0,00000
TRGV4*01TRGJ1*02	CATW#KKLF	0,00000	0,08244	0,09223	0,00000	0,00000	0,00000	0,00000	0,00000	0,00000	0,00000	0,00000
TRGV9*01TRGJ1*02	CALWEVRE#YKKLF	0,00000	0,02579	0,00346	0,00000	0,00000	0,00000	0,00000	0,00000	0,00000	0,00000	0,00000
TRGV4*02TRGJ1*02	CATSFILA#KKLF	0,00000	0,02083	0,00000	0,00000	0,00000	0,00000	0,00000	0,00000	0,00000	0,00000	0,00000
TRGV10*02TRGJ1*02	CAAWDY*KLF	0,00000	0,01964	0,06245	0,00000	0,00000	0,00000	0,00000	0,00000	0,00000	0,00000	0,00000
TRGV10*02TRGJ1*02	CAAWENYYKKLF	0,00000	0,01954	0,00709	0,00000	0,00000	0,00000	0,00000	0,00000	0,00000	0,00000	0,00000
TRGV10*02TRGJ2*01	CAAWE#YKKLF	0,00000	0,01925	0,00000	0,00000	0,00000	0,00000	0,00000	0,00000	0,00000	0,00000	0,00000
TRGV10*02TRGJ1*02	CAAFLKKLF	0,00000	0,01518	0,00000	0,00000	0,00000	0,00000	0,00000	0,00000	0,00000	0,00000	0,00000
TRGV2*01TRGJ2*01	CATWDGLHR#KKLF	0,00000	0,01508	0,00000	0,00000	0,00000	0,00000	0,00000	0,00000	0,00000	0,00000	0,00000
TRGV5*01TRGJ1*01	CATWDRLPK#KLF	0,00000	0,01458	0,00000	0,00000	0,00000	0,00000	0,00000	0,00000	0,00000	0,00000	0,00000
TRGV4*02TRGJ1*02	CATWDGQR#NYYKKLF	0,00000	0,01369	0,00892	0,00000	0,00000	0,00000	0,00000	0,00000	0,00000	0,00000	0,00000
TRGV2*02TRGJ1*02	CATWA*#KKLF	0,00000	0,01329	0,00000	0,00000	0,00000	0,00000	0,00000	0,00000	0,00000	0,00000	0,00000
TRGV8*01TRGJ1*02	CATW#KKLF	0,00000	0,01319	0,00230	0,00000	0,00000	0,00000	0,00000	0,00000	0,00000	0,00000	0,00000
TRGV5P*01TRGJ1*02	CATWGV#KLF	0,00000	0,01260	0,00000	0,00000	0,00000	0,00000	0,00000	0,00000	0,00000	0,00000	0,00000
TRGV10*02TRGJ1*01	CAAWD#WKLF	0,00000	0,01240	0,00000	0,00000	0,00000	0,00000	0,00000	0,00000	0,00000	0,00000	0,00000
TRGV4*01TRGJ1*02	CATWDGQRLF	0,00000	0,01131	0,00000	0,00000	0,00000	0,00000	0,00000	0,00000	0,00000	0,00000	0,00000
TRGV10*02TRGJ2*01	CAAWD*GDEG#KKLF	0,00000	0,01131	0,00000	0,00000	0,00000	0,00000	0,00000	0,00000	0,00000	0,00000	0,00000
TRGV10*02TRGJ2*01	CAAWENYYKKLF	0,00000	0,01111	0,00000	0,00000	0,00000	0,00000	0,00000	0,00000	0,00000	0,00000	0,00000
TRGV10*02TRGJP1*01	CAAWDD#GWFKIF	0,00000	0,00010	0,04053	0,00000	0,00000	0,00000	0,00000	0,00000	0,00000	0,00000	0,00000
TRGV4*01TRGJ1*02	CATWDGPG#YKKLF	0,00000	0,00010	0,00000	0,00000	0,12314	0,13633	0,31043	0,05403	0,05872	0,14106	0,02115
TRGV2*03TRGJ1*01	CATWDGL#KLF	0,00000	0,00000	0,07919	0,00000	0,00000	0,00000	0,00000	0,00000	0,00000	0,00000	0,00000
TRGV10*02TRGJ1*02	CAAWDYGKLF	0,00000	0,00000	0,04391	0,00000	0,00000	0,00000	0,00000	0,00000	0,00000	0,00000	0,00000
TRGV5*01TRGJ1*02	CATWDSR#YKKLF	0,00000	0,00000	0,04318	0,00000	0,00000	0,00000	0,00000	0,00000	0,00000	0,00000	0,00000
TRGV10*02TRGJ1*01	CAAWPFYYKKPAAWE*YKKLF	0,00000	0,00000	0,03150	0,00000	0,00000	0,00000	0,00000	0,00000	0,00000	0,00000	0,00000
TRGV10*02TRGJP1*01	CAAWVT#TTGWFKIF	0,00000	0,00000	0,02402	0,00000	0,00000	0,00000	0,00000	0,00000	0,00000	0,00000	0,00000
TRGV4*01TRGJ1*01	CRP#KLF	0,00000	0,00000	0,01012	0,00000	0,00000	0,00000	0,00000	0,00000	0,00000	0,00000	0,00000
TRGV9*01TRGJ1*02	CALWEV#KKLF	0,00000	0,00000	0,00973	0,00000	0,00000	0,00000	0,00000	0,00000	0,00000	0,00000	0,00000
TRGV3*02TRGJ1*02	CATWDSR#YKKLF	0,00000	0,00000	0,00732	0,00000	0,00000	0,00000	0,00000	0,00000	0,00000	0,00000	0,00000
TRGV9*01TRGJ1*01	CALWEV#KKLF	0,00000	0,00000	0,00596	0,00000	0,00000	0,00000	0,00000	0,00000	0,00000	0,00000	0,00000

TRGV10*02TRGJP1*01	CAAWG#TTGWFKIF	0,00000	0,00000	0,00000	0,02708	0,00000	0,00000	0,00000	0,00236	0,00000	0,00000	0,00000
TRGV9*01TRGJ1*01	CALWEVQ#YKKLF	0,00000	0,00000	0,00000	0,02568	0,00000	0,00000	0,00000	0,00000	0,00000	0,00000	0,00000
TRGV4*02TRGJ1*01	CATWDGG#KKLF	0,00000	0,00000	0,00000	0,01342	0,00000	0,00000	0,00000	0,00000	0,00000	0,00000	0,00000
TRGV8*01TRGJ1*01	CATWDRFW#YKKLF	0,00000	0,00000	0,00000	0,01304	0,00000	0,00000	0,00000	0,00000	0,00000	0,00000	0,00000
TRGV3*02TRGJ1*02	CATWD#KKLF	0,00000	0,00000	0,00000	0,01055	0,00000	0,00000	0,00000	0,00000	0,00000	0,00000	0,00000
TRGV9*01TRGJ1*02	CALWEVVS#NYYKKLF	0,00000	0,00000	0,00000	0,01009	0,00000	0,00000	0,00000	0,00000	0,00000	0,00000	0,00000
TRGV4*01TRGJ1*02	CATWDVPHYKKLF	0,00000	0,00000	0,00000	0,00962	0,00000	0,00000	0,00000	0,00000	0,00000	0,00000	0,00000
TRGV8*01TRGJ1*01	CATWDR#YYKKLF	0,00000	0,00000	0,00000	0,00939	0,00000	0,00000	0,00000	0,00000	0,00000	0,00000	0,00000
TRGV8*01TRGJP1*01	CATWLSG#WFKIF	0,00000	0,00000	0,00000	0,00892	0,00000	0,00000	0,00000	0,00000	0,00000	0,00000	0,00000
TRGV4*02TRGJ1*02	CATWDLLKSEKKLF	0,00000	0,00000	0,00000	0,00869	0,00000	0,00000	0,00000	0,00000	0,00000	0,00000	0,00000
TRGV5*01TRGJ1*02	CATWDRPNYYKKLF	0,00000	0,00000	0,00000	0,00861	0,00000	0,00000	0,00000	0,00000	0,00000	0,00000	0,00000
TRGV3*01TRGJ1*01	CATWDRPGDYKKLF	0,00000	0,00000	0,00000	0,00854	0,00000	0,00000	0,00000	0,00000	0,00000	0,00000	0,00000
TRGV10*02TRGJ1*01	CAAWP#YYKKLF	0,00000	0,00000	0,00000	0,00776	0,00000	0,00000	0,00000	0,00000	0,00000	0,00000	0,00000
TRGV8*01TRGJ1*02	CATWDYP#YYKKLF	0,00000	0,00000	0,00000	0,00776	0,00000	0,00000	0,00000	0,00000	0,00000	0,00000	0,00000
TRGV3*02TRGJ2*01	CATWDSTYKF#KLQKKLF	0,00000	0,00000	0,00000	0,00745	0,00000	0,00000	0,00000	0,00000	0,00000	0,00000	0,00000
TRGV11*01TRGJP2*01	CACWIR#SSDWIKTF	0,00000	0,00000	0,00000	0,00691	0,00000	0,00000	0,00000	0,00000	0,00000	0,00000	0,00000
TRGV4*01TRGJ2*01	CATW#ICYKKLF	0,00000	0,00000	0,00000	0,00582	0,00000	0,00000	0,00000	0,00000	0,00000	0,00000	0,00000
TRGV3*01TRGJ1*02	CATWDRHLG#KLF	0,00000	0,00000	0,00000	0,00000	0,24704	0,17527	0,44838	0,05591	0,10669	0,28287	0,02206
TRGV10*02TRGJ1*02	CAAWDY#RELF	0,00000	0,00000	0,00000	0,00000	0,02262	0,00273	0,00574	0,00939	0,01707	0,01955	0,00000
TRGV1*01TRGJ1*02	CATWDRHLG#KLF	0,00000	0,00000	0,00000	0,00000	0,01030	0,03495	0,02218	0,00064	0,01540	0,01729	0,00279
TRGV11*01TRGJP1*01	CACWIRHDGATGWFKIF	0,00000	0,00000	0,00000	0,00000	0,00783	0,00036	0,00751	0,01336	0,01072	0,02034	0,00527
TRGV10*02TRGJP1*01	CAAWDP#TGWFKIF	0,00000	0,00000	0,00000	0,00000	0,00619	0,00728	0,00156	0,00000	0,00000	0,00000	0,00000
TRGV2*03TRGJP2*01	CATWDGP#SDWIKTF	0,00000	0,00000	0,00000	0,00000	0,00351	0,00481	0,00156	0,00370	0,00267	0,02576	0,00000
TRGV5P*01TRGJ2*01	CAS#YYKKLF	0,00000	0,00000	0,00000	0,00000	0,00208	0,01253	0,00000	0,00000	0,00000	0,00000	0,00000
TRGV3*01TRGJP2*01	CATWDRR#SDWIKTF	0,00000	0,00000	0,00000	0,00000	0,00197	0,00000	0,00000	0,00815	0,01966	0,00806	0,00000
TRGV10*02TRGJ1*02	CAAWDHKKLF	0,00000	0,00000	0,00000	0,00000	0,00148	0,04574	0,00156	0,00003	0,02690	0,00000	0,00000
TRGV4*02TRGJ1*01	CATWDRLKLNYYKKLF	0,00000	0,00000	0,00000	0,00000	0,00099	0,00911	0,00000	0,00000	0,00000	0,00000	0,00000
TRGV4*02TRGJ1*02	CATWDDYYKKLF	0,00000	0,00000	0,00000	0,00000	0,00000	0,01912	0,00000	0,00000	0,00000	0,00000	0,00000
TRGV10*01TRGJP1*01	CAAWG#GWFKIF	0,00000	0,00000	0,00000	0,00000	0,00000	0,01663	0,00000	0,00000	0,00000	0,00000	0,00000
TRGV8*01TRGJ1*02	CATWD#SQKLF	0,00000	0,00000	0,00000	0,00000	0,00000	0,00921	0,00000	0,00000	0,00000	0,00000	0,00000
TRGV3*01TRGJ1*01	CATWDRPM#YKKLF	0,00000	0,00000	0,00000	0,00000	0,00000	0,00139	0,06202	0,00315	0,00746	0,04942	0,00000
TRGV3*01TRGJ1*02	CATWDGDPLDYYKKLF	0,00000	0,00000	0,00000	0,00000	0,00000	0,00000	0,01428	0,00000	0,00000	0,00000	0,00000
TRGV2*01TRGJ1*02	CATWDGDPLDYYKKLF	0,00000	0,00000	0,00000	0,00000	0,00000	0,00000	0,01152	0,00000	0,00000	0,00000	0,00000
TRGV5*01TRGJ1*02	CATWDRPS#KLF	0,00000	0,00000	0,00000	0,00000	0,00000	0,00000	0,01055	0,00000	0,00000	0,00000	0,00000
TRGV8*01TRGJP2*01	CATWE*PGGDWIKTF	0,00000	0,00000	0,00000	0,00000	0,00000	0,00000	0,00899	0,00000	0,00000	0,00000	0,00000
TRGV8*01TRGJ1*02	CATWD#NYYKKLF	0,00000	0,00000	0,00000	0,00000	0,00000	0,00000	0,00290	0,00012	0,01398	0,00436	0,00629
TRGV5*01TRGJ1*01	CATWDGA#NYYKKLF	0,00000	0,00000	0,00000	0,00000	0,00000	0,00000	0,00000	0,00321	0,01027	0,00545	0,00101

mutational status (NGS)	gene	p.AA	Patient tumor	PDX24-P1_Spleen	PDX24-P3_spleen
			variant allele frequency (%)		
	RHOA	p.G17V	0,18000	0,00000	0,03000
	TET2	p.Q1526X	0,32900	0,95000	0,98500
**TCRbeta repertoire**	**beta gene rearrangement**	**Junction AA**	**% of rearrangement withing all TCRB gene rearrangements**		
	TRBV12-5*01TRBJ2-2*01	CASGITGT#NTGELFF	0,64291	0,00550	0,12705
	TRBV21-1*01TRBJ2-7*01	CASSEGRSYEQYF	0,05882	0,00000	0,00000
	TRBV15*02TRBJ1-6*01	CATSRVLGSPLHF	0,03077	0,00000	0,00000
	TRBV19*01TRBJ2-5*01	CASSMEGGGETQYF	0,02816	0,00000	0,00000
	TRBV6-6*02TRBJ1-6*01	CASSYGREVAYNSPLHF	0,02165	0,00000	0,00000
	TRBV6-1*01TRBJ1-5*01	CASSEWTASNQPQHF	0,02102	0,00000	0,00000
	TRBV29-1*02TRBJ2-5*01	CSVERGPGETQYF	0,02091	0,00000	0,00000
	TRBV7-2*02TRBJ1-2*01	CASSFSGGYGGYTF	0,02012	0,00000	0,00000
	TRBV21-1*01TRBJ1-1*01	CAAKGGWESTGTEAFF	0,00000	0,43502	0,45017
	TRBV7-9*01TRBJ2-7*01	CASSLGLAGYDEQYF	0,00000	0,52009	0,24837
	TRBV6-5*01TRBJ2-1*01	CASTSGGAGGSYNEQF F	0,00000	0,00000	0,09569
	TRBV25-1*01TRBJ2-7*01	CASSKDRGASYEQYF	0,00000	0,00000	0,05453
	TRBV11-3*02TRBJ2-7*01	CASSPPGLG#FYEQYF	0,00000	0,00000	0,00695
	TRBV12-4*01TRBJ2-2*01	CASSTTGTSVTGELFF	0,00000	0,01570	0,00000
	TRBV28*01TRBJ1-4*01	CASTSGGSNEKLFF	0,00000	0,00635	0,00000
	TRBV5-5*01TRBJ2-7*01	CASILGLAGYDEQYF	0,00000	0,00550	0,00000
**TCRalpha repertoire**	**alpha gene rearrangement**	**Junction AA (around half non coding)**	**% of rearrangement withing all TCRA gene rearrangements**		
	TRAV13-1*01TRAJ6*01	CAASKTSGGSYIPTF	0,34708	0,00765	0,01173
	TRAV23/DV6*01TRAJ4*01	CAPRLLDE#NKLIF	0,16209	0,00063	0,04442
	TRAV17*01TRAJ26*01	CATVTDDNYGQNFVF	0,04488	0,00000	0,00000
	TRAV30*05TRAJ42*01	CGTEKPLSGGSQGNLIF	0,02432	0,00000	0,00000

TRAV5*01TRAJ35*01	CAEG#GFGNVLHC	0,02396	0,00000	0,00000
TRAV16*01TRAJ27*01	CAL#NTNAGKSTF	0,01760	0,00000	0,00000
TRAV8-4*03TRAJ9*01	CAAPRGGGFKTIF	0,01757	0,00000	0,00000
TRAV14/DV4*01TRAJ52*01	CATINAGGTSYGKLTF	0,01685	0,00000	0,00000
TRAV26-1*01TRAJ50*01	CIVRVEKTSYDKVIF	0,01652	0,00000	0,00000
TRAV4*01TRAJ45*01	CLVGDMYS	0,01600	0,00000	0,00000
TRAV26-1*01TRAJ48*01	CIVRSRRSNFGNEKLTF	0,01489	0,00000	0,00000
TRAV4*01TRAJ10*01	CLVGDGLTGGGNKLTF	0,01480	0,00000	0,00000
TRAV12-1*01TRAJ48*01	CVVNIGR#FGNEKLTF	0,01317	0,00000	0,00000
TRAV12-3*01TRAJ52*01	CA*#IAGGTSYGKLTF	0,01293	0,00000	0,00000
TRAV12-1*01TRAJ26*01	CVVNDYGQNFVF	0,01163	0,00000	0,00000
TRAV20*01TRAJ48*01	CAVQAFV#GNEKLTF	0,01124	0,00000	0,00000
TRAV9-2*01TRAJ5*01	CALDTGRRALTF	0,01091	0,00000	0,00000
TRAV12-2*01TRAJ38*01	CAMPTQ#CWQQP*ADL	0,01061	0,00000	0,00000
TRAV10*01TRAJ36*01	CVVSDQTGANNLFF	0,00983	0,00000	0,00000
TRAV8-3*02TRAJ45*01	CAVVPS	0,00952	0,00000	0,00000
TRAV1-2*01TRAJ17*01	CAVREW#AAGNKLTF	0,00889	0,00000	0,00000
TRAV17*01TRAJ22*01	CATDDSGSARQLTF	0,00823	0,00000	0,00000
TRAV13-1*01TRAJ7*01	CAASW#GNNRLAF	0,00796	0,00000	0,00000
TRAV1-2*01TRAJ28*01	CAVRH#YS	0,00763	0,00000	0,00000
TRAV12-3*01TRAJ37*01	CAMSFLGSGNTGKLIF	0,00645	0,00000	0,00000
TRAV4*01TRAJ3*01	CLVGGSRSSASKIIF	0,00642	0,00000	0,00000
TRAV13-1*01TRAJ9*01	CAAS#TGGFKTIF	0,00594	0,00000	0,00000
TRAV13-1*01TRAJ53*01	CAIGGSNYKLTF	0,00567	0,00000	0,00000
TRAV14/DV4*02TRAJ49*01	CAMRSPGNQFYF	0,00555	0,00000	0,00000
TRAV4*01TRAJ26*01	CLVGGNYAGQNFVF	0,00470	0,00000	0,00000
TRAV6*02TRAJ20*01	CAPT#SNDYKLSF	0,00374	0,00000	0,00000
TRAV9-2*01TRAJ20*01	CALSTNDYKLSF	0,00335	0,00000	0,00000
TRAV1-1*02TRAJ17*01	CAVCREGAAGNKLTF	0,00295	0,00000	0,00000
TRAV12-1*01TRAJ32*02	CVVNRGGATNKLIF	0,00295	0,00000	0,00000
TRAV23/DV6*02TRAJ26*01	CAASAP#GQNFVF	0,00000	0,00122	0,00000
TRAV13-1*01TRAJ50*01	CAASASKVIF	0,00000	0,00014	0,00000
TRAV13-2*02TRAJ26*01	CADR#YGQNFVF	0,00000	0,00236	0,00000

	TRAV13-2*01TRAJ6*01	CAENMGGSYIPTF	0,00000	0,00017	0,00000
	TRAV38-1*01TRAJ13*01	CAFMKGGYQKVTF	0,00000	0,00028	0,13852
	TRAV35*01TRAJ26*01	CAGRFNYGQNFVF	0,00000	0,02868	0,03309
	TRAV16*01TRAJ49*01	CALR#NTGNQFYF	0,00000	0,39794	0,25122
	TRAV12-3*01TRAJ6*01	CAMESGGSYIPTF	0,00000	0,00199	0,00000
	TRAV14/DV4*01TRAJ22*01	CAMREG#GSARQLTF	0,00000	0,01445	0,00000
	TRAV27*01TRAJ29*01	CAPI#NSGNTPLVF	0,00000	0,00000	0,11737
	TRAV26-1*01TRAJ23*01	CIVRA#NQGGKLIF	0,00000	0,01121	0,02178

TCRgamma repertoire	**gamma gene rearrangement**	**Junction AA (Mostly non coding)**	**% of rearrangement withing all TCRG gene rearrangements**		
	**TRGV10*02TRGJ1*02**	**CAAWEL#KLF**	**0,31587**	**0,00000**	**0,06900**
	**TRGV1*01TRGJ1*02**	**CAT*PSA#KLF**	**0,24948**	**0,00000**	**0,02691**
	TRGV5*01TRGJ1*02	CATWDSPVR#SYYKKLF	0,04296	0,00000	0,00000
	TRGV8*01TRGJ1*01	CATWD#NYYKKLF	0,03422	0,00000	0,00000
	TRGV2*03TRGJ1*01	CAA#KKLF	0,03195	0,00000	0,00000
	TRGV8*01TRGJP2*01	CATWYSSDWIKTF	0,02504	0,00000	0,00000
	TRGV10*02TRGJ1*01	CAAWD#LIRLF	0,02251	0,00000	0,00000
	TRGV9*01TRGJ1*01	CALWGAYYKKLF	0,01898	0,00000	0,00000
	TRGV3*02TRGJ1*02	CATWDKGKLF	0,01788	0,00000	0,00000
	TRGV5*01TRGJ1*02	CATWDRKVRKLF	0,01781	0,00000	0,00000
	TRGV3*01TRGJP1*01	CATWDRPRLE#TTGWFKIF	0,01711	0,00000	0,00000
	TRGV3*02TRGJ1*02	CATWDYHYKKLF	0,01638	0,00000	0,00000
	TRGV10*02TRGJ1*01	CAAWD#YYKKLF	0,01597	0,00000	0,00000
	TRGV10*02TRGJP1*01	CAAWDR#TGWFKIF	0,01307	0,00000	0,00000
	TRGV10*02TRGJ1*02	CAAFLGW#YKKLF	0,01168	0,00000	0,00000
	TRGV9*01TRGJ1*02	CALWEGL#YYKKLF	0,00999	0,00000	0,00000
	TRGV5*01TRGJ1*02	CATWDRLLGM#KKLF	0,00885	0,00000	0,00000
	TRGV10*02TRGJ1*02	CAAWALYYKKLF	0,00676	0,00000	0,00000
	TRGV2*02TRGJP2*01	CATWA#YSDWIKTF	0,00672	0,00000	0,00000
	TRGV9*01TRGJP*01	CALWEVQELGKKIKVF	0,00599	0,00000	0,00000
	TRGV2*02TRGJ1*02	CATWDIPE#YYKKLF	0,00580	0,00000	0,00000
	TRGV3*02TRGJ1*02	CATWDSPMTYYKKLF	0,00558	0,00000	0,00000
	TRGV9*01TRGJ1*02	CALWEATRYYKKLF	0,00441	0,00000	0,00000

TRGV2*02TRGJP1*01	CATWDDGWFKIF	0,00224	0,00000	0,00000
TRGV8*01TRGJ1*01	CARGPYYKKLF	0,00000	0,00025	0,00002
TRGV8*01TRGJ1*02	CATGPYYKKLF	0,00000	0,68119	0,35685
TRGV4*02TRGJ1*01	CATWDAPGG#YYKKLF	0,00000	0,29223	0,22492
TRGV4*02TRGJ1*02	CATWDGG#KLF	0,00000	0,01219	0,00000
TRGV4*01TRGJ1*02	CATWDGLNYYKKLF	0,00000	0,01642	0,00000
TRGV2*03TRGJ1*01	CATWDGNHSNYYKKLF	0,00000	0,02336	0,00000
TRGV1*01TRGJ2*01	CATWDRGG#MRKLF	0,00000	0,00000	0,00233
TRGV8*01TRGJ1*02	CATWDRP#YKKLF	0,00000	0,00000	0,02201
TRGV8*01TRGJ1*02	CATWDRRV#YYKKLF	0,00000	0,00000	0,13843
TRGV4*02TRGJ1*01	CATWR*#KKLF	0,00000	0,00000	0,01650

			Patient tumor	PDX26-P1 Spleen	PDX26-P1 Lung	DX26-P1 Skin	PDX26-P2 Spleen	PDX26-P2 Lung	PDX26-P2 Skin
**mutational status (NGS)**	**gene**	**p.AA**	**variant allele frequency (%)**						
	IDH2	p.R172K	0,107	0,01	ND	ND	ND	ND	ND
	PLCG1	p.S520F	0,041	0,039	ND	ND	ND	ND	ND
	RHOA	p.G17V	0,14	0,412	ND	ND	ND	ND	ND
	TET2	p.W954X	0,199	0,388	ND	ND	ND	ND	ND
	TET2	p.K1818fs	0,22	0,326	ND	ND	ND	ND	ND
	DNMT3A	p.V296M	0,16	0,331	ND	ND	ND	ND	ND
**TCRbeta repertoire**	**beta gene rearrangement**	**Junction AA**	**% of rearrangement withing all TCRB gene rearrangements**						
	**TRBV9*03TRBJ1-1*01**	**CASSKTGRNTEAFF**	**0,6988653**	**0,9000842**	**0,82498743**	**0,96906202**	**0,9854183**	**0,928255072**	**0,98964346**
	TRBV9*03TRBJ2-1*01	CASNKGSGGGPHEQFF	0,0664824	0	0	0	0	0	0
	TRBV4-1*02TRBJ2-7*01	CASSIFTSGSYEQYF	0,0531714	0	0,0047058	0	0	0	0
	TRBV4-2*01TRBJ1-1*01	CASSKTGRNTEAFF	0,0154204	0,02872112	0,02004454	0,00126564	0,0046126	0,001523955	0
	TRBV16*01TRBJ1-5*01	CASSRQSNQPQHF	0	0,03985406	0,0979237	0	0	0	0
	TRBV19*03TRBJ1-5*01	CASSWSAGSNQPQHF	0	0,00411638	0,00416697	0	0	0	0
	TRBV15*02TRBJ2-5*01	CATSRALKETQYF	0	0,00215174	0	0	0	0	0
	TRBV4-3*01TRBJ1-2*01	CASSQTLHKGSPGGYTF	0	0,00196464	0	0	0	0	0
	TRBV12-4*01TRBJ2-5*01	CASSFLLGTQETQYF	0	0,00187108	0,02144551	0	0	0	0
	TRBV12-4*01TRBJ2-5*01	CASRTGGLVETQYF	0	0	0	0	0	0,055886275	0
	TRBV3-1*01TRBJ1-2*01	CASSKTGRNTEAFFGQGTRLTVVGKTFFRFFCRSVTGKSGSTVSLLEWLYSYV#NYGYTF	0	0	0,00901645	0	0	0	0
**TCRalpha repertoire**	**alpha gene rearrangement**	**Junction AA (around half non coding)**	**% of rearrangement withing all TCRA gene rearrangements**						
	**TRAV29/DV5*03TRAJ32*02**	**CAASPGGATNKLIF**	**0,3903607**	**0,46730382**	**0,47240102**	**0,41798646**	**0,5388405**	**0,424540779**	**0,46445316**
	**TRAV19*01TRAJ11*01**	**CALSEPH#SGYSTLTF**	**0,2719334**	**0,45372233**	**0,38504768**	**0,52102911**	**0,4427437**	**0,519030125**	**0,39664662**
	TRAV3*01TRAJ45*01	CAVRD#S	0,0513392	0	0	0	0	0	0
	TRAV9-1*01TRAJ20*01	CALSDPRDDYKLSF	0,0431907	0	0	0	0	0	0
	TRAV29/DV5*01TRAJ52*01	CAASSINAGGTSYGKLTF	0,0428984	0	0	0	0	0	0
	TRAV26-1*01TRAJ31*01	CIVRVC#NARLMF	0,0355538	0	0	0	0	0	0
	TRAV14/DV4*02TRAJ37*02	CAMRRWSNTGKLIF	0,0318632	0	0	0	0	0	0
	TRAV12-3*01TRAJ42*01	CAMSGYGGSQGNLIF	0,0223262	0	0	0	0	0	0
	TRAV26-1*01TRAJ29*01	CIVRDFPGNTPLVF	0,0204261	0	0	0	0	0	0
	TRAV17*01TRAJ58*01	CAPIV*ETSGSRLTF	0,0169547	0	0	0	0	0	0
	TRAV1-2*01TRAJ27*01	CAVRDTNAGKSTF	0,0161508	0	0	0	0	0	0
	TRAV2*01TRAJ44*01	GTASKLTF	0,0120583	0	0	0	0	0	0
	TRAV9-2*01TRAJ57*01	CALG#TQGGSEKLVF	0,0071254	0	0	0	0	0	0
	TRAV41*01TRAJ58*01	CAVTGGETSGSRLTF	0,0024847	0	0	0	0	0	0
	TRAV12-1*01TRAJ8*01	CVVNKGFQKLVF	0,0007308	0	0	0	0	0	0
	TRAV41*01TRAJ58*01	CAVTVGETSGSRLTF	0,0002558	0	0	0	0	0	0
	TRAV29/DV5*02TRAJ32*02	CAASAGGATNKLIF	0,0002192	0	0,00011268	6,5716E-05	7,972E-05	4,40852E-05	0,00015289
	TRAV13-1*01TRAJ10*01	CAASKPGGGNKLTF	0	0,00533199	0	0	0	0	0
	TRAV23/DV6*01TRAJ43*01	CAASLA#NDMRF	0	0,00704225	0,04095832	0	0	0,013328435	0,10197737

	TRAV29/DV5*01TRAJ32*02	CAASPRGATNKLIF	0	0	2,8169E-05	0	7,972E-05	0	0,00012741
	TRAV23/DV6*01TRAJ20*01	CAEGGNDYKLSF	0	0	0	0	0	0,01137399	0,02764754
	TRAV17*01TRAJ23*01	CAIMIYNQGGKLIF	0	0	0,0044226	0	0	0	0
	TRAV8-3*01TRAJ40*01	CAVGK#SGTYKYIF	0	0	0,00436626	0	0	0	0
	TRAV20*01TRAJ22*01	CAVQFSGSARQLTF	0	0	0	0,02349346	0	0	0
	TRAV1-1*01TRAJ23*01	CAVRE*#GGKLIF	0	0,00487928	0,00709869	0	0	0	0
	TRAV8-6*01TRAJ52*01	CAVSAKG#AGGTSYGKLTF	0	0,0028672	0,00790152	0	0	0	0
	TRAV1-2*01TRAJ29*01	CAVTSLRNTPLVF	0	0,00125755	0,0018451	0,01058027	0	0	0
	TRAV14/DV4*03TRAJ43*01	CDNNNDMRF	0	0,00150905	1,4085E-05	0	0	0	0
	TRAV12-1*01TRAJ29*01	CVVN#FRNTPLVF	0	0	0	0,0186962	0	0	0
	TRAV12-1*01TRAJ29*01	CVVNRGNTPLVF	0	0	0	0	3,189E-05	0,01334313	0
TCRgamma repertoire	**gamma gene rearrangement**	**Junction AA (Mostly non coding)**	**% of rearrangement withing all TCRG gene rearrangements**						
	TRGV3*02TRGJ1*02	CATC#FKKLF	0,4449704	0,57433186	0,45747671	0,44496173	0,6551707	0,584511993	0,63238213
	TRGV8*01TRGJ1*01	CATWDGAVRGIV#YYKKLF	0,1073809	0	0	0	0	0	0
	TRGV2*01TRGJ1*02	CATWDGRRY#NYYKKLF	0,0575522	0,2532746	0,26282051	0,32083958	0,2654562	0,302088503	0,17158809
	TRGV3*01TRGJ2*01	CATWDRLKVV#YYKKLF	0,0478979	0	0	0	0	0	0
	TRGV9*01TRGJ1*01	CAFR#KKLF	0,0457801	0	0	0	0	0	0
	TRGV2*01TRGJ1*01	CATWDG#FYKKLF	0,0401744	0	0	0	0	0	0
	TRGV2*01TRGJ1*02	CATWASA#FYKKLF	0,0391155	0	0	0	0	0	0
	TRGV8*01TRGJ1*02	CATWDPFYKKLF	0,0301464	0	0	0	0	0	0
	TRGV8*01TRGJ1*02	CATWDR#YYKKLF	0,0256618	0	0	0	0	0	0
	TRGV11*01TRGJ1*02	CACWTNYYKKLF	0,0232326	0	0	0	0	0	0
	TRGV4*01TRGJ1*02	CATWEG*G#GSGTTLVVTGKYRKNTTFPR*V	0,0230458	0	0	0	0	0	0
	TRGV5*01TRGJ1*02	CATWDRV#YYKKLF	0,0153223	0	0	0	0	0	0
	TRGV11*01TRGJ1*01	CACWMA#KKLF	0,0047337	0	0	0	0	0	0
	TRGV10*02TRGJ1*01	CAAWETIP*A#YYKKLF	0,0042977	0	0	0	0	0	0
	TRGV5*01TRGJ1*01	CATWDRHKKLF	0,0035503	0	0	0	0	0	0
	TRGV3*01TRGJ2*01	CGTWDRLKVV#YYKKLF	0,0008097	0	0	0	0	0	0
	TRGV8*01TRGJ2*01	CATCD#KKLF	0,0001246	0,04246696	0,0456803	0,03061627	0,0673023	0,060401158	0,02971464
	TRGV10*02TRGJP2*01	CAAWDPTNAG#SDWIKTF	0	0,02237885	0,03405328	0,00765407	0	0	0,08076923
	TRGV10*02TRGJP2*01	CAEWDPTNAG#SDWIKTF	0	0	0	0	0	0	0,00024814
	TRGV2*03TRGJ1*01	CAT*E#YYKKLF	0	0	0	0	0	0,024607113	0,02158809
	TRGV4*01TRGJ2*01	CATRP#YKKLF	0	0,0086931	0,00521676	0	0	0	0
	TRGV4*02TRGJ1*01	CATWD*SG#YKKLF	0	0,01574156	0	0	0	0	0
	TRGV2*03TRGJ1*02	CATWDDL#YYKKLF	0	0	0,003311	0	0	0	0
	TRGV2*01TRGJP1*01	CATWDER#TGWFKIF	0	0,00334802	0,0001925	0	0	0	0
	TRGV2*01TRGJ1*02	CATWDGH#YYKKLF	0	0,00305433	0	0	0	0	0
	TRGV4*02TRGJ1*02	CATWDGP#NYYKKLF	0	0,01045521	0,0454878	0,00118362	0	0,003701406	0,04937965
	TRGV2*03TRGJ1*02	CATWDGPQVYYYYKKLF	0	0,00070485	0,00402325	0	0	0	0
	TRGV4*01TRGJ2*01	CATWDGPRS#YKKLF	0	0	0,01593902	0,08435256	0	0	0

TRGV4*02TRGJ2*01	CATWDGRG#YYKKLF	0	0	0,00500501	0	0	0	0,00086849
TRGV3*02TRGJ2*01	CATWDIL#ENYKKLF	0	0,00657856	0	0,06170599	0	0	0
TRGV8*01TRGJ2*01	CATWDPGNYYKKLF	0	0,0020558	0,002002	0	0	0	0
TRGV3*01TRGJ1*02	CATWDRLP#KKLF	0	0,01298091	0,03651729	0	0	0	0
TRGV3*01TRGJ1*02	CATWDRP#*YKKLF	0	0	0,01459151	0	0	0	0
TRGV3*01TRGJ1*02	CATWDRQWPKKLF	0	0,01350954	0,01871102	0,0338515	0	0	0
TRGV5*01TRGJ1*02	CATWDRRRY#NYYKKLF	0	0	5,775E-05	7,8908E-05	0,0008622	0	6,2035E-05
TRGV2*01TRGJP2*01	CATWDVHSSDWIKTF	0	0	0	0	0	0,005831266	0
TRGV2*01TRGJ2*01	CATWDVSYYKKLF	0	0	0,00456225	0	0	0	0
TRGV8*01TRGJ1*01	CATWEA#KNYKKLF	0	0	0,00639101	0	0	0	0
TRGV10*02TRGJP2*01	YAAWDPTNAG#SDWIKTF	0	0	0	0	0	0	0,00037221
